# Morphology and material composition of raptorial foreleg cuticles in praying mantises *Gongylus gongylodes* and *Sphodromantis lineola*

**DOI:** 10.1038/s41598-025-06427-6

**Published:** 2025-06-20

**Authors:** Timo Zeimet, Stanislav N. Gorb, Wencke Krings

**Affiliations:** 1 Department of Electron Microscopy, Institute of Cell and Systems Biology of Animals, University of Hamburg, Martin-Luther-King-Platz 3, 20146 Hamburg, Germany; 2 Section Mammalogy and Palaeoanthropology, Leibniz Institute for the Analysis of Biodiversity Change, Martin-Luther-King-Platz 3, 20146 Hamburg, Germany; 3 Department of Functional Morphology and Biomechanics, Zoological Institute, Kiel University, Am Botanischen Garten 1–9, 24118 Kiel, Germany; 4 Department of Cariology, Endodontology and Periodontology, Leipzig University, Liebigstraße 12, 04103 Leipzig, Germany

**Keywords:** Young’s modulus, Hardness, Elemental composition, Nanoindentation, Biomechanics, Cuticle, Prey capture, Structural biology, Biomechanics, Entomology

## Abstract

Praying mantises (Mantodea, Insecta) are capable of capturing larger invertebrates and also smaller vertebrates with their raptorial forelegs. Forelegs may exhibit morphological adaptations based on the type of prey they prefer (e.g., softer or harder invertebrates). Since the functionality of a structure is also influenced by its material composition, it could be tailored to match the prey as well. To pave the way for deeper studies on trophic adaptations in insects, this study investigates the morphology, material composition and mechanical properties of the raptorial forelegs of two species of praying mantises with different prey preferences. *Sphodromantis lineola* favours harder and larger invertebrates, such as cockroaches (Blattaria), whereas *Gongylus gongylodes* feeds on softer and smaller invertebrates like flies (Diptera). Both males and females were examined in both species to assess potential sexual dimorphism. The results suggest that the morphology of the raptorial forelegs, the arrangement and tiltability of the spines, and the material mechanical properties of the leg cuticle could potentially be related to the preferred prey type. Autofluorescence documentations reflected the presence of micro gradients in the spines, which are related to their mechanical properties. However, pigmentation in the structures of *G. gongylodes* seemed to corrupt the autofluorescence, complicating comparisons between the species. Elemental analysis confirmed the presence of traces of transition and alkaline earth metals in the raptorial forelegs, though no significant species-specific differences and no correlations to the mechanical properties were detected.

**Supplementary Information:**

The online version contains supplementary material available at 10.1038/s41598-025-06427-6.

## Introduction

The majority of all scientifically described species belong to the invertebrates. The species-richness goes hand in hand with various evolutionary mechanisms that have evolved to support and protect their body structures. These include, for example, an internal calcified skeleton, as seen in Echinodermata, or spiculae, which can be composed of calcite, calcium carbonate, or silicates, providing structural support to Porifera. In arthropods, the exoskeleton is composed of a chitin-proteins matrix, which can also contain minerals. The number of arthropod species accounts for over 80% of known animal species, rendering them a significant part of the planet’s biodiversity^1^. This high biodiversity can be explained, among other factors, by the variety of ecological niches occupied by the arthropod taxa. Among other factors to reduce competition^2^, specialization to specific food sources can give species or individual organisms, an advantage ^3^. An example of competition avoidance within a species is in the holometabolic life cycle of Lepidoptera: larvae are usually specialized in feeding on plant parts, while adults (imagoes) feed on nectar, ensuring that different life stages do not compete for the same resources.

Different feeding strategies in Arthropoda, in the form of trophic specialization, are often related to adaptations in the corresponding mouthparts. In previous studies, the focus was mainly on the morphology of these structures [e.g.,^4–6^]. However, beyond form, the material properties also play a crucial role in function and are increasingly becoming the focus of research [for a comprehensive review, see^7^]. This includes research on the material properties of the mouthpart cuticles of flies^8^, ant lions^9^, Trichoptera larvae^10^, butterflies^11,12^, ants^13–16^, cicada^17^, lady birds^18^, mantises^19^, locusts^20^, true spiders^21^, scorpions^22^, and false scorpions^23^.

Cuticle material properties are influenced by several factors, including (a) the orientation, density, or dimensions of chitin fibres [e.g.,^24^], (b) the incorporation of ions that bind to proteins and cross-link the fibres^7,9,13,16,20,21,25–38^, (c) the degree of mineralization between fibres [e.g.,^39,40^], or (d) varying degrees of tanning of the chitin and the presence of proteins like resilin [see e.g.,^9,10,24,41–45^]. These factors affect local mechanical properties, such as hardness and flexibility, as well as the cuticle’s ability to self-repair, thus determining its function or multifunctionality^7,24,42,46,47^.

Mantises (Mantodea) are capable of capturing not only well-defended insects, but also smaller vertebrates, such as lizards or birds^48^. Raphidiomimoidea^2^ and some Arachnida are able to do this as well, but mantises achieve it without using venom to subdue their prey. Instead, they pursuit and ambush their prey and use their first pair of forelegs, which have evolved into raptorial legs, to quickly seize, immobilize, and fix their catch^49–54^. For example, *Bolbe pygmaea*, a mantis species that grows only up to 1 cm in length, is capable of hunting insects and small invertebrates^55^.

Interestingly, similar sized species, such as *Sphodromantis lineola* and *Gongylus gongylodes*, have different food preferences. *G. gongylodes* prefers flies (Diptera), while *S. lineola* hunts not only flies, but also more defensive insects like cockroaches (Blattaria) and crickets (*Acheta*). This prey specialization could, in addition to morphological adaptations, also have influence on the material properties of the raptorial apparatus. The higher forces, acting on *S. lineola*’s raptorial apparatus when capturing larger or more defensive prey, might be dissipated through the chitinious material to prevent structural damage, such as failure of the foreleg spines. In contrast, such an adaptation may not be necessary for *G. gongylodes*, given its preference for less defensive prey.

In a previous study^19^, we have already tested the mandibles of these species and some of these specimens, tested here, for potential adaptations to the preferred prey. We determined that *S. lineola* possess harder and stiffer mandible cutting edges than *G. gongylodes* and found that the mechanical properties have their origin in the degree of tanning and in the content of magnesium.

We here add onto this study by investigating the raptorial apparatus of *S. lineola* and *G. gongylodes*. The morphologies of the forelegs were documented by various methods. Their autofluorescence signals were recorded and their elemental compositions were tested to reveal potential micro gradients within structure. Additionally, the mechanical properties of some regions were identified. This research shall serve as a basis to further research on higher quantity of mantis species with different feeding strategies in the context of potential adaptations to the preferred prey.

## Materials and methods

### Species profile

#### *Sphodromantis lineola*

**Fig. 1:**
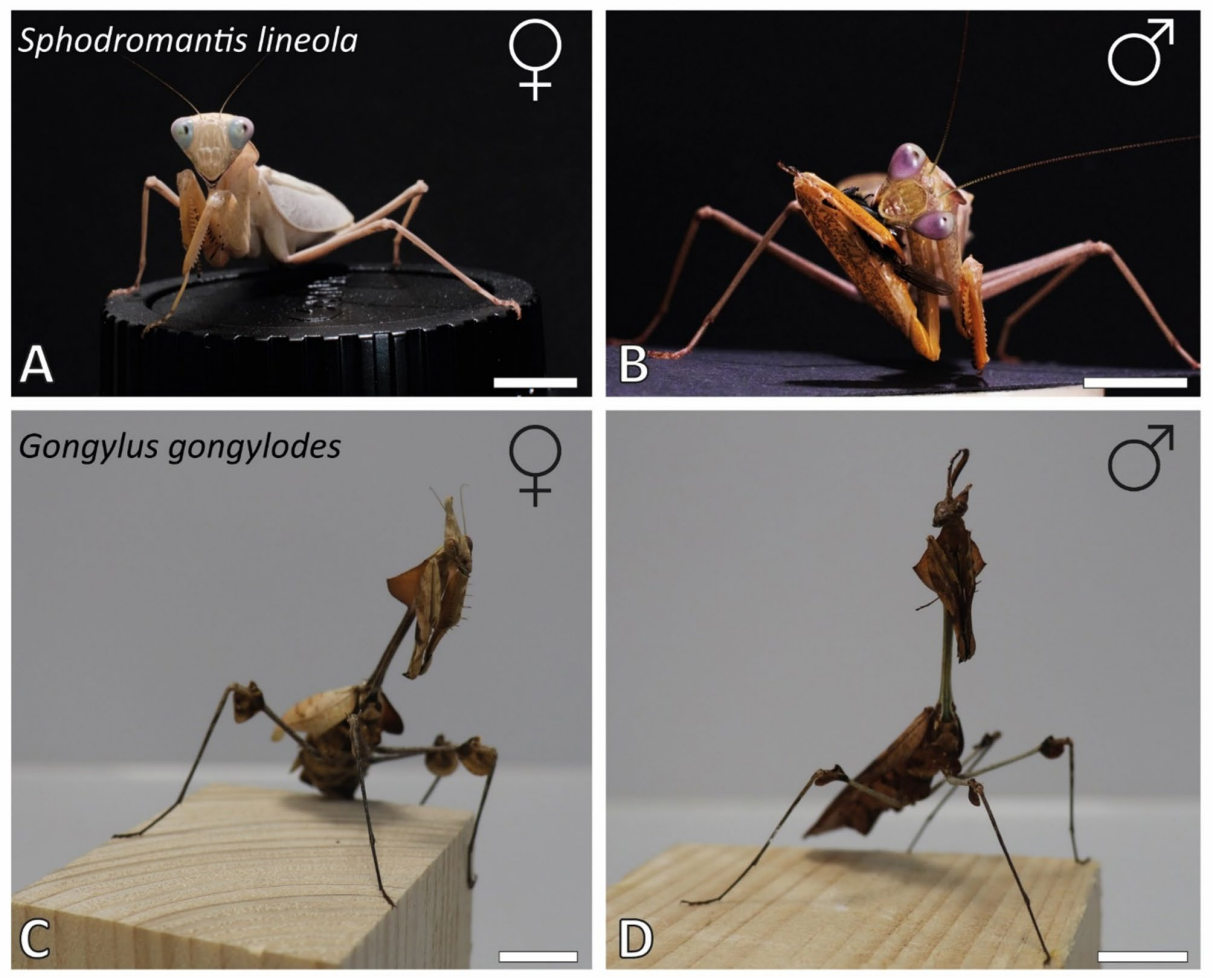
Habitus images. A–B. *Sphodromantis lineola*. (A) Female. (B) Male. C–D. *Gongylus gongylodes*. (C) Female. (D) Male. Images taken from^19^. Scale bars: 12 mm.

*Sphodromantis lineola* (Burmeister, 1838) (Mantodea, Mantidae), commonly known as the Ghana praying mantis, is found across West, East, Central, and parts of Southern Africa (Fig. 1A–B). These mantises inhabit steppe vegetation and can be found in grasses, bushes, and trees. As ambush predators, their diet includes not only flying insects (e.g., Diptera) but also more defensive arthropods such as wasps (Hymenoptera) and cockroaches (Blattodea). Additionally, *S. lineola* is known to hunt small vertebrates (Prete & Cleal, 1996).

The species exhibits a pronounced sexual dimorphism. Females have a more robust body structure, growing up to 9 cm in length, with six visible coxosternites. The wings of adult females reach the tip of the abdomen, but they are limited to short flights of only a few meters. Males, in contrast, grow to a maximum length of 6 cm, have eight visible coxosternites, and possess longer wings, which enable them to fly over greater distances.

Seven adult specimens (three males, four females) were obtained from an online retailer (https://mantidenundmehr.de, M&M Wüst, Mühlheim am Main, Germany).

#### *Gongylusgongylodes*

*Gongylus gongylodes* (Linnaeus, 1758) (Mantodea, Empusidae), commonly known as the wandering violin mantis, is typically found hanging upside down in shrubs and bushes in the wild (Fig. 1C, D). Its primary distribution ranges from India and Thailand to Java, where it inhabits dry, hot regions. This species exhibits a pronounced sexual dimorphism. Males grow to 7–8 cm and are characterized by large, comb-shaped antennae, while females reach a size of 9–10 cm and have short, filamentous antennae. Both sexes possess numerous spines along the tibia and on the medial side of the femur, enabling them to capture both running and flying prey, including very small insects like *Drosophila*. According to^48^, *G. gongylodes* has a wider range of animals it is capable of catching. Breeders as “Mantiden & Mehr” (https://mantidenundmehr.de, M&M Wüst, Mühlheim am Main, Germany) note that flightless insects and cockroaches are unsuitable for this species, which prefers less defensive flying insects, such as Diptera. In videos, we noticed that the animals ate most of the fly except, in most cases, the legs.

One adult male (specimen 08) was obtained from an online retailer (https://mantidenundmehr.de, M&M Wüst, Mühlheim am Main, Germany) and seven adult specimens (four males, four females) were acquired from the private breeder Kai Schütte, Hamburg (Supplementary Table 1).

### Character photos

Alive imagines were photographed using a Canon EOS 80D (with Canon EF-S 18–55 mm f/4–5.6 IS STM and Sigma 105 mm f/2.8 EX DG OS HSM Macro lenses), a FUJIFILM X-T30 II (with Fujifilm Fujinon XF 18–55 mm F2.8–4 R LM OIS lens), and an iPhone 12 (6.1-inch; Apple Inc., Cupertino, USA; 12-megapixel wide-angle camera). The animals were placed on a table and photographed against either a light or dark background.

### Video sequences

To observe the hunting and feeding behavior of the animals, videos were recorded using an iPad Pro (11-inch; Apple Inc., Cupertino, USA; 12-megapixel wide-angle camera) and an iPhone 12 (6.1-inch; Apple Inc., Cupertino, USA; 12-megapixel wide-angle camera). The animals were placed either in a plexiglass container (for juvenile *Gongylus gongylodes*) or on a platform, and were fed with houseflies (*Musca domestica*) or crickets (*Acheta domesticus*) that had been dazed using CO_2_ and cold temperature.

### Stack photography system

For stack photography, the specimens were frozen for two days, prepared, and then dried at room temperature for two weeks. Images of whole specimens were captured from dorsal, ventral, and lateral perspectives. A Canon EOS 6D with a 50 mm lens was used for this purpose, integrated into a photo stacking system. The stacked images were processed using Adobe Lightroom (version 12.2) and Helicon Focus (version 5.3). These images were used for species validation.

### Light microscopy

To document the forelegs, the Keyence Digital Microscope VHX-7000 (KEYENCE, Neu-Isenburg, Germany) was employed. The extremities were photographed at 50x magnification from both the medial and lateral side in dry condition. Terminologies of structures was taken from^56^.

### Categorization of spines and foreleg regions

**Fig. 2:**
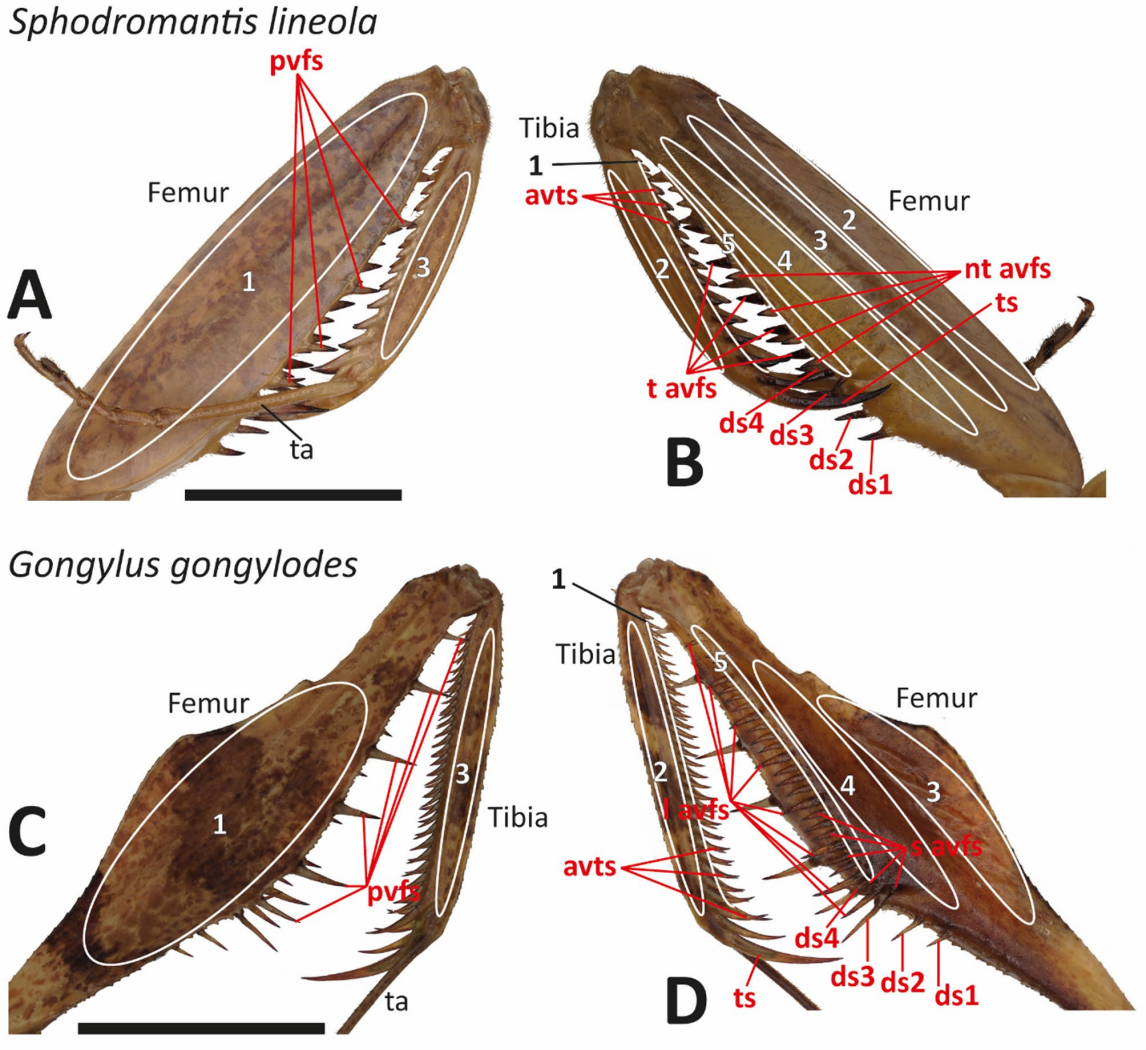
Spines and regions on the femur and tibia were examined. Red letters refer to the spines and white or black numbers as well as the lines to the region type (see Table 1 for the definition of the region types). A–B. *Sphodromantis lineola*, specimen 01, male, right foreleg. (A) Lateral view. (B) Medial view. C–D. *Gongylus gongylodes*, specimen 15, male, right foreleg. (C) Lateral view. (D) Medial view. Abbreviations: avts, anteroventral tibial spines; ds1, 1th discoidal spine; ds2, 2nd discoidal spine; ds3, 3rd discoidal spine; ds4, 4th discoidal spine; l avfs, long anteroventral femoral spines; nt avfs, non-tiltable anteroventral femoral spines; pvfs, posteroventral femoral spines; s avfs, short anteroventral femoral spines; t avfs, tiltable anteroventral femoral spines; ta, tarsus; ts, tibial spur. Scale bars: 6 mm.

Based on morphology and locality, the spines and regions of the forelegs were sorted to certain types. The regions are described in Table 1 and the spines are depicted in Fig. 2.

**Table 1:** Description of the foreleg regions. Abbreviations: avts, anteroventral tibial spines; pvts, posteroventral tibial spines.

Segment	Side	*Sphodromantis lineola*	Region type	*Gongylus* *gongylodes*	Region type
Femur	Lateral	Complete cuticle	1	Complete cuticle	1
Femur	Medial	Along the dorsal cuticle	2	Along the dorsal cuticle	3
Femur	Medial	Medial cuticle	3/4	Medial cuticle	4
Femur	Medial	Ventral cuticle bearing the spines	5	Ventral cuticle bearing the spines	5
Tibia		Cuticle between avts and pvts spines	1	Cuticle between avts and pvts spines	1
Tibia	Medial	Complete cuticle	2	Complete cuticle	2
Tibia	Lateral	Complete cuticle	3	Complete cuticle	3

### Confocal laser scanning microscopy

For confocal laser scanning microscopy (CLSM), we used the following forelegs (see also Supplementary Table 1):

*S. lineola*: specimens 01 (male), 03 (male), 05 (female), 06 (female), always the left foreleg.

*G. gongylodes*, specimens 08 (male), 11 (female), 13 (female), 18 (male), always the right foreleg.

For this examination, the dry forelegs were transferred into 99.5% glycerine (Carl Roth GmbH & Co. KG, Karlsruhe, Germany) and covered with a coverslip. The lateral and medial side of the forelegs were documented. After CLSM imaging, the samples were cleaned with distilled water and transferred back to 70% ethanol.

CLSM allows for high-contrast, pinpoint imaging of reflective or fluorescent regions using lasers. These lasers scan not only the surface, but also penetrate the structure at various depth levels. The reflected signal (in the form of light) is detected through emission filters. Following the protocol by^57^, commonly used for studies of arthropod cuticle, different regions, such as elastic resilin-rich, or highly sclerotized areas can be identified. For the CLSM analysis of the forelegs, a Zeiss LSM 700 (Carl Zeiss Microscopy GmbH, Jena, Germany) was used, employing laser wavelengths of 405, 488, 555, and 639 nm. Emission filters 420–480 nm, ≥ 490 nm, ≥ 560 nm, and ≥ 640 nm were employed.

The master gain was set for Tracks 1–4 at 713, 715, 719, and 707, respectively, with a digital gain of 1.12, 1.25, 1.20, and 1.25. The digital offset remained at 0.00. Laser intensities were set to 60%, 15%, 22%, 45%, respectively. To ensure comparability, the same settings were applied across all images. Maximum intensity projections (MIP) were created using the proprietary Zeiss Efficient Navigation (Zen) software, version 2009 (Carl Zeiss Microscopy GmbH, Jena, Germany; https://www.zeiss.com/microscopy). The following colours were assigned to the autofluorescence elicited by the lasers: laser 405 nm (100% saturation) – blue, 488 nm (100%) – green, 555 nm (50%) – red, and 639 nm (50%) – red.

We additionally separated the channels (T1 blue, T2 green, T3 red 50%, and T4 red 50%) and presented them in grayscale to compare the intensity of the respective autofluorescence. Then, the images were transferred to Adobe Photoshop software CS& (Adobe Inc., San José, USA) and the number of pixels was documented. The number of pixels from channel T1 was used as the basis for this comparison and was set to 1.00 (equal to 100%). The proportions of the autofluorescence signals are depicted in the grayscale CLSM images.

After all measurements, we cut one foreleg of *S. lineola* and one of *G. gongylodes* and CLSM scanned it with the same settings, in order to document the cuticle heterogeneity and the thickness.

### Scanning electron microscopy

The following samples were used for scanning electron microscopy (SEM) imaging (see also Supplementary Table 1):

*S. lineola*: specimens 01 (male), 02 (male), 06 (female), 07 (female), always the right foreleg.

*G. gongylodes*, specimens 09 (male; left foreleg), 11 (female; left foreleg), 14 (female; right foreleg), 15 (male; left foreleg).

Higher-resolution imaging was achieved using a Zeiss LEO 1525 SEM (One Zeiss Drive, Thornwood, New York, USA). Given the sizes of the legs, they were cut at the femur/tibia and coxa/femur joints. The dry parts of the legs were mounted on sample holders using double-sided carbon adhesive tape and coated with platinum (5 μm). After the initial documentation, the samples were rotated, recoated, and documented to obtain views from both the medial and lateral sides.

### Energy-dispersive X-ray spectroscopy

The following samples were used for energy-dispersive X-ray spectroscopy (EDX) (see also Supplementary Table 1):

*S. lineola*: specimens 01 (male), 06 (female), always the right foreleg.

*G. gongylodes*, 14 (female; right foreleg), 15 (male; left foreleg).

To determine the elemental composition of the insect cuticle, EDX detector was utilized. In this method, an electron beam is focused on the sample, generating an emission spectrum that represents the elemental composition. A Zeiss LEO 1525 SEM (One Zeiss Drive, Thornwood, New York, USA) equipped with an Octane Silicon Drift Detector (SDD) (microanalysis system TEAM, EDAX Inc., Mahwah, New Jersey, USA) was employed for the elemental analysis. The EDX system was calibrated using copper (Cu) and aluminium (Al). Due to limitations in this EDX setup, precise resolution of light elements is not possible, rendering the results semi-quantitative.

The samples previously imaged with the SEM and already coated with platinum were used for EDX analysis. The protocol followed the procedures outlined and applied previously^58–63^. The medial side of the tibia and femur of each foreleg was fixed on a slide using double-sided carbon adhesive tape, to prevent the samples from floating in the resin. The samples were encased in metal rings, which were filled with epoxy resin (ECKLI EPOXI WST, RECKLI GmbH, Herne, Germany). After five days of resin polymerization, the slide and adhesive were removed, and the brass rings were polished down with sandpapers (P800, P1200, P3000) to expose the predefined areas for EDX measurement. The polishing process was monitored using a binocular microscope. The exposed surface was then smoothed using aluminium oxide polishing powder (grain size 0.3 μm), distilled water, and a polishing machine (Minitech 233/333, PRESI GmbH, Hagen, Germany). To avoid contamination from sweat or debris, the samples were cleaned in an ultrasonic bath with 70% ethanol for five minutes at 10% intensity and 37 °C before coating them with platinum (5 μm).

The measurements were performed with the same settings (acceleration voltage of 20 kV, working distance 15 mm, lens aperture 60 μm, measurement time for each measurement point 30 s, resolution 137.6 eV).

Detected were and their proportions measured: aluminium (Al), calcium (Ca), carbon (C), chlorine (Cl), copper (Cu), fluorine (F), hydrogen (H), iron (Fe), magnesium (Mg), manganese (Mn), nitrogen (N), oxygen (O), phosphorus (P), platinum (Pt), potassium (K), silicon (Si), sodium (Na), sulphur (S), and zinc (Zn).

Proportions of H, C, N, O, and Al were measured not included in the discussion. H, C, N, and O are constituents of chitin, the primary component of insect cuticle. Al and O can be artefacts from the polishing material. Pt coating was chosen because it offers an advantage over C coating, as Pt is not expected to be present in the sample itself. If Pt was detected, it confirmed a successful measurement. It was noted that one spectrum of Pt overlapped with one of P, and the software could not distinguish between the two elements. Therefore, in the statistical analysis, Pt and P were grouped together as P + Pt. Through EDX measurements of the resin, which did not contain P, the proportion of the Pt coating was determined to be 0.13 ± 0.03 atomic %. The relative atomic composition (atomic %) was used for data analysis.

After measuring the initial region of the forelegs, the steps of polishing, smoothening, cleaning and measuring were repeated until all predefined regions were tested. At least eight measurements were conducted per region. 17 regions (Fig. 2) were defined, and out of the 1503 measurements taken, 1156 were identified manually as reliable and used for analysis. Measurements were excluded if they were outside the predefined regions (e.g., femoral brushes) or those measuring the epicuticle instead of the procuticle.

### Nanoindentation

Nanoindentation was performed on the same samples and at some of the cuticle regions (tibial spur, non-tiltable and tiltable anteroventral femoral spines of *S. lineola*, long and short anteroventral femoral spines of *G. gongylodes*, anteroventral tibial spines, femoral region 4, femoral region 5, tibial region 2) that were analysed in the elemental study. The samples were mounted onto holders following established protocols^59,60^. Each region of interest was tested at room temperature using an SA2 nanoindenter (MTS Nano Instruments, Oak Ridge, USA) equipped with a Berkovich diamond tip. In total, 636 localities were tested, with 344 measurements on *Gongylus gongylodes* and 292 on *Sphodromantis lineola*.

### Statistical analysis

Statistical analyses were conducted with JMP Pro, version 17 (SAS Institute Inc., Cary, NC, 1989–2007; https://www.jmp.com). The data collected through EDX and nanoindentations were tested for normal distribution using the Shapiro-Wilk test. Most of the data was not normally distributed, so the following tests for metric, non-normally distributed data were applied: for comparisons between two groups (between species and between sexes), the Mann-Whitney/Wilcoxon-test (median comparison) and the Kolmogorov-Smirnov test (variance comparison) were used. Correlation coefficients (Pearson correlation test) were also computed with JMP Pro software.

## Results

### Regions of interest

In the studied mantises, the coxa, trochanter, and tarsus did not interact with the prey as documented via videos (see Supplementary Video 1 and 2). When catching or eating the prey animals, the tarsus was pressed against the tibia to get it out of the way [see also^50^ and^64^]. For both the coxa and trochanter, no holding function was observed during feeding in both species. Thus, the investigation of the elemental composition was focused on the femur and tibia.

### Morphology of the raptorial forelegs

**Fig. 3:**
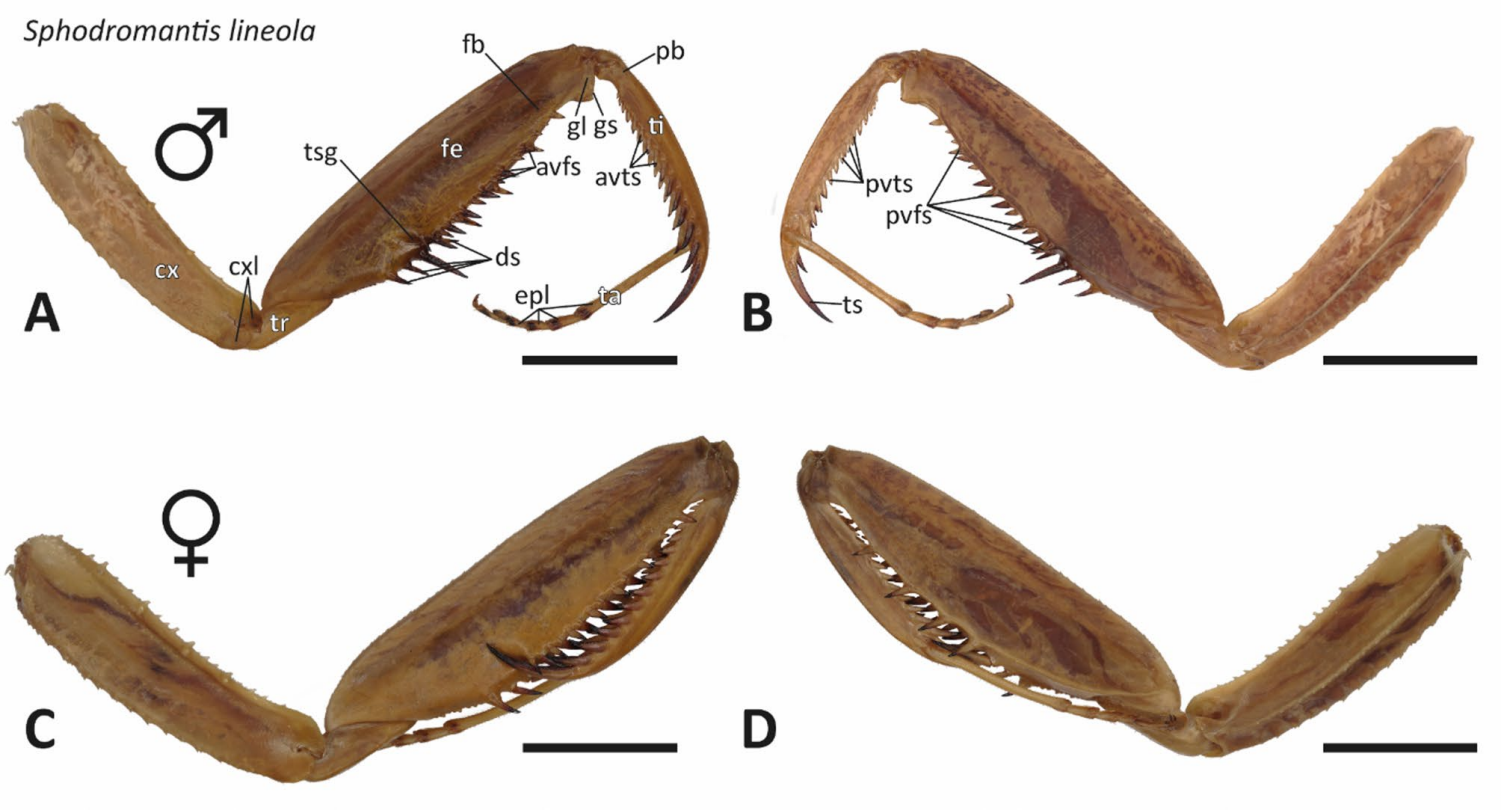
Light microscopic images of the raptorial forelegs. A–B. *Sphodromantis lineola*, specimen 01, male, left foreleg. (A) Medial view. (B) Lateral view. C–D. *S. lineola*, specimen 06, female, left foreleg. (C) Medial view. (D) Lateral view. Abbreviations: avfs, anteroventral femoral spines; avts, anteroventral tibial spines; cx, coxa; cxl, coxal lobes; ds, discoidal spines; epl, euplantulae; fb, femoral brush; fe, femur; gl, genicular lobes; gs, genicular spurs; pb, proximal bend in the tibia; pvfs, posteroventral femoral spines; pvts, posteroventral tibial spines; ta, tarsus; ti, tibia; tr, trochanter; tsg, tibial spur groove. Scale bars: 6 mm.

**Fig. 4:**
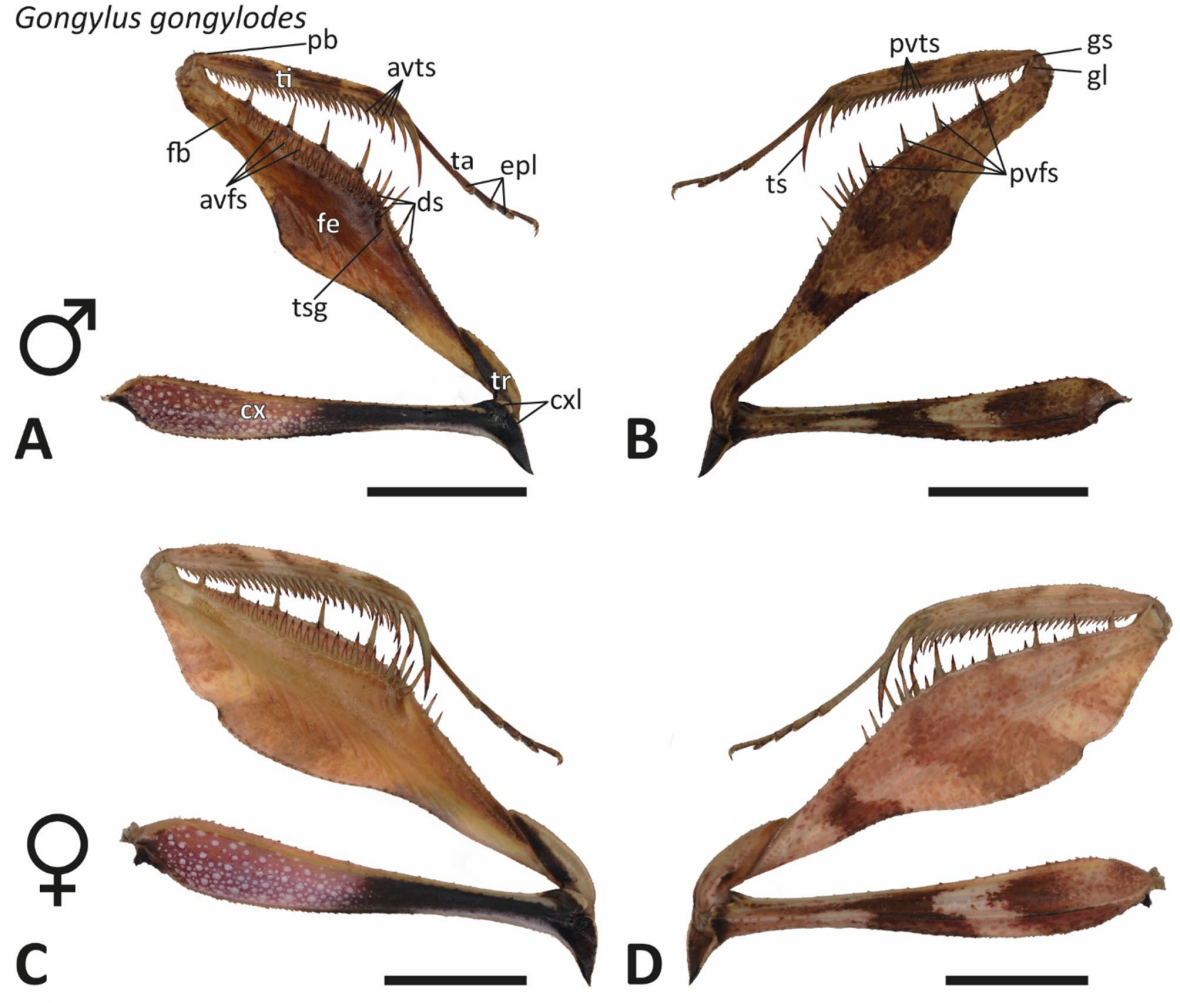
Light microscopic images of the forelegs. A–B. *Gongylus gongylodes*, specimen 15, male, left foreleg. (A) Medial view. (B) Lateral view. C–D. *G. gongylodes*, specimen 14, female, left foreleg. (C) Medial view. (D) Lateral view. Abbreviations: avfs, anteroventral femoral spines; avts, anteroventral tibial spines; cx, coxa; cxl, coxal lobes; ds, discoidal spines; epl, euplantulae; fb, femoral brush; fe, femur; gl, genicular lobes; gs, genicular spurs; pb, proximal bend in the tibia; pvfs, posteroventral femoral spines; pvts, posteroventral tibial spines; ta, tarsus; ti, tibia; tr, trochanter; tsg, tibial spur groove. Scale bars: 6 mm.

The forelegs of *Sphodromantis lineola* and *Gongylus gongylodes* consisted of several segments (Figs. 3 and 4): the coxa (cx), trochanter (tr), femur (fe), tibia (ti), and tarsus (ta). The tarsus, located at the distal end of the tibia, was composed of tarsal segments that contain euplantulae (epl), adhesive structures employed for walking and gripping.

The coxa, connecting the remaining foreleg to the thorax, was longer than the corresponding segment of the hind legs. At the distal end of the coxa, coxal lobes (cxl) almost encircled the joint between the coxa and the trochanter (Figs. 3 and 4) and were elongated in *G. gongylodes* (Fig. 4). The distal end of the trochanter was fused with the femur. Both the femur and tibia bare a series of spines that faced each other when the limb was closed, arranged in distinct rows. On the medial side of the femur the anteroventral femoral spines (avfs) were located, while the lateral side featured the posteroventral femoral spines (pvfs) (Figs. 3 and 4). Proximally, between these two sets of spines, the discoidal spines (ds) were located. The tibial spur groove (tsg) was located between the discoidal spines and the anteroventral femoral spines (Figs. 3 and 4) and bare the tibial spur (ts) when the raptorial apparatus was closed (Supplementary Fig. 1).

Similar to the coxa, the femur-tibia joint was covered by genicular lobes (gl), which also featured genicular spurs (gs). A cleaning brush, the femoral brush (fb), was present on the medial side of the femur, close to the last avfs. The tibia was composed of a proximal bend (pb) and terminated distally in a pronounced ts. The medial side of the tibia possessed a row of anteroventral tibial spines (avts), while the lateral side bare a row of posteroventral tibial spines (pvts).

**Fig. 5:**
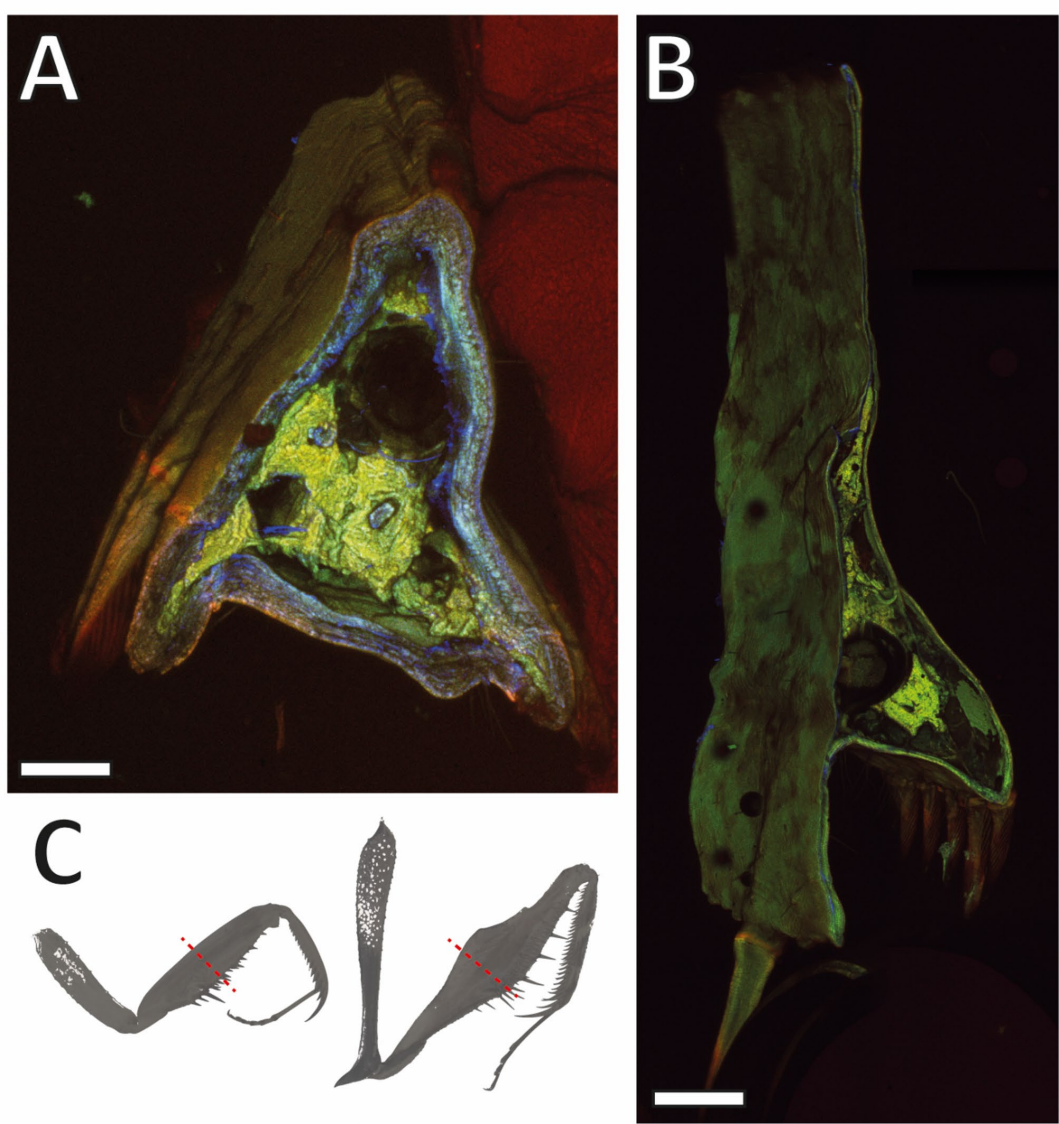
A–B. CLSM images of cross-sections of the raptorial forelegs displaying the cuticle thickness. Images were generated with the same settings. (A) *Sphodromantis lineola*. (B) *Gongylus gongylodes*. (C) Region of the cross-sections. Scale bars: 500 μm.

In both species, male forelegs were smaller (Figs. 3 and 4). *G. gongylodes* displayed more pigmentation, particularly along the medial side of the coxa. Another distinguishing feature was the shape of the femur: *S. lineola* possessed a nearly cylindrical femur, while *G. gongylodes* bare a flattened femur. Additionally, the forelegs of *S. lineola* were thicker than those of *G. gongylodes* (Fig. 5). The femurs of *G. gongylodes* possessed a dorsal inflation.

### Spine morphologies

No differences between males and females in spine shape were observed in each of the species (Figs. 3 and 4). The spines of *S. lineola* were noticeably thicker and shorter compared to those of *G. gongylodes*. Additionally, spines in *S. lineola* were straighter; here, the ts was the only strongly curved spine; with regard to the other spines, only their tips were slightly curved inwards. In *G. gongylodes*, the ts and the avts were curved in a sickle-like manner towards the centre of the tibia. Additionally, the femoral spines in *G. gongylodes* exhibited a slight sickle shape, curving towards the centre of the femur. Spines of *G. gongylodes* exhibited a certain pattern. Specifically, the first four proximal avfs alternated between being shorter or longer, followed by three short spines (in two specimens, we found two short spines) and one long spine distally. In the remaining distal avfs, every fourth spine was elongated.

Similar to floral formulas in plants or dental formulas in mammals, a “spine formula” for mantis forelegs has been previously developed to simplify morphological comparisons [following^56^]. We determined the following spine formula for the two species.

Spine formula for *S. lineola*:

Femur: 4–5 ds / 15–16 avfs / 4 pvfs.

Tibia: 13–15 avts / 10–11 pvts.

Spine formula for *G. gongylodes*:

Femur: 4 ds / 27–31 avfs / 6 pvfs.

Tibia: 26–31 avts / 29–37 pvts.

No differences between males and females in spine quantities were observed in each species. Both species exhibited a nearly identical number of ds. However, an exception was noted in one female *S. lineola* specimen, which possessed a fifth ds positioned proximally to the other four. This suggests some variability, albeit rare, in *S. lineola* compared to *G. gongylodes*. Greater variability was found in the avfs. *S. lineola* bare approximately half the amount of avfs compared to *G. gongylodes*, which also showed more variability in the quantity of these spines. The quantity of pvts was consistent within each species. *G. gongylodes* possessed two more pvts than *S. lineola*. In terms of tibial spines, *S. lineola* possessed 2–5 more avts than pvts, while in *G. gongylodes*, the quantity of avts was 2–11 lower than of the pvts. Overall, *G. gongylodes* displayed twice as many avts and four times as many pvts compared to *S. lineola*.

Some spines in both species were articulable with a joint-like socket at their bases, allowing a tilting (Supplementary Fig. 2). In *S. lineola*, the second and third ds, along with nearly every second avfs, could tilt distally (Supplementary Fig. 2A–B). These spines were thicker and darker than their non-tilting counterparts. The last avfs before the femur-tibia joint, was similar in form and color but not tiltable. In *G. gongylodes*, only the third ds could tilt (Supplementary Fig. 2C–D). Unlike the avfs in *S. lineola*, those in *G. gongylodes* were not tiltable.

### Autofluorescence signals

#### *Sphodromantis lineola*

**Fig. 6:**
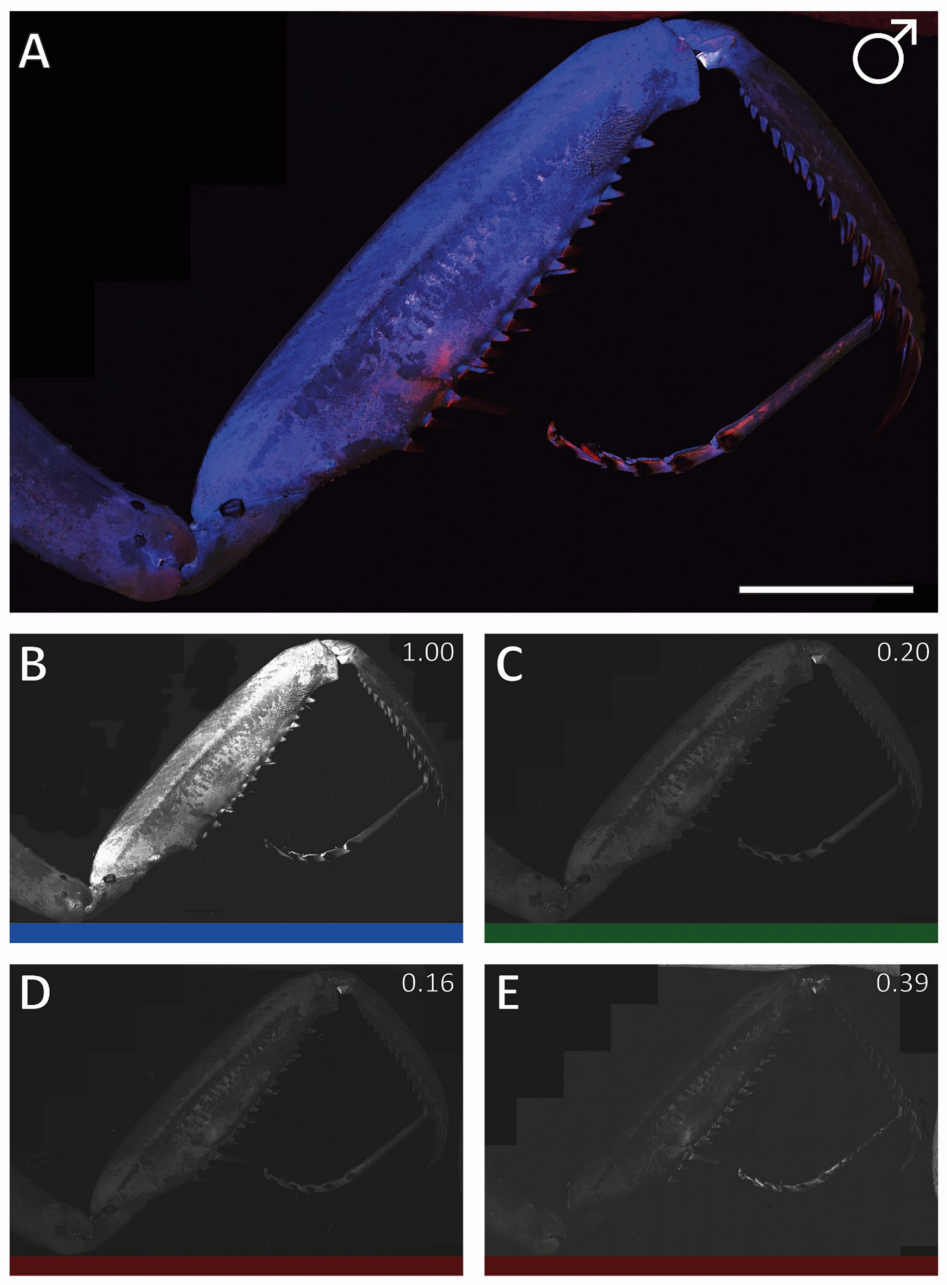
*Sphodromantis lineola*, specimen 01, male, left foreleg, medial view. CLSM image with the same settings as Supplementary Figs. 3–8 and Fig. 7. (A) All channels. (B) Blue (100%). (C) Green (20% compared to blue). (D) Red 50% (16% compared to blue). (E) Red 50% (39% compared to blue). Scale bar: 4 mm.

For the male foreleg, see Fig. 6 and Supplementary Fig. 3, for the female, see Supplementary Fig. 4 and Supplementary Fig. 5. Most of the femur cuticle exhibited a strong blue autofluorescence. In the middle region of the medial side of the femur (region 3, see Figs. 6 and Supplementary Fig. 4), there was a less intense blue band running parallel to the row of spines. The lateral side of the femur (Supplementary Fig. 3 and Supplementary Fig. 5) was more differentiated, displaying five nearly parallel regions: the dorsal region was bright blue, followed by a green area that transitioned to a blue strip in the middle. Towards the ventral region of the foreleg, the blue area then transitioned into a darker region bordered by a green one (Supplementary Fig. 3 and Supplementary Fig. 5).

While the proximal region of the tibia showed a strong blue autofluorescence, most regions exhibited a green autofluorescence in medial view (Figs. 6 and Supplementary Fig. 4). The lateral side of the tibia displayed a strong blue autofluorescence (Supplementary Fig. 3 and Supplementary Fig. 5). From the tarsal joint to the ts, as well as along the dorsal cuticle of the tibia, red autofluorescence was documented.

Comparing the medial sides of the male (Figs. 6 and Supplementary Fig. 3) and female (Supplementary Fig. 4 and Supplementary Fig. 5) forelegs, most of the autofluorescence patterns were similar. However, the male forelegs exhibited a stronger red colour on the medial side. While the tsg showed partially a red autofluorescence signal in males, this was less pronounced in females.

**Fig. 7:**
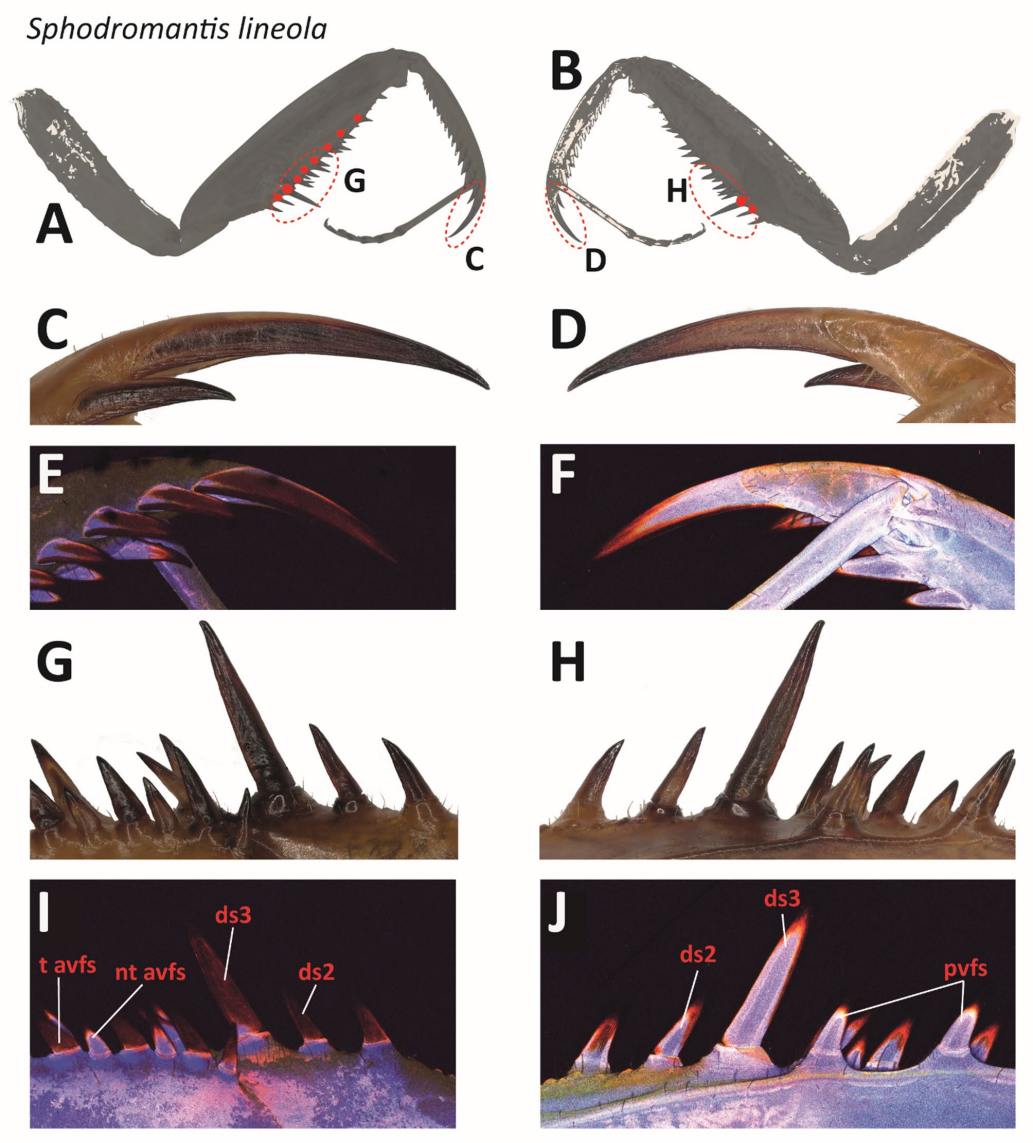
Light microscopy and CLSM images of different spines of *Sphodromantis lineola*, specimen 01, male, left foreleg. A, C, E, G, I. Medial view. B, D, F, H, J. Lateral view. The tiltable spines are high lightened by red dots. C–F. Ts with nt avts. G, I. In the front: t avfs alternating with nt avfs. In the back: (tiltable) ds2 and ds3. H, J. In the front: (non-tiltable) pvfs. In the back: (tiltable) ds2 and ds3. Abbreviations: ds2, 2nd discoidal spine; ds3, 3rd discoidal spine; nt avfs, non-tiltable anteroventral femoral spines; pvfs, posteroventral femoral spines; t avfs, tiltable anteroventral femoral spines; ts, tibial spur.

All spines exhibited a gradual shift in color from red to dark red toward the tips, indicating a higher degree of sclerotization from base to tip (Fig. 7). The extent of red colour varied among the different spine types. The tiltable avfs as well as ds (Fig. 7I) appeared dark red, while the pvfs and the non-tiltable avfs showed a red to dark red autofluorescence only at their tips from their lateral sides (Fig. 7J). The medial sides of the tiltable avfs and the ds exhibited slightly stronger red autofluorescence compared to the pvfs and the non-tiltable avfs (Fig. 7I). No difference was observed between the medial and lateral sides of the avts (Fig. 7E–F). The base of the ts was light blue, transitioning to red and dark red towards the tip (Fig. 7F).

The bases of the tiltable spines exhibited red autofluorescence towards proximal and blue towards distal (Fig. 7J). The bases and sockets of the not-tiltable spines appeared rather uniformly blue (Fig. 7I–J).

#### *Gongylusgongylodes*

**Fig. 8:**
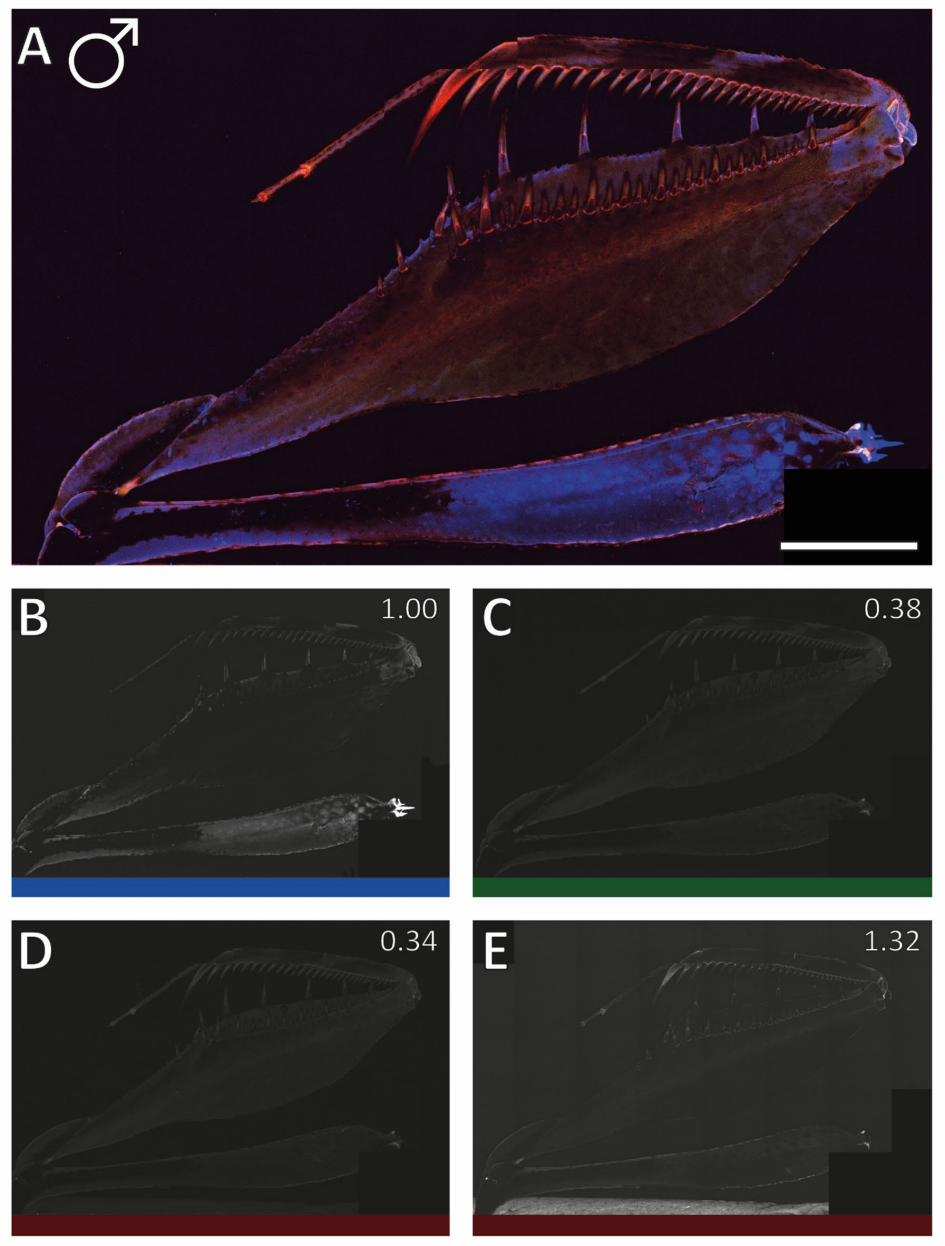
*Gongylus gongylodes*, specimen 15, male, right foreleg, medial view. CLSM image with the same settings as Supplementary Figs. 3–8 and Fig. 6. (A) All channels. (B) Blue (100%). (C) Green (38% compared to blue). (D) Red 50% (34% compared to blue). (E) Red 50% (132% compared to blue). Scale bar: 4 mm.

The structures of *G. gongylodes*, documented with the same settings, exhibited lower autofluorescence signals than the forelegs of *S. lineola*, specifically a notably reduced autofluorescence as answer to the blue laser (for male see Fig. 8 and Supplementary Fig. 6; for female see Supplementary Fig. 7 and Supplementary Fig. 8). Large parts of the forelegs showed a high degree of pigmentation, which likely influenced the autofluorescence signals.

The lateral side of the femur exhibited a slightly stronger blue autofluorescence compared to the medial side (Supplementary Fig. 6 and Supplementary Fig. 8). The tibia also showed a slightly increased blue autofluorescence on its lateral side. Differences between male (Fig. 8 and Supplementary Fig. 6) and female *G. gongylodes* (Supplementary Fig. 7 and Supplementary Fig. 8) in autofluorescence intensity were less pronounced than those observed in *S. lineola*.

**Fig. 9:**
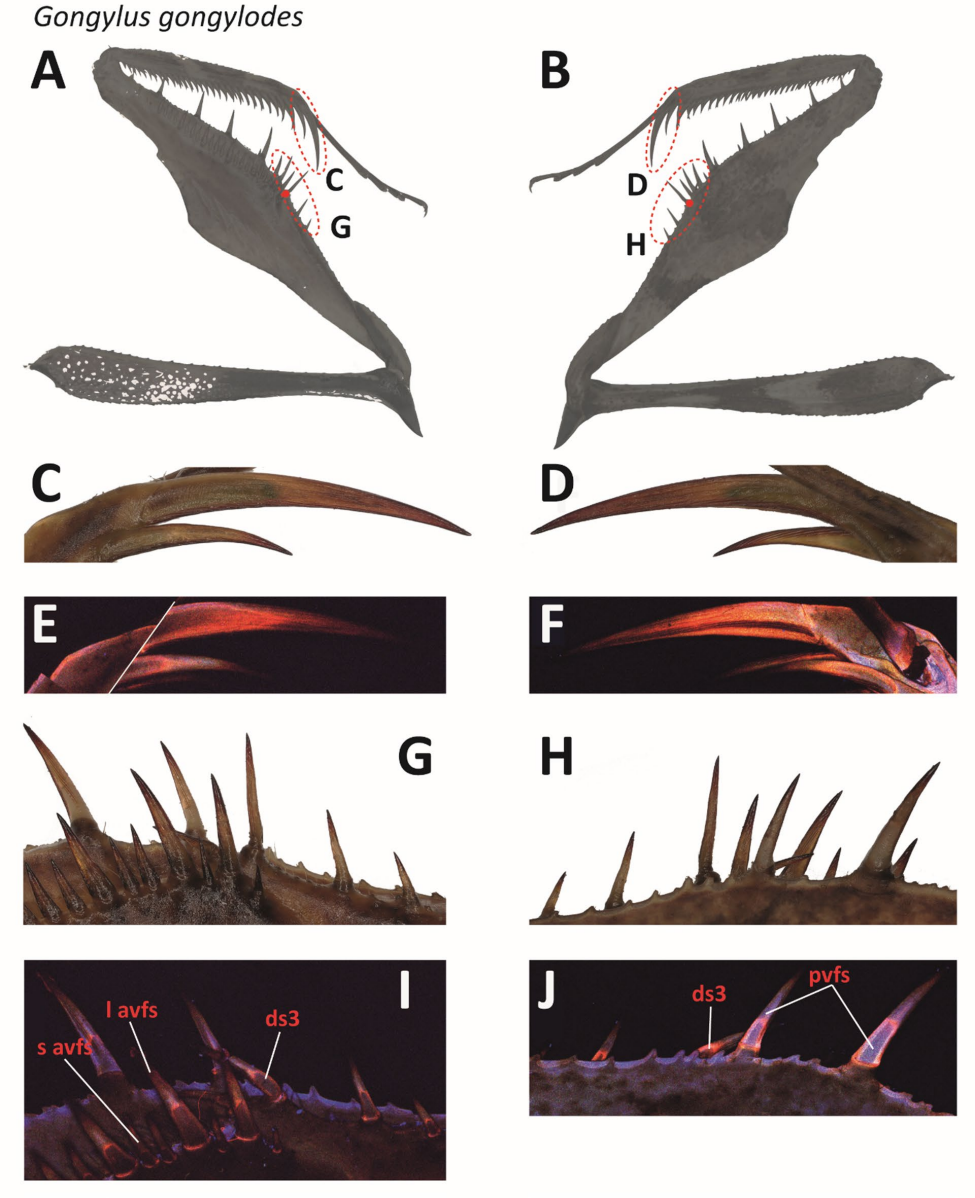
Light microscopy and CLSM images of different spines of *Gongylus gongylodes*, specimen 15, male, left foreleg. A, C, G, I. Medial view. B, D, H, J. Lateral view. The tiltable spine is high lightened by a red dot. C–F. Ts with (non-tiltable) avts. G, I. (non-tiltable) l avfs and s avfs as well as the only tiltable spine (ds3). H, J. In the front: (non-tiltable) pvfs. In the back: the only tiltable spine (ds3). Abbreviations: avts, anteroventral tibial spines; ds3, 3rd discoidal spine; l avfs, long anteroventral femoral spines; pvfs, posteroventral femoral spines; s avfs, short anteroventral femoral spines; ts, tibial spur.

Light microscopy images of the spines are presented in Fig. 9A–D G–H. Under CLSM, the spines appeared either brown (the lower parts of ds, long avfs, and short avfs) or blue (pvfs and avts) (Fig. 9I–J). Towards the spine bases, a red signal was documented. Above the brown region, a red autofluorescence, which faded into a blue, then brown and finally, at the tip, into a red signal, was determined (Fig. 9I–J). The spine sockets appear darker than the spines themselves (Fig. 9I–J). The region of the sockets interacting with the spines showed a red autofluorescence. A blue autofluorescence was determined between the sockets (Fig. 9I–J). The ts exhibited a red autofluorescence that darkened toward the tip (Fig. 9E–F).

#### Intensity of the autofluorescence signals

In *S. lineola*, the autofluorescence was dominated by T1 blue (1.00), followed by T4 red 50% (0.87–0.31), T2 green (0.43–0.20), and T3 red 50% (0.49–0.16).

In *G. gongylodes*, the lateral and medial sides of the forelegs exhibited a different pattern. The medial side showed higher autofluorescence from the T4 red 50% channel as from the T1 blue channel (1.32–1.30). The lateral sides of the forelegs in *G. gongylodes* displayed a pattern similar to that of *S. lineola*, where the T4 red 50% autofluorescence (0.96–0.54) was nearly as strong as the T1 blue autofluorescence (1.00). The autofluorescence signal from the T2 green (0.49–0.28) and T3 red 50% (0.43–0.22) channel varied little and remained up to 60% lower than T1 blue.

### Microstructures

**Fig. 10:**
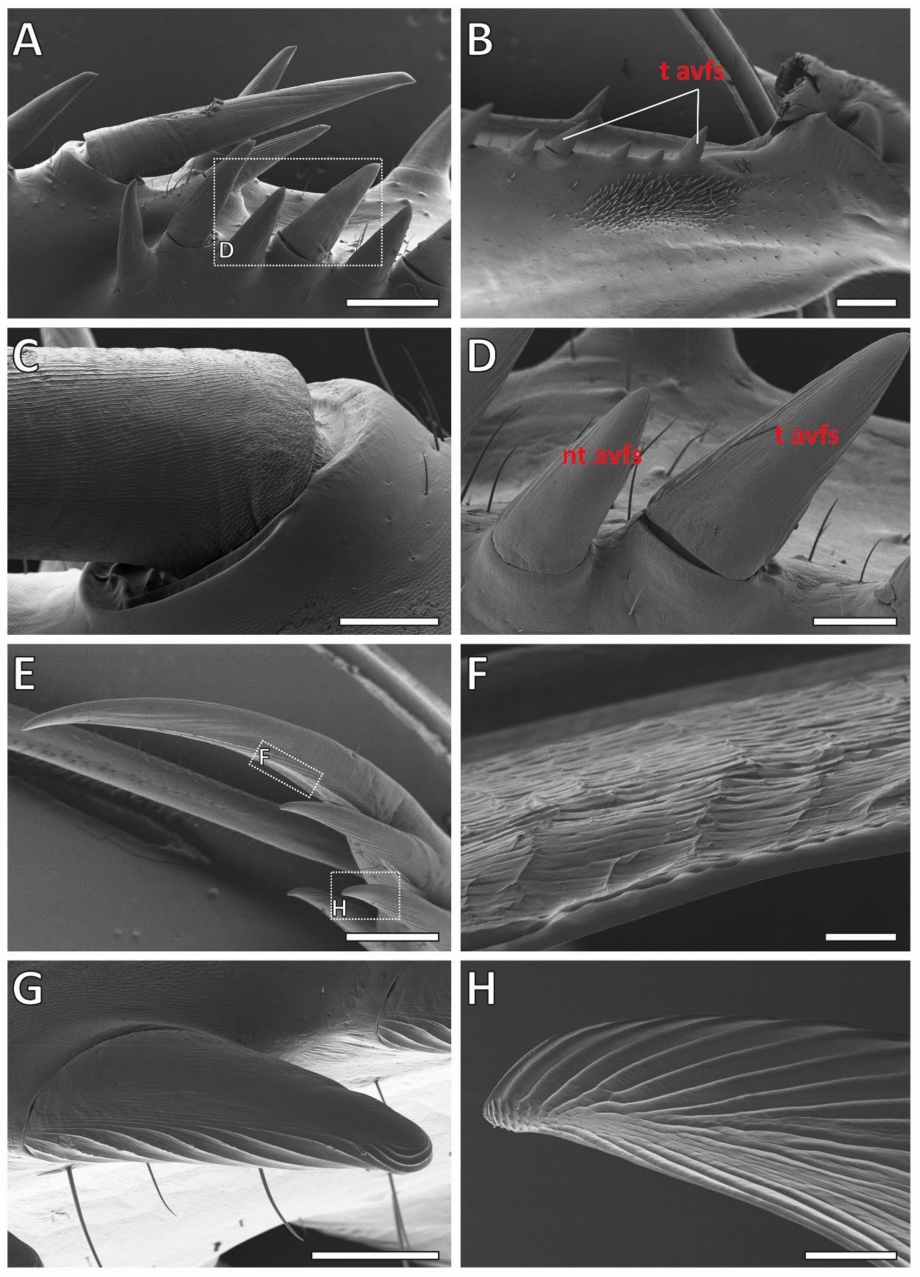
Scanning electron microscopic images of the raptorial apparatus of *Sphodromantis lineola*. (A) Overview on the medial side of the femur. (B) T avfs and nt avfs, located at the femoral brush. (C) (Tiltable) ds3, lateral sides feature fine grooves resembling a honeycomb structure. (D) Nt avfs and t avfs. (E) Ts. (F) Medial side of the ts exhibiting a very rough surface structure. G–H. Tip of the avfs with folds running from right to left, forming a rough honeycomb-like structure beneath the tip on the medial side. Abbreviations: ds3, 3rd discoidal spine; nt avfs, non-tiltable anteroventral femoral spines; t avfs, tiltable anteroventral femoral spines; ts, tibial spur. Scale bars: A, B, E, 800 μm; C, D, G, 200 μm; F, 20 μm; H, 80 μm.

In the SEM images of *S. lineola* (Fig. 10), the joint-carrying sockets of the tiltable avfs (Fig. 10D) and ds3 (Fig. 10C) were documented. In the CLSM images, the joints exhibited a red autofluorescence signal (Fig. 7I–J). The medial sides of the spines exhibited a strong red autofluorescence signal, while the lateral sides, which could tilt, exhibited a strong blue autofluorescence signal (Fig. 7I–J). The most distal avfs, located at the femoral brush, was non-tiltable due to the absence of a joint (Fig. 10B).

**Fig. 11:**
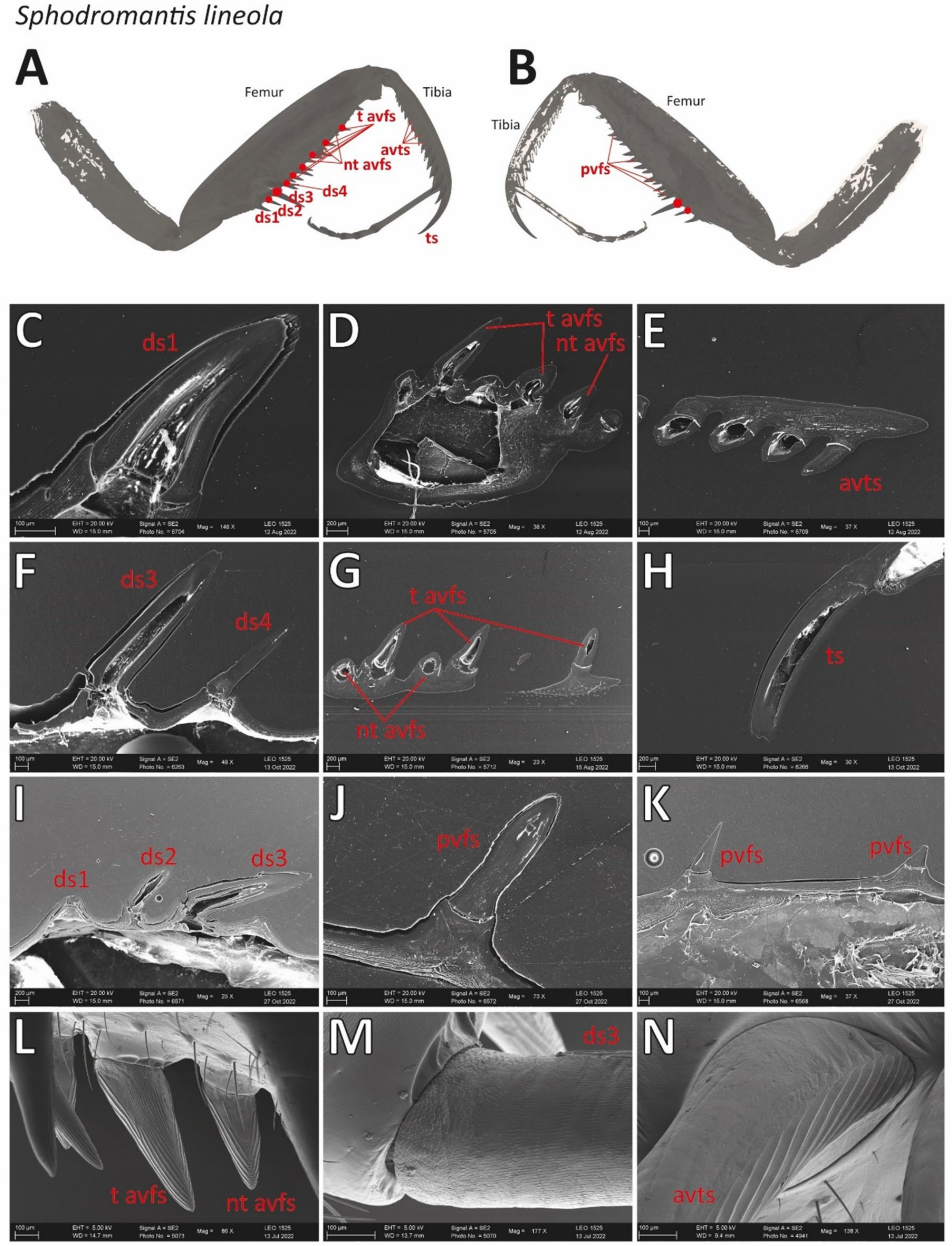
A–B. Overview of the raptorial apparatus of *Sphodromantis lineola* with labelled spine types and highlighted tiltable spines (red dots). (A) Medial view. (B) Lateral view. C–N. Scanning electron microscope images with details of the spines, their bases and sockets. (C) Smoothened ds1. (D) Smoothened nt avfs and t avfs. (E) Smoothened avts. (F) Smoothened ds3 and ds4. (G) Smoothened nt avfs and t avfs with femoral brush. (H) Smoothened ts. (I) Smoothened ds1, ds2, ds3. J–K. Smoothened pvfs. L. Detail medial side of the nt avfs and t avfs. M. Detail base of ds3, medial view. N. Detail base and connection to the tibia of the avts, lateral view. Abbreviations: avts, anteroventral tibial spines; ds1, 1th discoidal spine; ds2, 2nd discoidal spine; ds3, 3rd discoidal spine; ds4, 4th discoidal spine; nt avfs, non-tiltable anteroventral femoral spines; pvfs, posteroventral femoral spines; t avfs, tiltable anteroventral femoral spines; ts, tibial spur.

The polished and smoothened spines exhibited varying socket structures (Fig. 11). In the non-tiltable spines, ring-shaped sockets were documented (Fig. 11C, E, H, J, K). The bases of the tiltable ds2 and ds3 possessed a constriction on their proximal side, while the sockets were elevated (Fig. 11F, I). The tiltable avfs (Fig. 11D) showed a smaller constriction and their sockets were less differentiated (Fig. 11G) compared to those of the tiltable ds.

The lateral sides of the spines featured fine grooves resembling a honeycomb structure (Fig. 10C), whereas the medial sides displayed large folds running toward the tips of the spines (Fig. 10A, D). At the tips of the pvfs, the non-tiltable avfs, the tiltable avfs, and the avts, the folds run from right to left, forming a rough honeycomb-like structure beneath the tip on the medial side (Figs. 10H and 11L). In contrast, the tips of the ts and ds were smooth, while the medial side of the ts revealed a very rough surface structure that exhibited a dark red autofluorescence in the CLSM (Figs. 7E and 10F).

**Fig. 12:**
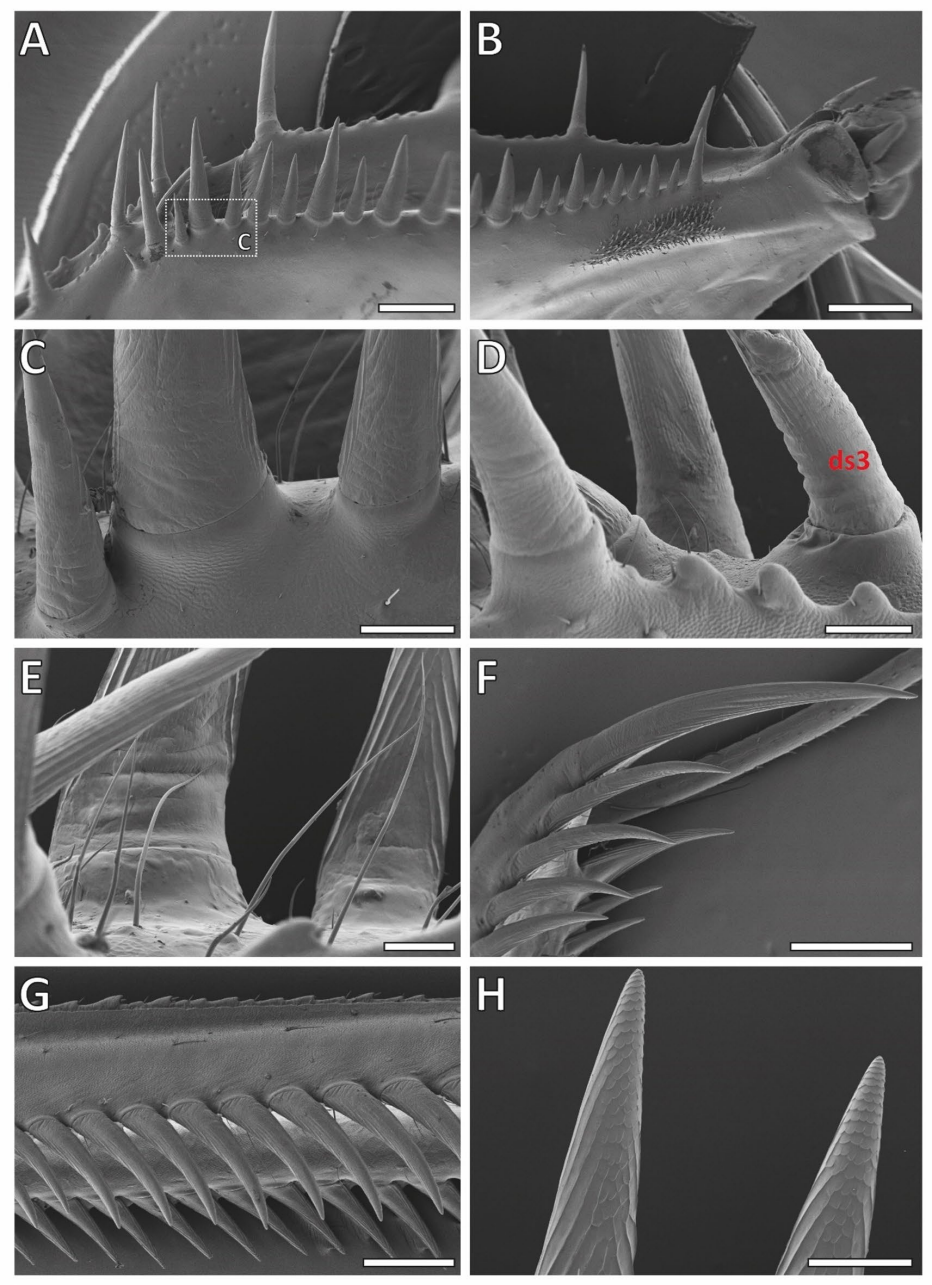
Scanning electron microscopic images of *Gongylus gongylodes*. (A) Overview on the medial side of the femur. (B) Femoral brush with (non-tiltable) spines. (C) Medial view on (non-tiltable) spines, their bases and sockets. (D) Right side: (tiltable) ds3. (E) The lateral sides of the spines featured vertical lines whereas the tips were smooth. (F) Ts. (G) Avts in lateral view. (H) Vertical folds on the medial side, running toward the tips and resembling upright scales arranged in rows. Abbreviations: avts, anteroventral tibial spines; ds3, 3rd discoidal spine; ts, tibial spur. Scale bars: A, B, 1000 μm; C, D, 200 μm; E, H, 100 μm; F, 800 μm; G, 500 μm.

**Fig. 13:**
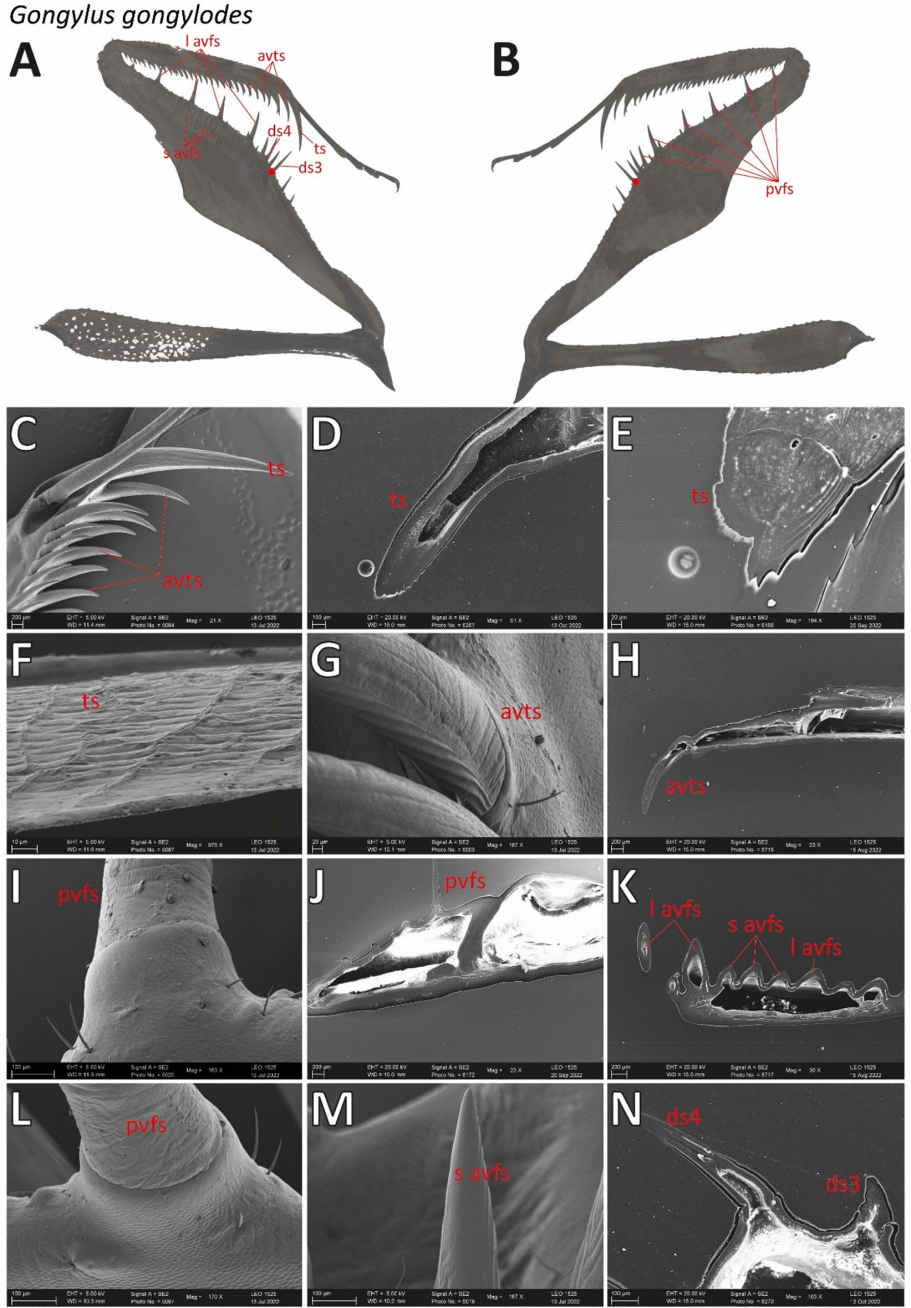
A–B. Overview of the raptorial apparatus of *Gongylus gongylodes* with labelled spine types. The tiltable ds3 is high lightened by a red dot. C–N. Scanning electron microscope images with details of the spines, their bases and sockets. C. Tip of the tibia with ts and avts, lateral view. D. Smoothened ts. E. Detail of the smoothened base of the ts. F. Detail of the contact surface of the ts, lateral view. G. Detail of the base of avts, medial view. H. Detail of the smoothened avts. I. Detail of the base of pvfs, lateral view. J. Smoothened pvfs. K. Smoothened l avfs and s avfs. L. Detail of the base of pvfs, medial view. M. Detail of the lateral side of the tip of s avfs. N. Smoothened ds3 and ds4. Ds 3 is tiltable. Abbreviations: avts, anteroventral tibial spines; ds3, 3rd discoidal spine; ds4, 4th discoidal spine; l avfs, long anteroventral femoral spines; pvfs, posteroventral femoral spines; s avfs, short anteroventral femoral spines; ts, tibial spur.

For detailed images of the spines of *G. gongylodes*, please refer to Figs. 12 and 13. In contrast to *S. lineola*, the forelegs of *G. gongylodes* featured only one tiltable spine, the ds3 (Fig. 12D). In the CLSM, this spine showed no blue autofluorescence at the base-socket joint (Fig. 9I), different to the tiltable spines in *S. lineola*. The socket shape (Fig. 13N) was, however, similar to the sockets of the tiltable spines in *S. lineola*. Most non-tiltable spines possessed a circular base, similar to those in *S. lineola*. The fourth ds showed a different base shape (Fig. 13N) with a constriction just above the base, which seems to keep the spine in a defined angle towards the femur.

Similar to the spines of *S. lineola*, those of *G. gongylodes* displayed vertical folds on the medial side, running toward the tips. However, different to *S. lineola*, they did not form a rough honeycomb structure at the tip. Instead, their shape resembled upright scale-like structures arranged in rows (Fig. 12H). In the CLSM, the vertical folds exhibited strong dark red autofluorescence, while the adjacent areas displayed blue to green autofluorescence (Fig. 9I). The lateral sides of the spines featured delicate vertical lines, which could not be determined in *S. lineola*, whereas the tips were smooth (Fig. 12E). Similar to *S. lineola*, the tibial spur displayed a broad, long, rough vertical line on its medial side (Fig. 12F), which exhibited a strong dark red autofluorescence (Fig. 9F).

### Elemental composition

The forelegs consisted to 98.4 ± 0.5 atomic % (mean ± standard deviation) of the elements H, C, N, and O. The remaining 1.6 ± 0.5 atomic %, referred to as trace elements (Te), consisted of Ca, Na, Mg, Si, P + Pt, S, Cl, K, F, Cu, Mn, Zn, and Fe. The most common trace element, with an atomic proportion of 0.91 ± 0.3 atomic %, was F, followed by P + Pt (0.23 ± 0.13), and Mg (0.07 ± 0.05). Cu, Cl, and Zn followed with 0.05 ± 0.05 atomic %, while Ca, S, Fe, and K were present with 0.03 ± 0.03 atomic %. Si was determined with 0.02 ± 0.07 atomic %, Mn with 0.02 ± 0.01 atomic %, and Na with 0.00 ± 0.01 atomic % (for ranking, see Supplementary Table 2).

**Fig. 14:**
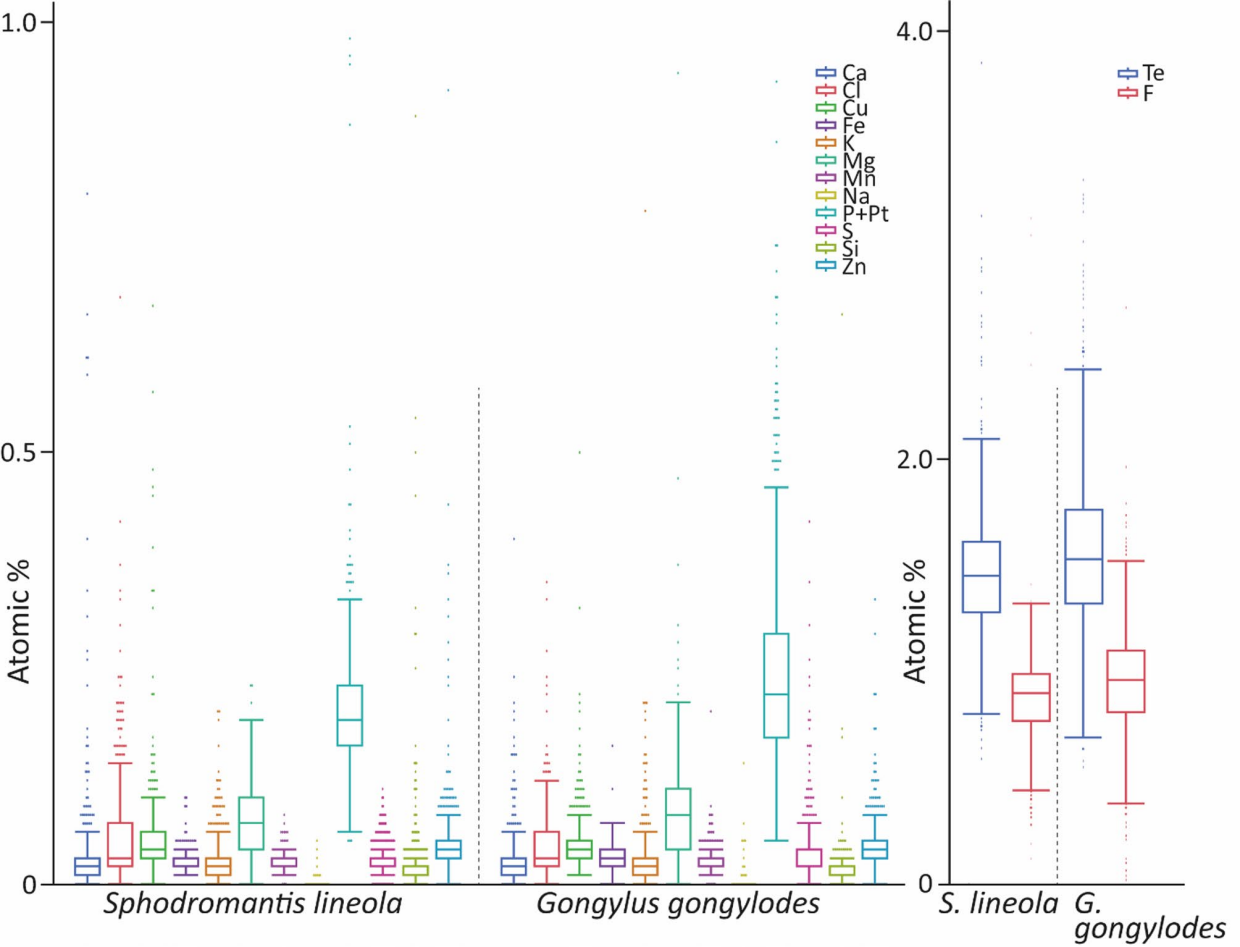
Results from EDX analyses, given in atomic %, for each species. For statistical comparison, see Supplementary Table 3. Abbreviations: Te, trace elements, sum of Ca, Cl, Cu, F, Fe, K, Mg, Mn, Na, P + Pt, S, Si, and Zn.

When examining the species individually (Fig. 14), the order of the first seven trace elements did not change, and Na remained in last place (for ranking see Supplementary Table 2). Following Zn, *G. gongylodes* contained S, Fe, K, Ca, Mn, and Si, while in *S. lineola*, K, Si, Fe, S, and Mn followed (for ranking, see Supplementary Table 2). Furthermore, there were significant differences between the species in the proportions of trace elements (for values and for p-values, see Supplementary Table 3). The content of F, Fe, K, Mg, Mn, P + Pt, and S were significantly higher in *G. gongylodes* than in *S. lineola*. Conversely, the concentration of Si was significantly higher in *S. lineola* than in *G. gongylodes*. There were no significant differences in the proportions of Ca, Cl, Cu, Na, and Zn (for values and p-values, see Supplementary Table 3).

**Fig. 15:**
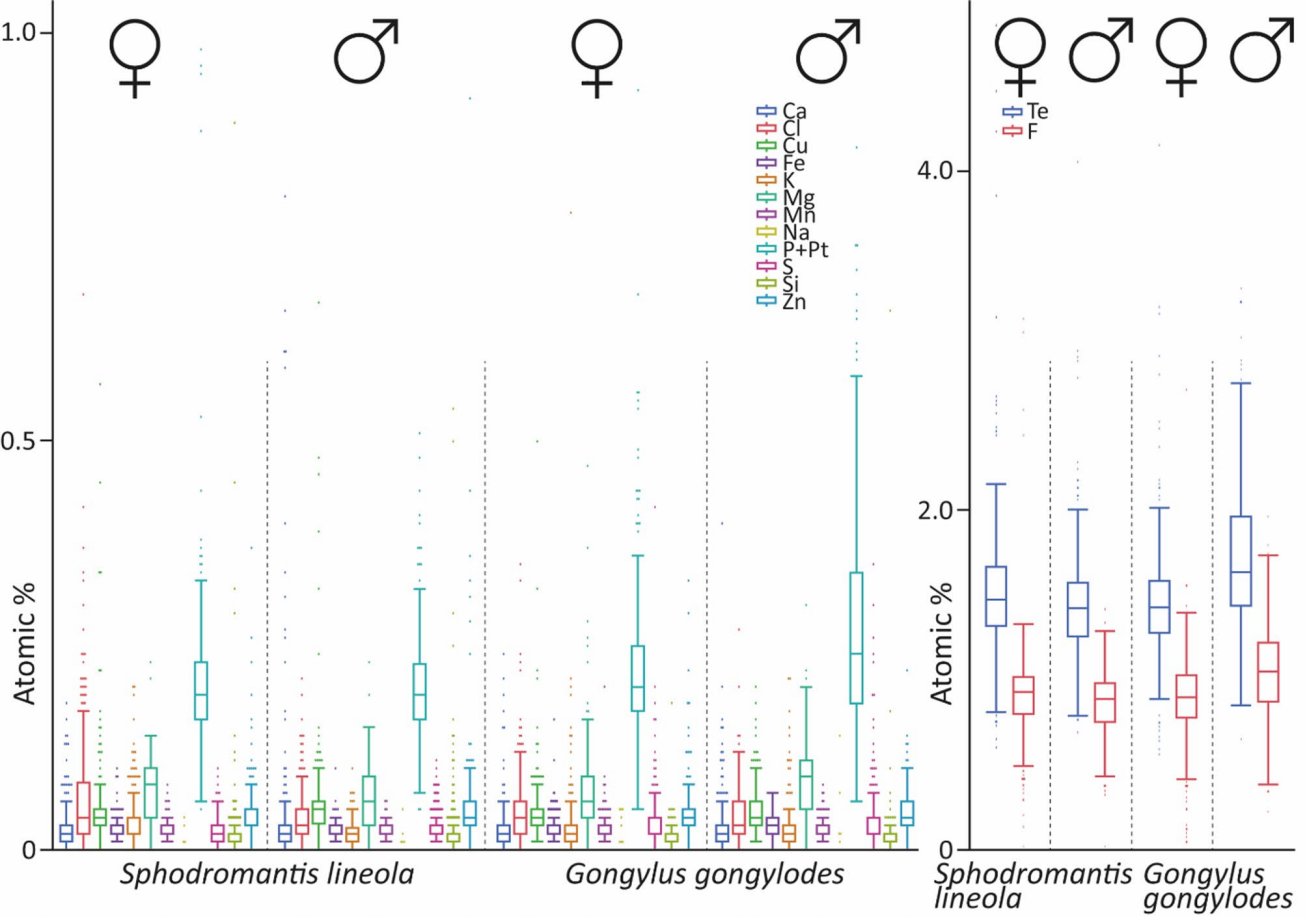
Results from EDX analyses, given in atomic %, for each species and sex. For values see Supplementary Tables 4 and for statistical comparison see Supplementary Tables 5 and 6. Abbreviations: Te, trace elements, sum of Ca, Cl, Cu, F, Fe, K, Mg, Mn, Na, P + Pt, S, Si, and Zn.

In different sexes within each species (Fig. 15; for values, see Supplementary Table 4), the order of trace elements (based on their atomic %) did not seem to follow a strict pattern (for ranking see Supplementary Table 2).

In *S. lineola*, the female possessed significantly higher proportions of Cl, F, K and Mg, than the male, while the male exhibited significantly higher proportions of Cu, S, and Zn than the female (for p-values, see Supplementary Table 5). The proportions of P + Pt, Si, Fe, Ca, or Mn did not differ between the sexes.

In female *G. gongylodes*, F was followed by P + Pt, Mg, Cl, Cu, Zn, S, Fe, K, Mn, Ca, Si, and Na (for ranking see Supplementary Table 2). In the male, Cl was not in fourth place, but instead followed Cu and Zn in sixth place, and the elements K and Ca were switched. Additionally, there were differences between the sexes in the atomic proportions of the trace elements (for p-values, see Supplementary Table 6). The male exhibited significantly higher proportions of Cu, F, Fe, P + Pt, Mg, Si, and Zn, while the female showed a significantly higher atomic proportion of Cl. The trace elements Ca, K, Mn, Na, and S, however, did not differ.

The following section focuses on the significant differences in the elemental composition within the forelegs of each examined specimen (for *S. lineola* see Supplementary Fig. 9 and Supplementary Fig. 10; for *G. gongylodes* see Supplementary Fig. 11 and Supplementary Fig. 12; for values, see Supplementary Table 7). Only areas that significantly differ in their elemental composition from several other regions are highlighted to underscore recurring patterns.

In the male *S. lineola*, the medial side of the tibia (region 3) showed a higher Ca and Cl content, as well as a lower Mg content than the other regions. The cuticle between the avts and pvts (region 1) stood out due to a higher Cu and Zn content. The dorsal cuticle of the medial side of the femur (region 2) exhibited a higher (10 to 15 times) Ca content than the other regions of the femur. Additionally, this region possessed the lowest Cl and the highest Cu content. Moreover, the S content increased from region 5 towards the dorsal side of the medial side of the femur (region 2). The non-tiltable avfs possessed the highest Cl content. There were no further differences between the spines (Supplementary Fig. 9; for values see Supplementary Table 7).

In the female *S. lineola*, the Ca content of the lateral side of the tibia (region 3) was four times higher than in the other regions (region 1 and 2), while the medial cuticle (region 2) possessed a lower Cl content. The (non-tiltable) ds4 exhibited a higher Ca and the highest K content, while the large (tiltable) ds3 possessed the lowest K content compared to the other spines. The non-tiltable avfs showed the highest Cl content (Supplementary Fig. 10; for values see Supplementary Table 7).

There were three similarities in the elemental composition between male and female *S. lineola*. The lateral sides of the tibiae showed a higher Ca content compared to the medial sides of the tibiae. On the femur, the lateral side (region 2) exhibited the lowest Cl content, while the non-tiltable avfs possessed the highest Cl content among the spines.

In the female *G. gongylodes*, the defined regions of the tibia differed in their elemental composition primarily with regard to Cl. The dorsal cuticle of the medial side of the femur (region 3) possessed a higher Cl content compared to the middle cuticle of the medial side of the femur (region 4). The cuticle between the avts and pvts (region 1) contained also more Cl. Along the femur, the dorsal femoral process (region 3) differed from the regions 4 and 5, showing a lower S and Cu content. The lateral side of the femur (region 1), in contrast, possessed a lower Cl content than the region along the spines (region 5) and the femoral process (region 3). The only tiltable ds (ds3) stood out in its elemental composition. It exhibited a K content five times higher than the other spines, and its Cu content was also elevated. The ds4 possessed a lower Mg content compared to the pvfs, the long avfs, the short avfs, and the avts as well as the ts. Additionally, its F content was lower than that of the avts and the long avfs (Supplementary Fig. 12; for values, see Supplementary Table 7).

The male *G. gongylodes* exhibited a similar pattern. The lateral side of the tibia (region 3) possessed a higher Cl content, while the medial side (region 2) showed a higher Cu content and a lower Mg content. The area between the avts and pvts (region 1) stood out by a high F content, whereas other elements (Ca, Fe, K, Mn, P + Pt) were present in lower proportions than in other regions of the tibia (regions 2 and 3). The region 5, beneath the long and short avfs, showed an elevated Ca and P + Pt content. The cuticle (region 4) between the ventral cuticle and the femoral process possessed a higher Mg content. Both areas (region 4 and 5) exhibited a higher F content than the lateral side of the femur (region 1) and the femoral process (region 3). The ds3 and ds4 showed lower F and Mg content than the other spines. Additionally, the ds3 and ds4 as well as the avts possessed twice as much Cl in comparison to the other spines of the foreleg (Supplementary Fig. 11; for values, see Supplementary Table 7).

There were three similarities in the elemental composition between male and female *G. gongylodes*. The lateral side of the tibia (region 3) possessed a higher Cl content compared to the medial side of the tibia (region 2) and the spines. Furthermore, the ds4, which is bent, in both males and females possessed less Mg and F than the other spines.

The only similarity between both species was the increased Cl content on the lateral side of the tibia (regions 3).

### Gradients along the tibial spur

**Fig. 16:**
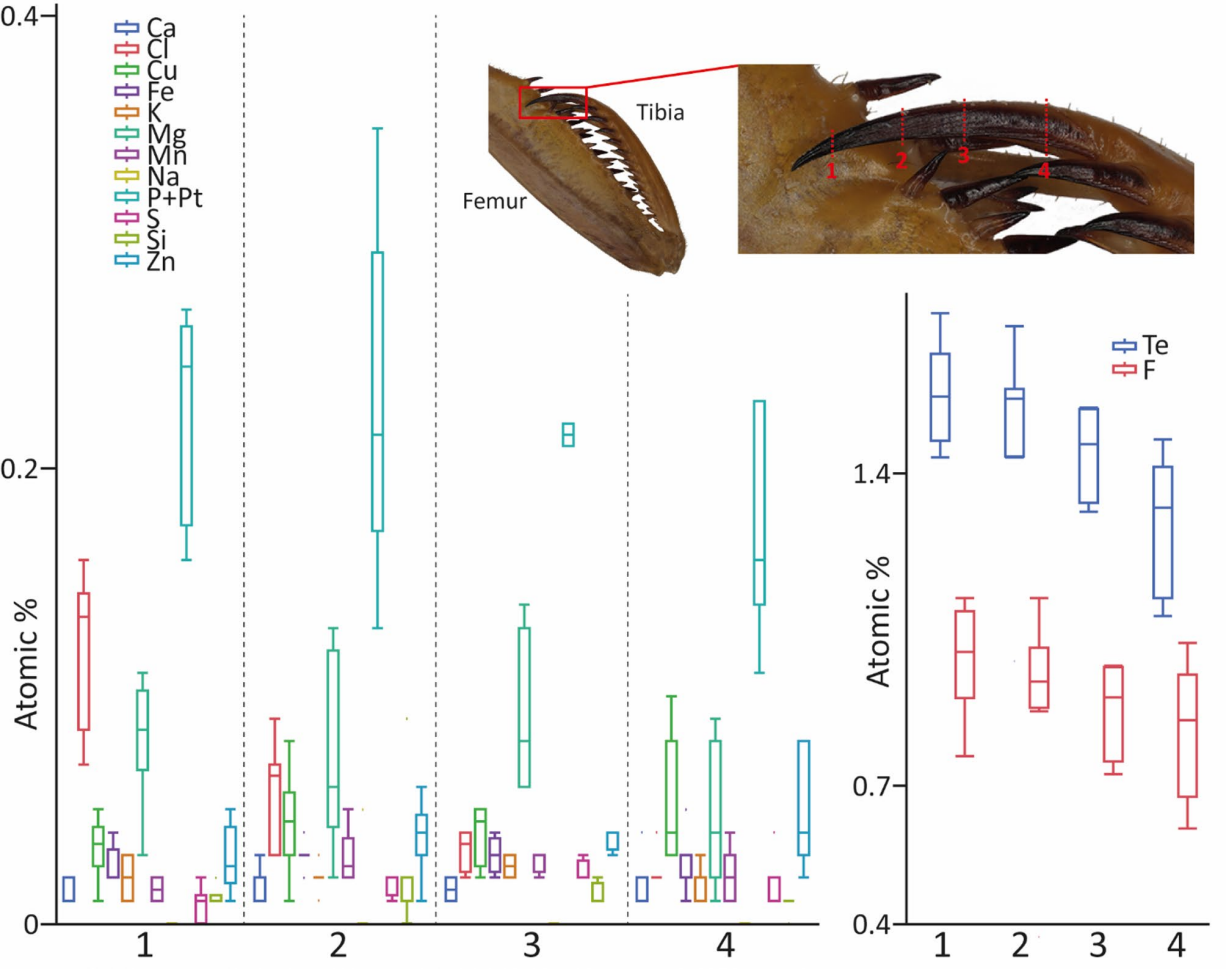
Results from EDX analyses, given in atomic %, for the tibial spur of *Sphodromantis lineola*, male, right foreleg, specimen 01 – sorted to the tested regions of the tibial spur. For values see Supplementary Table 8. Medial view on the foreleg. Abbreviations: Te, trace elements, sum of Ca, Cl, Cu, F, Fe, K, Mg, Mn, Na, P + Pt, S, Si, and Zn.

In addition to summarizing measurements to specific regions, we tested for gradients within the ts of the male *S. lineola* (Fig. 16; for values, see Supplementary Table 8). We determined an increase in the trace elements (Te) from the base (1.30 atomic %) to the tip of the spur (1.57 atomic %). A large portion of this was due to the F content, which increased by 0.16 atomic %, followed by Cl (0.1 atomic %), Mg (0.04 atomic %), and P + Pt (0.04 atomic %). Cu, Zn, S, and Ca showed a slight decrease (by approximately 0.01–0.02 atomic %).

### Mechanical properties

No differences between males and females in mechanical properties were observed within each species.

**Fig. 17:**
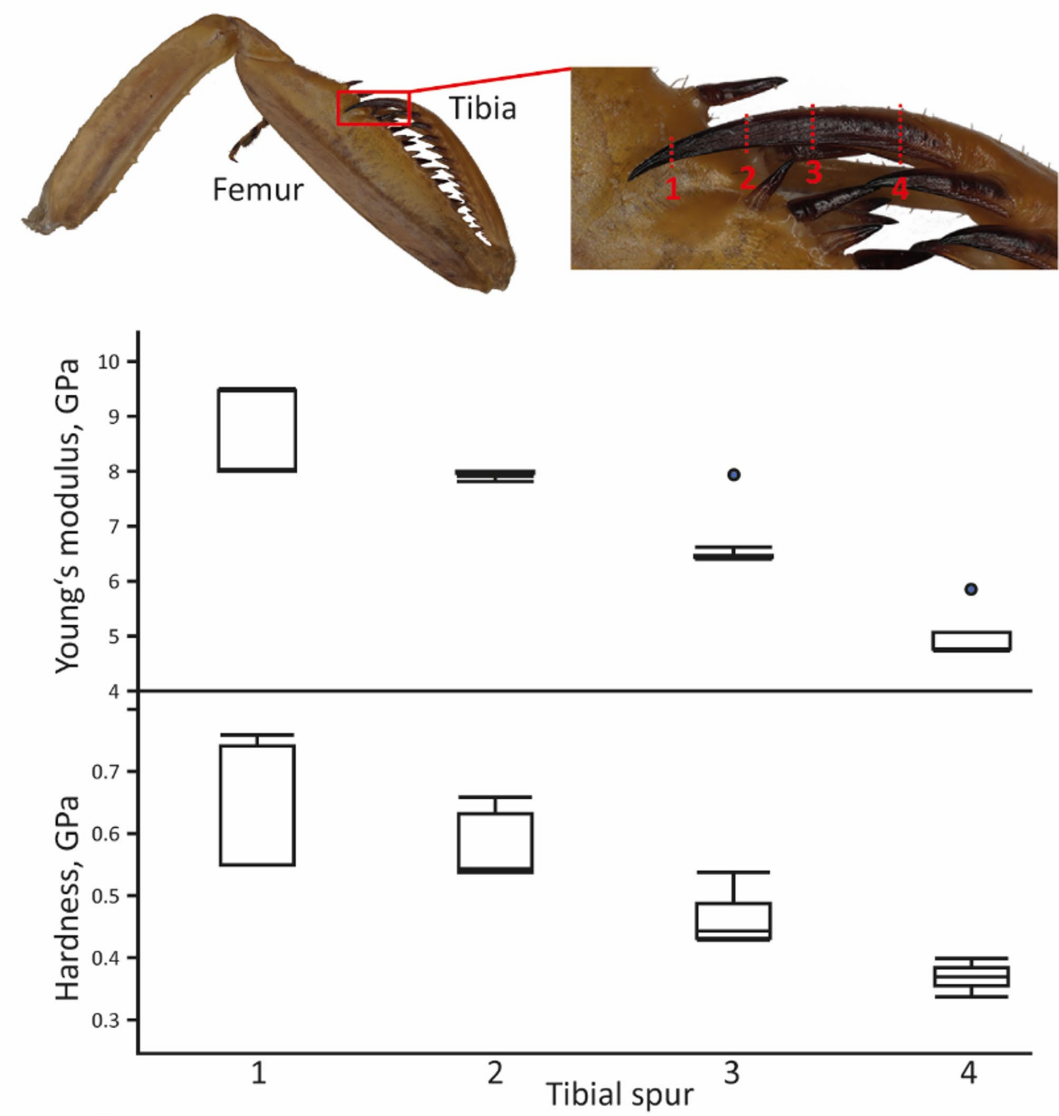
Results from nanoindentation experiment, hardness and Young’s modulus, both given in GPa, for the different regions of the tibial spur of *Sphodromantis lineola*. For values, see Supplementary Table 9.

The ts tip (region 1) was the hardest (Fig. 17; for vales of hardness, see Supplementary Table 9) and stiffest region of the foreleg in *S. lineola* (mean ± standard deviation of the Young’s modulus: 8.64 ± 0.78 GPa). Within the ts, we detected a significant (for p-values, see Supplementary Table 10) decrease in both hardness and Young’s modulus: from region 2 (7.97 ± 0.02 GPa), across region 3 (6.64 ± 0.53 GPa), and finally region 4 as the softest and most flexible region (4.96 ± 0.44).

**Fig. 18:**
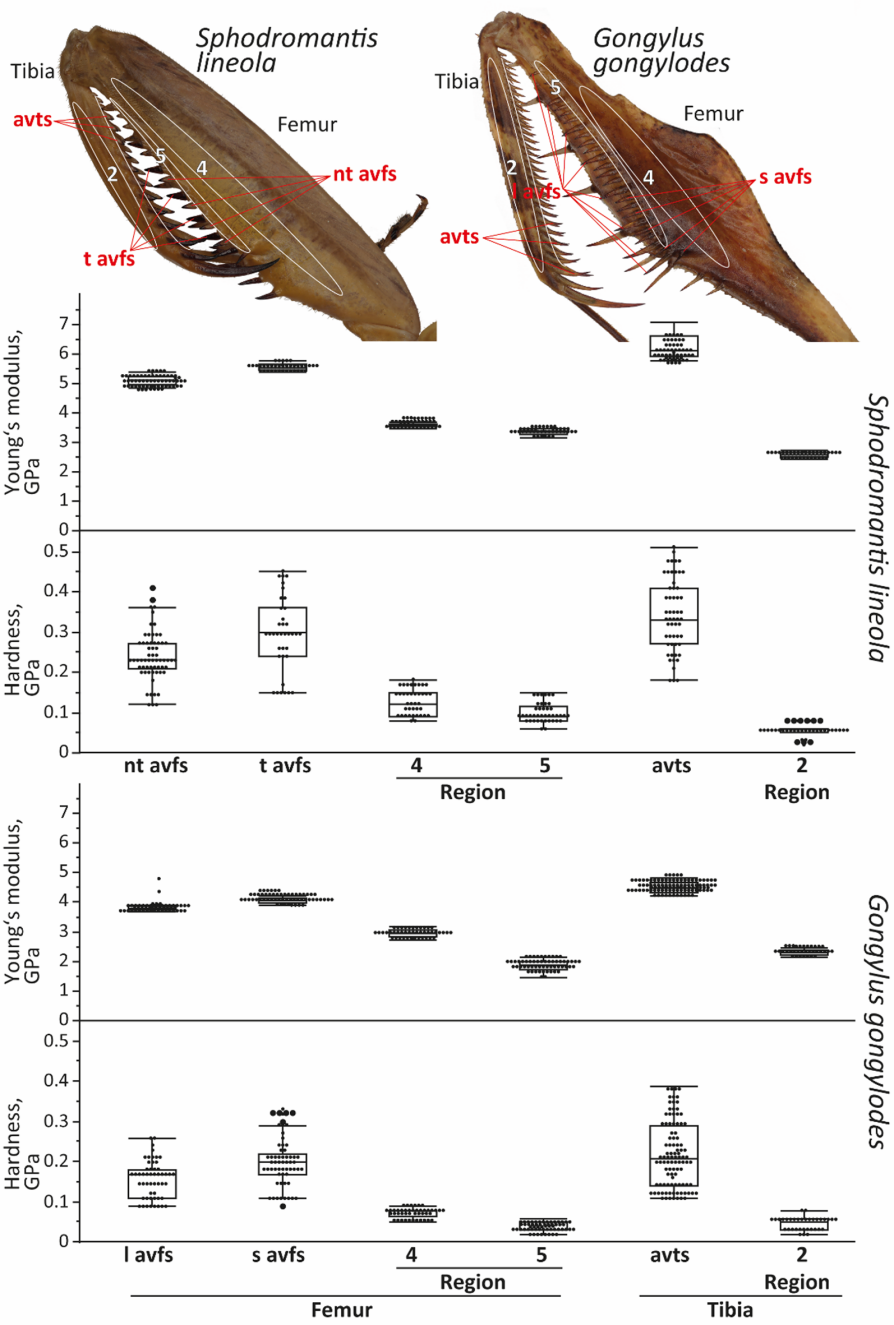
Results from nanoindentation experiment, hardness and Young’s modulus, both given in GPa, for different regions and spines of *Sphodromantis lineola* and *Gongylus gongylodes*. For values, see Supplementary Table 11. Abbreviations: avts, anteroventral tibial spines; l avfs, long anteroventral femoral spines; nt avfs, non-tiltable anteroventral femoral spines; s avfs, short anteroventral femoral spines; t avfs, tiltable anteroventral femoral spines.

The spines were the hardest (Fig. 18; for vales of hardness, see Supplementary Table 11) and stiffest parts of the other tested regions; spines of *S. lineola* were significantly harder and stiffer than the spines of *G. gongylodes* (for p-values, see Supplementary Tables 12 and 13). Hardest and stiffest part were the avts of *S. lineola* (6.27 ± 0.40 GPa), followed by the tiltable avfs of *S. lineola* (5.56 ± 0.11 GPa), the non-tiltable avfs of *S. lineola* (5.11 ± 0.15 GPa), the avts of *G. gongylodes* (4.50 ± 0.19 GPa), the short avfs of *G. gongylodes* (4.05 ± 0.08 GPa), and finally the long avfs of *G. gongylodes* (3.79 ± 0.07 GPa).

Regions of *S. lineola* were significantly harder (Fig. 18; for vales of hardness, see Supplementary Table 11; for p-values, see Supplementary Tables 12 and 13) and stiffer than the regions of *G. gongylodes*. The femoral region 4 of *S. lineola* was the hardest and stiffest (3.57 ± 0.06 GPa) one, followed by the femoral region 5 of *S. lineola* (3.33 ± 0.09 GPa), the femoral region 4 of *G. gongylodes* (2.96 ± 0.13 GPa), the tibial region 2 of *S. lineola* (2.58 ± 0.09 GPa), the tibial region 2 of *G. gongylodes* (2.29 ± 0.08 GPa), and the femoral region 5 of *G. gongylodes* (1.86 ± 0.17 GPa) was the softest and most flexible one.

### Relationship between mechanical parameters and elemental composition

Hardness and Young’s modulus showed a very strong relationship (*r* = 0.88). Both Young’s modulus and hardness did not correlate with any of the discussed elements (for correlation coefficients, see Supplementary Table 14; for relationships between elements and mechanical properties, see Supplementary Fig. 13).

## Discussion

### Morphology

Todays and fossil Mantodea are pursuit and ambush predators. This feeding strategy has evolved convergently to different insect groups^2,65^.

According to^49^, many species of Mantodea exhibit a similar morphology of the raptorial appendages, similar to the forelegs of the generalist feeder *Hierodula membranacea*. These generalist raptorial forelegs are thought to provide a selective advantage, allowing mantises to successfully capture various types of prey. A different morphology of the raptorial apparatus is only seen, according to^49^, when mantises (a) specialize in a particular type of prey or (b) live in a habitat where only a specific type of prey exists (for example, highly sclerotized Tenebrionidae beetles, which are adapted to desert climates).

In the species studied here, the raptorial forelegs were highly different in their morphologies, specifically *Sphodromantis lineola* forelegs were much broader and thicker than the forelegs of *Gongylus gongylodes*. Potentially, the foreleg morphology is adapted to reducing the drag forces, similar to the situation in stomatopod raptorial forelegs^66^. *G. gongylodes* forelegs potentially experience less drag force during prey capture due to their thinness. This, however, could potentially increase the risk of structural failure, when the tibia is bended, which is potentially decreased by the presence of the dorsal femoral inflation in the forelegs. *S. lineola* forelegs are additionally composed of a thicker cuticle, which probably reduces structural failure, when experiencing higher stresses. In the mandibles of these species, we previously also documented a similar pattern, as the cutting edge is composed of thicker cuticle in comparison to the rest of the mandible aiding in reducing fracture and minimizing wear^19^. The thinness of *G. gongylodes* forelegs could, however, together with their colour, also relate to mimicry, as this is the case for many mantis species^67^.

Variations in spine morphology between the sexes, such as seen in *Eremiaphila denticollis*, are thought to be an expression of pronounced sexual dimorphism^49^. In our specimens, we could however, not detect differences between females and males in the number of spines.

While the forelegs of *S. lineola* resemble those of the generalist *H. membranacea*, *G. gongylodes* possesses a higher amount of long, delicate spines to fixate their prey [see^48^]. Potentially, similar to the situation in adult dragonflies^68^, the long spines of *G. gongylodes* serve as the walls of a catch basket, formed by the profile of the femur and the inclination of the spines.

The long, delicate spines also suggest the capture of smaller insects, as these spines are more prone to breaking or wearing down when dealing with tougher, more sclerotized prey^48,49,55^. Additionally, *G. gongylodes* and *S. lineola* differed in the number of movable spines. *S. lineola* possesses two movable discoidal spines and six movable anteroventral spines, whereas *G. gongylodes* has only one movable discoidal spine. However, ^48^ noted that *G. gongylodes* has several movable spines, including three discoidal spines and larger spines along the front edge (without further specification) that are movable. When the forelegs of dead mantises dry out, the spines lose their mobility usually within a day. However, rehydrating them with water partially restored their mobility, and spines preserved in alcohol retain their ability to tilt^49^. Although our study of tiltable spines was conducted on the fresh material, the similarity in the socket structure of the third and fourth discoidal spines in *G. gongylodes* suggests that at least one additional spine might be movable. A future study of the spines and socket structures on fresh foreleg material could provide further clarity in this matter.

Overall, the function of the tiltable spines in mantises is poorly understood. In general, the tiltable spines do not usually interact with the tibial spines when the foreleg closes. They are thought that they cannot be actively tilted and may function as mechanoreceptors^69^. The so-called prothoracic-tibial flexion reflex PTFR^70^ enables the mantis to open its forelegs following a failed capture attempt, allowing it to strike again within approximately 100 ms. When prey capture is successful, the tilting of the movable spines suppresses the PTFR^69^.

The elongated discoidal spine is also thought to help to narrow the escape angle of the prey from the raptorial apparatus, working together with the tibial spur to roll the prey further into the trap^49^. This function is probably also enhanced by the morphology of the femoral spines, which, especially in *S. lineola*, are bend towards the femur-tibial joint and thus likely decrease the ability of the prey to escape from the foreleg. The morphologies of the joints between the bases and the sockets of the tiltable spines seem to fulfil this function as well. The sockets are elongated towards proximal (Fig. 11F, I), which probably reduce the spines’ tiltability towards proximal and thus work as supporting structure. Additionally, the microstructure of the spines might contribute to reducing the ability of the prey to escape, as the medial spine surfaces are rougher and probably increase friction and/or mechanical interlocking (Figs. 10 and 12)^71^.

Another study suggests that the tiltability of the spines does not increase capture success, but contributes to better handling of prey during feeding^50^. According to^72^, movable spines are also found in Blattidae (*Periplaneta americana*)^73^ and could have existed in the common ancestors of Dictyoptera^74^. Some species of mantis shrimp (Stomatopoda) also possess tiltable spines, but these have not been studied in detail with regard to their precise function. Even though fossil Mantodea^75,76^ possess spines, it is not clear why some species (e.g., *G. gongylodes*) possess fewer or no tiltable spines. Potentially, the capability of spines to tilt is necessary to reduce structural failure when hitting harder prey items; this in turn would mean that spines handling softer prey are potentially not as prone to structural failure and thus do not need a capability of bending.

Another possible explanation for the presence of tiltable spines could be the adjustment of the distance between the anteroventral spines on the femur. By altering the spacing between the spines via the tilting mechanism, insects could be better secured, allowing *S. lineola* to capture invertebrates of various sizes by clamping their limbs, thus increasing its range of prey compared to *G. gongylodes*. However, this hypothesis, has not been discussed and therefore awaits deeper investigations in future studies.

### Mechanical properties of the forelegs

The cuticle of insects consists of chitin fibres embedded in a matrix of proteins^42^. The mechanical properties range from KPa to GPa, depending on the water content and the composition [^77,78^; see reviews by^42^ and^79^]. Values of different studies are difficult to compare because of different testing conditions or sample preparation^42,79^. In general, however, arthropod structures that are prone to abrasion, such as mouthparts, joints, or claws, tend to exhibit higher hardness and Young’s modulus values [e.g.,^9,10,13,16,32,36,80–85^], compared to structures, such as wings, elytra, or eyes [e.g.,^45,86–90^].

The nanoindentation results from the femur and tibia of mantids in this study were similar to the mechanical properties determined previously for the forelegs of *Mantis religiosa*^71^. In our specimens, the tibial spur and the spines were the hardest and stiffest regions, likely because they are prone to structural failure due to their thinness and interaction with the prey. In *M. religiosa*, the tibial spur tip was also the stiffest element followed by the basis of the tibial spur^71^. The softer and more flexible basis probably allows a bending, when high forces act onto the structure [see also^71^]. We detected, that the structures of *Gongylus gongylodes* were softer and more flexible than the corresponding regions in *Sphodromantis lineola*. Potentially this could be explained with the different prey, as the forelegs of *S. lineola* potentially experience higher stresses during capturing and holding of the prey. The same pattern has been previously detected in the mandibles, as *S. lineola* possessed harder and stiffer mandible cutting edges as *G. gongylodes*^19^.

However, we here tested only two specimens per species by nanoindentation, which is a small sample size and thus may not represent the entire species. Additionally, these specimens were obtained from private breeders and were not caught in the wild, which could influence the results as it has been previously shown that the environment influences the material of insect cuticle and the morphologies of structures^91–94^. This, however, awaits further investigations with an elevated number of wild-caught specimens.

### The origin of mechanical properties

The mechanical properties of insect cuticle are affected by the extent of sclerotization [e.g.,^41,95^], the specific microstructure of the chitin-protein complex^96–98^, the proportions of mineral components present^40,99–101^, and the distribution of transition or alkaline earth metals [e.g.,^7,13,25^].

^57^ established a protocol to determine regional material properties employing CLSM, which has since been frequently applied to insects. Chitin-containing structures, such as the cuticle of wings^102,103^, thorax^104^, tarsi and legs^105–108^, as well as mouthparts^8–11,109–112^, were on focus in these studies.

These studies have revealed a consistent pattern: autofluorescence signals obtained from laser-excited cuticle, following the method of^57^, were linked to specific material properties. Sclerotized, hard, and stiff cuticle is associated with signals from lasers with wavelengths of 555 nm and 639 nm (appearing red in CLSM images). Weakly sclerotized, flexible, and relatively hard cuticle is linked to signals from a 488 nm wavelength laser (shown as green in CLSM images). Regions rich in resilin, a soft and flexible protein, or other not-tanned proteins, produce signals from a 405 nm wavelength laser (displayed as blue in CLSM images). As^57^ discussed, other proteins can, however, exhibit a similar autofluorescence maximum as resilin^113–116^. Areas with both weak sclerotization and a high proportion of proteins are depicted in shades of yellow, brown, or pink, depending on the proportions of the components.

The CLSM images obtained in this study reveal a significant difference in the autofluorescence of certain areas along the forelegs of both species. The coxa, femur, and tibia of *S. lineola* exhibit markedly higher autofluorescence in the blue to light blue range compared to *G. gongylodes*. This is also evident, when analysing the histograms of the grayscale-separated channels. According to^57^, blue autofluorescence areas are highly resilient, while a combination of green and blue autofluorescence corresponds to a mix of slightly sclerotized areas that also contain resilin. However, when handling the forelegs, those of *G. gongylodes* are noticeably more flexible than those of *S. lineola*. This observation was validated by the results of our nanoindentation experiments presenting a clear contradiction to the CLSM measurements, which suggest a high content of resilin in the forelegs of *S. lineola*. The light microscopy images show that the forelegs of *G. gongylodes* are highly pigmented. Potentially this pigmentation affects the autofluorescence signals.

Highly sclerotized areas show autofluorescence in the red and dark red regions of spectrum. In the here studied forelegs, the tips of the spines showed a strong red signal and are likely harder and stiffer, while the spine bases showed light blue autofluorescence, which may indicate a higher resilin content and thus softer and more flexible regions. This relationship, which was previously also determined for the mandibles of these species^19^, is confirmed by our nanoindentation tests on the tibial spur (type 9), which possessed a softer and flexibles basis and a harder and stiffer tip.

Insect cuticle typically contains low proportions of inorganic materials, but various transition metals as copper (Cu), iron (Fe), manganese (Mn), and zinc (Zn) along with halogens as sodium (Cl) and alkaline earth metals as calcium (Ca) and magnesium (Mg) have been previously found^8–11,25,28,32,33,80,117–122^. Ions, such as Ca, Cu, Fe, Mg, Mn, and Zn, likely serve as covalent cross-linkers^27,35,80,120,123–126^, while Ca and Mg may also exist in crystalline form, contributing to biomineralization^26–28,37,40,101^. Transition and alkaline earth metals may co-occur with elements, such as phosphorus (P), silicon (Si), Cl, potassium (K), or sodium (Na) in the same cuticular region^8,28,37,83,99,100,122,127–131^. Increased hardness and stiffness of insect cuticle is often linked to the presence of elements like Zn, Mn, Ca, and Mg^9,10,15,25,32,80,83,84,95,131^. Interestingly, in both species studied here, we found that Young’s modulus and hardness did not correlate with any of the elements. Probably, the mechanical property gradients have their origin in the degree of tanning. Potentially, also the microstructure (i.e., the density and distribution of chitin fibres) relates to both parameters; this however awaits further investigations. In our previous study on the mandibles of these specimens, we found that the mechanical property values correlated strongly with Mg content^19^. This indicates that different genes are involved in the cuticle formation of the distinct body regions, but awaits further investigations.

## Conclusions

This study represents the first comprehensive examination of the foreleg cuticle of mantises, specifically analysing *Sphodromantis lineola* and *Gongylus gongylodes*. The (I) morphology, (II) autofluorescence, (III) elemental composition, and (iv) mechanical properties were investigated.

*G. gongylodes* forelegs are thinner, which could potentially contribute to the drag force reduction during prey capture. In contrast, *S. lineola* forelegs were composed of a thicker cuticle, which potentially decreases the risk of structural failure, when experiencing high stress. *G. gongylodes* exhibited a higher number of spines, which were longer, more delicate, and positioned more closely together, if compared to those in *S. lineola*. This morphological trait suggests a potential adaptation to a diet comprising smaller insects. In contrast, *S. lineola* possesses a larger number of movable spines, which are potentially used to clamp prey, allowing it to capture a wider range of insects with varying leg thicknesses.

A direct link between the degree of tanning in the forelegs to the dietary preferences could not be established through confocal laser scanning microscopy (CLSM), as the observed differences in autofluorescence between the two species were found to be presumably artefacts resulting from the cuticle pigmentation. This conclusion was reinforced by the results of nanoindentation, which showed that the forelegs of *G. gongylodes* were softer and more flexible than the forelegs of *S. lineola*, which also seems to relate to the preferred prey. Consequently, CLSM measurements should be complemented with additional methods such as light microscopy and the assessment of mechanical properties. In the spines and along the tibial spur, which were not highly pigmented, micro-gradients based on varying tanning and protein composition documented via CSLM were supported by the biomechanical data from nanoindentation.

The analysis of elemental composition provides the first dataset concerning alkaline earth metals, transition metals, and halogens within the forelegs of mantises. Although some differences were documented between species, we did not find a clear pattern and no relationship to the mechanical property values.

## Electronic supplementary material

Below is the link to the electronic supplementary material.

Supplementary Material 1

Supplementary Material 2

Supplementary Material 3

## Data Availability

Data is provided within the manuscript or supplementary information files.
