# Supplementary material for: Morphology and material composition of raptorial foreleg cuticles in praying mantises Gongylus gongylodes and Sphodromantis lineola
Source: Sci Rep. 2025 Jun 20;15:20208. doi: 10.1038/s41598-025-06427-6 (PMC12181396; doi:10.1038/s41598-025-06427-6)
Supplement: Supplementary file 3 — Supplementary Material 3 [file 41598_2025_6427_MOESM3_ESM.docx]

**Morphology and material composition of raptorial foreleg cuticles in praying mantises *Gongylus gongylodes* and *Sphodromantis lineola***

**Timo Zeimet^1,2^, Stanislav N. Gorb^3^, Wencke Krings^1,2,3,4*^**

^1^Department of Electron Microscopy, Institute of Cell and Systems Biology of Animals, Universität Hamburg, Martin-Luther-King-Platz 3, 20146 Hamburg, Germany

^2^Section Mammalogy and Palaeoanthropology, Leibniz Institute for the Analysis of Biodiversity Change, Martin-Luther-King-Platz 3, 20146 Hamburg, Germany

^3^Department of Functional Morphology and Biomechanics, Zoological Institute, Christian-Albrechts-Universität zu Kiel, Am Botanischen Garten 1–9, 24118 Kiel, Germany

^4^Department of Cariology, Endodontology and Periodontology, Universität Leipzig, Liebigstraße 12, 04103 Leipzig, Germany

*corresponding author: wencke.krings@uni-hamburg.de, orcid: 0000-0003-2158-9806

**Supplementary Material**

**Supplementary Table 1.** Overview of the sources and the methodological use of each studied specimen. Abbreviations: CLSM, confocal laser scanning microscopy; EDX, energy-dispersive X-ray spectroscopy; f, female; m, male; SEM, scanning electron microscopy.

| **Animal**  **number** | **Species** | | **Sex** | **Source** | | **Methods** | | | | |
| --- | --- | --- | --- | --- | --- | --- | --- | --- | --- | --- |
|  | ***Sphodromantis lineola*** | ***Gongylus gongylodes*** |  | **mantidenundmehr.de** | **Kai Schütte** | **Habitus**  **images** | **Light**  **microscopy** | **CLSM** | **SEM** | **EDX** |
| 01 | x |  | m | x |  | x | x | x | x | x |
| 02 | x |  | m | x |  |  | x |  | x |  |
| 03 | x |  | m | x |  |  | x | x |  |  |
| 04 | x |  | f | x |  |  | x |  |  |  |
| 05 | x |  | f | x |  |  | x | x |  |  |
| 06 | x |  | f | x |  | x | x | x | x | x |
| 07 | x |  | f | x |  |  | x |  | x |  |
| 08 |  | x | m | x |  |  | x | x |  |  |
| 09 |  | x | m |  | x |  | x |  | x |  |
| 10 |  | x | m |  | x |  | x |  |  |  |
| 11 |  | x | f |  | x |  | x | x | x |  |
| 12 |  | x | f |  | x |  | x |  |  |  |
| 13 |  | x | f |  | x | x | x | x |  |  |
| 14 |  | x | f |  | x | x | x |  | x | x |
| 15 |  | x | m |  | x | x | x | x | x | x |

**Supplementary Table 2. Rank of the elements, from highest to lowest occurrence, based on their atomic %. This is show for each species and for males or females. For each species, we tested one male and one female. Abbreviations: Ca, calcium; Cl, chlorine; Cu, copper; F, fluorine; Fe, iron; K, potassium; Mg, magnesium; Mn, manganese; Na, sodium; P+Pt, sum of phosphorus and platinum; S, sulphur; Si, silicon;** Te, trace elements, sum of Ca, Cl, Cu, F, Fe, K, Mg, Mn, Na, P+Pt, S, Si, and Zn**; Zn, zinc.**

| **Specimens** | **Ranks** | | | | | | | | | | | | | |
| --- | --- | --- | --- | --- | --- | --- | --- | --- | --- | --- | --- | --- | --- | --- |
|  | 1 | 2 | 3 | 4 | 5 | 6 | 7 | 8 | 9 | 10 | 11 | 12 | 13 | 14 |
| Data from both species taken together | Te | F | P+Pt | Mg | Cu | Cl | Zn | Ca | S | Fe | K | Si | Mn | Na |
| Data from both *Gongylus gongylodes* | Te | F | P+Pt | Mg | Cu | Cl | Zn | S | Fe | K | Ca | Mn | Si | Na |
| Female *Gongylus gongylodes* | Te | F | P+Pt | Mg | Cl | Cu | Zn | S | Fe | K | Mn | Ca | Si | Na |
| Male *Gongylus gongylodes* | Te | F | P+Pt | Mg | Cu | Zn | Cl | S | Fe | Ca | Mn | K | Si | Na |
| Data from both *Sphodromantis lineola* | Te | F | P+Pt | Mg | Cu | Cl | Zn | Ca | Si | Fe | K | S | Mn | Na |
| Female *Sphodromantis lineola* | Te | F | P+Pt | Mg | Cl | Cu | Zn | Si | K | Fe | Ca | S | Mn | Na |
| Male *Sphodromantis lineola* | Te | F | P+Pt | Cu | Mg | Zn | Ca | Cl | Fe | S | Si | Mn | K | Na |

**Supplementary Table 3. Results of EDX analysis, given in atomic %, for each species. Results of pairwise comparison between *Gongylus gongylodes* and *Sphodromantis lineola* for each element. Colours of the p-values: black, not significant; yellow, highly significant. For each species, we tested one male and one female. Abbreviations: Cl, chlorine; Cu, copper; F, fluorine; Fe, iron; K, potassium; Mg, magnesium; Mn, manganese; N, number of EDX measurements; Na, sodium; P+Pt, sum of phosphorus and platinum; S, sulphur; SD, standard deviation; Si, silicon;** Te, trace elements, sum of Ca, Cl, Cu, F, Fe, K, Mg, Mn, Na, P+Pt, S, Si, and Zn**; Zn, zinc.**

| **Species** | **N** | **Element** | **Mean** | **SD** | **N** | **Mann-Whitney/Wilcoxon-test** | **Kolomogorov-Smirnov-test** |
| --- | --- | --- | --- | --- | --- | --- | --- |
|  |  |  |  |  |  | **p-value** | **p-value** |
| *Gongylus gongylodes* | 599 | Te | 1.60 | 0.56 | 1157 | <.0001* | <.0001* |
| *Sphodromantis lineola* | 558 |  | 1.50 | 0.46 |  |  |  |
| *Gongylus gongylodes* | 599 | Ca | 0.03 | 0.03 |  | 0.5688 | 0.976 |
| *Sphodromantis lineola* | 558 |  | 0.04 | 0.08 |  |  |  |
| *Gongylus gongylodes* | 599 | Cl | 0.04 | 0.04 |  | 0.3271 | 0.0844 |
| *Sphodromantis lineola* | 558 |  | 0.05 | 0.06 |  |  |  |
| *Gongylus gongylodes* | 599 | Cu | 0.05 | 0.03 |  | 0.1508 | 0.2526 |
| *Sphodromantis lineola* | 558 |  | 0.06 | 0.11 |  |  |  |
| *Gongylus gongylodes* | 599 | F | 0.95 | 0.30 |  | <.0001* | <.0001* |
| *Sphodromantis lineola* | 558 |  | 0.88 | 0.26 |  |  |  |
| *Gongylus gongylodes* | 599 | Fe | 0.03 | 0.01 |  | <.0001* | 0.0052* |
| *Sphodromantis lineola* | 558 |  | 0.03 | 0.01 |  |  |  |
| *Gongylus gongylodes* | 599 | K | 0.03 | 0.04 |  | 0.0083* | 0.0276* |
| *Sphodromantis lineola* | 558 |  | 0.03 | 0.02 |  |  |  |
| *Gongylus gongylodes* | 599 | Mg | 0.08 | 0.06 |  | 0.0357* | 0.0798 |
| *Sphodromantis lineola* | 558 |  | 0.07 | 0.04 |  |  |  |
| *Gongylus gongylodes* | 599 | Mn | 0.03 | 0.01 |  | 0.0062* | 0.0963 |
| *Sphodromantis lineola* | 558 |  | 0.02 | 0.01 |  |  |  |
| *Gongylus gongylodes* | 599 | Na | 0.00 | 0.01 |  | 0.4791 | 1 |
| *Sphodromantis lineola* | 558 |  | 0.00 | 0.00 |  |  |  |
| *Gongylus gongylodes* | 599 | P+Pt | 0.26 | 0.17 |  | <.0001* | <.0001* |
| *Sphodromantis lineola* | 558 |  | 0.21 | 0.10 |  |  |  |
| *Gongylus gongylodes* | 599 | S | 0.03 | 0.04 |  | <.0001* | 0.0062* |
| *Sphodromantis lineola* | 558 |  | 0.03 | 0.02 |  |  |  |
| *Gongylus gongylodes* | 599 | Si | 0.02 | 0.03 |  | 0.0003* | 0.0029* |
| *Sphodromantis lineola* | 558 |  | 0.03 | 0.11 |  |  |  |
| *Gongylus gongylodes* | 599 | Zn | 0.04 | 0.03 |  | 0.8135 | 0.981 |
| *Sphodromantis lineola* | 558 |  | 0.05 | 0.08 |  |  |  |

**Supplementary Table 4. Results of the EDX analysis, given in atomic %, for each specimen. For each species, we tested one male and one female. Abbreviations: Ca, calcium; Cl, chlorine; Cu, copper; F, fluorine; Fe, iron; K, potassium; Mg, magnesium; Mn, manganese; N, number of EDX measurements; Na, sodium; P+Pt, sum of phosphorus and platinum; S, sulphur; SD, standard deviation; Si, silicon;** Te, trace elements, sum of Ca, Cl, Cu, F, Fe, K, Mg, Mn, Na, P+Pt, S, Si, and Zn**; Zn, zinc.**

| **Specimen** | **N** | **Element** | **Mean** | **SD** |
| --- | --- | --- | --- | --- |
| *Gongylus gongylodes* Female | 303 | Te | 1.49 | 0.59 |
|  |  | Ca | 0.02 | 0.03 |
|  |  | Cl | 0.05 | 0.04 |
|  |  | Cu | 0.05 | 0.04 |
|  |  | F | 0.86 | 0.27 |
|  |  | Fe | 0.03 | 0.01 |
|  |  | K | 0.03 | 0.05 |
|  |  | Mg | 0.07 | 0.07 |
|  |  | Mn | 0.03 | 0.02 |
|  |  | Na | 0.00 | 0.01 |
|  |  | P+Pt | 0.23 | 0.18 |
|  |  | S | 0.03 | 0.03 |
|  |  | Si | 0.01 | 0.01 |
|  |  | Zn | 0.04 | 0.03 |
| *Gongylus gongylodes* Male | 296 | Te | 1.72 | 0.49 |
|  |  | Ca | 0.03 | 0.03 |
|  |  | Cl | 0.04 | 0.03 |
|  |  | Cu | 0.05 | 0.03 |
|  |  | F | 1.03 | 0.30 |
|  |  | Fe | 0.03 | 0.01 |
|  |  | K | 0.02 | 0.03 |
|  |  | Mg | 0.08 | 0.05 |
|  |  | Mn | 0.02 | 0.01 |
|  |  | Na | 0.00 | 0.01 |
|  |  | P+Pt | 0.28 | 0.15 |
|  |  | S | 0.04 | 0.04 |
|  |  | Si | 0.02 | 0.04 |
|  |  | Zn | 0.05 | 0.03 |
| *Sphodromantis lineola*  Female | 298 | Te | 1.53 | 0.47 |
|  |  | Ca | 0.03 | 0.03 |
|  |  | Cl | 0.07 | 0.08 |
|  |  | Cu | 0.05 | 0.05 |
|  |  | F | 0.90 | 0.31 |
|  |  | Fe | 0.03 | 0.01 |
|  |  | K | 0.03 | 0.03 |
|  |  | Mg | 0.07 | 0.04 |
|  |  | Mn | 0.02 | 0.01 |
|  |  | Na | 0.00 | 0.00 |
|  |  | P+Pt | 0.21 | 0.12 |
|  |  | S | 0.02 | 0.02 |
|  |  | Si | 0.04 | 0.15 |
|  |  | Zn | 0.04 | 0.03 |
| *Sphodromantis lineola*  Male | 260 | Te | 1.47 | 0.43 |
|  |  | Ca | 0.05 | 0.12 |
|  |  | Cl | 0.04 | 0.03 |
|  |  | Cu | 0.07 | 0.14 |
|  |  | F | 0.84 | 0.19 |
|  |  | Fe | 0.03 | 0.01 |
|  |  | K | 0.02 | 0.01 |
|  |  | Mg | 0.06 | 0.04 |
|  |  | Mn | 0.02 | 0.01 |
|  |  | Na | 0.00 | 0.00 |
|  |  | P+Pt | 0.20 | 0.06 |
|  |  | S | 0.03 | 0.02 |
|  |  | Si | 0.03 | 0.05 |
|  |  | Zn | 0.06 | 0.10 |

**Supplementary Table 5. Pairwise comparison between the male and female *Sphodromantis lineola* for each element. Colours of the p-values: black, not significant; yellow, highly significant. We tested one male and one female. Abbreviations: Cl, chlorine; Cu, copper; f, female; F, fluorine; Fe, iron; K, potassium; m, male; Mg, magnesium; Mn, manganese; N, number of EDX measurements; Na, sodium; P+Pt, sum of phosphorus and platinum; S, sulphur; Si, silicon;** Te, trace elements, sum of Ca, Cl, Cu, F, Fe, K, Mg, Mn, Na, P+Pt, S, Si, and Zn**; Zn, zinc.**

| **Sex** | **Element** | **N** | **Mann-Whitney/****Wilcoxon-test** | **Kolomogorov-Smirnov-test** |
| --- | --- | --- | --- | --- |
|  |  |  | **p-value** | **p-value** |
| m | Te | 558 | 0.0101* | 0.0518 |
| f |  |  |  |  |
| m | Ca |  | 0.8887 | 0.9173 |
| f |  |  |  |  |
| m | Cl |  | <.0001* | <.0001* |
| f |  |  |  |  |
| m | Cu |  | <.0001* | <.0001* |
| f |  |  |  |  |
| m | F |  | 0.0101* | 0.0402* |
| f |  |  |  |  |
| m | Fe |  | 0.2994 | 0.9631 |
| f |  |  |  |  |
| m | K |  | <.0001* | 0.0002* |
| f |  |  |  |  |
| m | Mg |  | 0.0005* | 0.0030* |
| f |  |  |  |  |
| m | Mn |  | 0.822 | 1 |
| f |  |  |  |  |
| m | Na |  | 0.5898 | 1 |
| f |  |  |  |  |
| m | P+Pt |  | 0.9633 | 1 |
| f |  |  |  |  |
| m | S |  | 0.0002* | 0.0015* |
| f |  |  |  |  |
| m | Si |  | 0.3551 | 0.2586 |
| f |  |  |  |  |
| m | Zn |  | <.0001* | 0.0023* |
| f |  |  |  |  |

**Supplementary Table 6. Pairwise comparison between the male and female** *Gongylus gongylodes* **for each element. Colours of the p-values: black, not significant; yellow, highly significant. We tested one male and one female. Abbreviations: Ca, calcium; Cl, chlorine; Cu, copper; f, female; F, fluorine; Fe, iron; K, potassium; m, male; Mg, magnesium; Mn, manganese; N, number of EDX measurements; Na, sodium; P+Pt, sum of phosphorus and platinum; S, sulphur; Si, silicon;** Te, trace elements, sum of Ca, Cl, Cu, F, Fe, K, Mg, Mn, Na, P+Pt, S, Si, and Zn**; Zn, zinc.**

| **Sex** | **Element** | **N** | **Mann-Whitney/Wilcoxon-Test** | **Kolomogorov-Smirnov-Test** |
| --- | --- | --- | --- | --- |
|  |  |  | **p-wert** | **p-wert** |
| m | Te | 599 | <.0001* | <.0001* |
| f |  |  |  |  |
| m | Ca |  | 0.1235 | 0.143 |
| f |  |  |  |  |
| m | Cl |  | 0.0013* | 0.0061* |
| f |  |  |  |  |
| m | Cu |  | 0.0288* | 0.0174* |
| f |  |  |  |  |
| m | F |  | <.0001* | <.0001* |
| f |  |  |  |  |
| m | Fe |  | 0.0290* | 0.2112 |
| f |  |  |  |  |
| m | K |  | 0.1126 | 0.4054 |
| f |  |  |  |  |
| m | Mg |  | <.0001* | <.0001* |
| f |  |  |  |  |
| m | Mn |  | 0.9262 | 0.9991 |
| f |  |  |  |  |
| m | Na |  | 0.4293 | 1 |
| f |  |  |  |  |
| m | P+Pt |  | <.0001* | <.0001* |
| f |  |  |  |  |
| m | S |  | 0.8119 | 0.7654 |
| f |  |  |  |  |
| m | Si |  | 0.0012* | 0.0020* |
| f |  |  |  |  |
| m | Zn |  | 0.0017* | 0.0123* |
| f |  |  |  |  |

**Supplementary Table 7. Results of the EDX analysis, given in atomic %, for the different regions of each specimen. For each species, we tested one male and one female. Abbreviations:** avts, anteroventral tibial spines; **Ca, calcium; Cl, chlorine; Cu, copper;** ds1, 1th discoidal spine; ds2, 2nd discoidal spine; ds3, 3rd discoidal spine; ds4, 4th discoidal spine; **F, fluorine; Fe, iron; K, potassium;** l avfs, long anteroventral femoral spines; **Mg, magnesium; Mn, manganese; N, number of EDX measurements; Na, sodium;** nt avfs, non-tiltable anteroventral femoral spines; **P+Pt, sum of phosphorus and platinum;** pvfs, posteroventral femoral spines; s avfs, short anteroventral femoral spines; **S, sulphur; SD, standard deviation; Si, silicon;** t avfs, tiltable anteroventral femoral spines; Te, trace elements, sum of Ca, Cl, Cu, F, Fe, K, Mg, Mn, Na, P+Pt, S, Si, and Zn**;** ts, tibial spur; **Zn, zinc.**

| ***Sphodromantis* lineola** | | | | | | | **Gongylus gongylodes** | | | | | | |
| --- | --- | --- | --- | --- | --- | --- | --- | --- | --- | --- | --- | --- | --- |
| **Sex** | **Structure** | **Region** | **Element** | **N** | **Mean** | **SD** | **Sex** | **Structure** | **Region** | **Element** | **N** | **Mean** | **SD** |
| Male | Femur | 1 | Te | 16 | 1.45 | 0.20 | Male | Femur | 1 | Te | 29 | 1.60 | 0.54 |
| Male | Femur | 1 | Ca | 16 | 0.01 | 0.01 | Male | Femur | 1 | Ca | 29 | 0.02 | 0.01 |
| Male | Femur | 1 | Cl | 16 | 0.02 | 0.01 | Male | Femur | 1 | Cl | 29 | 0.03 | 0.02 |
| Male | Femur | 1 | Cu | 16 | 0.05 | 0.02 | Male | Femur | 1 | Cu | 29 | 0.06 | 0.04 |
| Male | Femur | 1 | F | 16 | 0.05 | 0.16 | Male | Femur | 1 | F | 29 | 0.99 | 0.35 |
| Male | Femur | 1 | Fe | 16 | 0.03 | 0.02 | Male | Femur | 1 | Fe | 29 | 0.03 | 0.01 |
| Male | Femur | 1 | K | 16 | 0.02 | 0.01 | Male | Femur | 1 | K | 29 | 0.02 | 0.01 |
| Male | Femur | 1 | Mg | 16 | 0.07 | 0.02 | Male | Femur | 1 | Mg | 29 | 0.06 | 0.04 |
| Male | Femur | 1 | Mn | 16 | 0.02 | 0.00 | Male | Femur | 1 | Mn | 29 | 0.03 | 0.02 |
| Male | Femur | 1 | Na | 16 | 0.00 | 0.00 | Male | Femur | 1 | Na | 29 | 0.00 | 0.00 |
| Male | Femur | 1 | P+Pt | 16 | 0.19 | 0.03 | Male | Femur | 1 | P+Pt | 29 | 0.24 | 0.16 |
| Male | Femur | 1 | S | 16 | 0.02 | 0.01 | Male | Femur | 1 | S | 29 | 0.06 | 0.08 |
| Male | Femur | 1 | Si | 16 | 0.02 | 0.01 | Male | Femur | 1 | Si | 29 | 0.01 | 0.01 |
| Male | Femur | 1 | Zn | 16 | 0.04 | 0.02 | Male | Femur | 1 | Zn | 29 | 0.06 | 0.05 |
| Male | Femur | 2 | Te | 16 | 2.31 | 1.03 | Male | Femur | 3 | Te | 7 | 1.25 | 0.36 |
| Male | Femur | 2 | Ca | 16 | 0.40 | 0.29 | Male | Femur | 3 | Ca | 7 | 0.02 | 0.01 |
| Male | Femur | 2 | Cl | 16 | 0.01 | 0.01 | Male | Femur | 3 | Cl | 7 | 0.05 | 0.03 |
| Male | Femur | 2 | Cu | 16 | 0.29 | 0.50 | Male | Femur | 3 | Cu | 7 | 0.03 | 0.02 |
| Male | Femur | 2 | F | 16 | 0.93 | 0.15 | Male | Femur | 3 | F | 7 | 0.75 | 0.33 |
| Male | Femur | 2 | Fe | 16 | 0.03 | 0.01 | Male | Femur | 3 | Fe | 7 | 0.03 | 0.01 |
| Male | Femur | 2 | K | 16 | 0.03 | 0.02 | Male | Femur | 3 | K | 7 | 0.02 | 0.01 |
| Male | Femur | 2 | Mg | 16 | 0.06 | 0.03 | Male | Femur | 3 | Mg | 7 | 0.04 | 0.02 |
| Male | Femur | 2 | Mn | 16 | 0.02 | 0.01 | Male | Femur | 3 | Mn | 7 | 0.03 | 0.01 |
| Male | Femur | 2 | Na | 16 | 0.00 | 0.00 | Male | Femur | 3 | Na | 7 | 0.00 | 0.01 |
| Male | Femur | 2 | P+Pt | 16 | 0.19 | 0.04 | Male | Femur | 3 | P+Pt | 7 | 0.22 | 0.15 |
| Male | Femur | 2 | S | 16 | 0.06 | 0.02 | Male | Femur | 3 | S | 7 | 0.03 | 0.02 |
| Male | Femur | 2 | Si | 16 | 0.07 | 0.09 | Male | Femur | 3 | Si | 7 | 0.01 | 0.01 |
| Male | Femur | 2 | Zn | 16 | 0.23 | 0.35 | Male | Femur | 3 | Zn | 7 | 0.03 | 0.02 |
| Male | Femur | 3 | Te | 19 | 1.52 | 0.36 | Male | Femur | 4 | Te | 27 | 1.96 | 0.45 |
| Male | Femur | 3 | Ca | 19 | 0.03 | 0.02 | Male | Femur | 4 | Ca | 27 | 0.02 | 0.01 |
| Male | Femur | 3 | Cl | 19 | 0.04 | 0.05 | Male | Femur | 4 | Cl | 27 | 0.02 | 0.02 |
| Male | Femur | 3 | Cu | 19 | 0.11 | 0.12 | Male | Femur | 4 | Cu | 27 | 0.05 | 0.04 |
| Male | Femur | 3 | F | 19 | 0.79 | 0.28 | Male | Femur | 4 | F | 27 | 1.27 | 0.37 |
| Male | Femur | 3 | Fe | 19 | 0.03 | 0.01 | Male | Femur | 4 | Fe | 27 | 0.03 | 0.01 |
| Male | Femur | 3 | K | 19 | 0.03 | 0.03 | Male | Femur | 4 | K | 27 | 0.01 | 0.01 |
| Male | Femur | 3 | Mg | 19 | 0.07 | 0.05 | Male | Femur | 4 | Mg | 27 | 0.12 | 0.04 |
| Male | Femur | 3 | Mn | 19 | 0.02 | 0.01 | Male | Femur | 4 | Mn | 27 | 0.02 | 0.01 |
| Male | Femur | 3 | Na | 19 | 0.00 | 0.00 | Male | Femur | 4 | Na | 27 | 0.00 | 0.00 |
| Male | Femur | 3 | P+Pt | 19 | 0.22 | 0.09 | Male | Femur | 4 | P+Pt | 27 | 0.29 | 0.13 |
| Male | Femur | 3 | S | 19 | 0.05 | 0.02 | Male | Femur | 4 | S | 27 | 0.04 | 0.02 |
| Male | Femur | 3 | Si | 19 | 0.02 | 0.01 | Male | Femur | 4 | Si | 27 | 0.02 | 0.01 |
| Male | Femur | 3 | Zn | 19 | 0.09 | 0.08 | Male | Femur | 4 | Zn | 27 | 0.04 | 0.03 |
| Male | Femur | 4 | Te | 27 | 1.55 | 0.44 | Male | Femur | 5 | Te | 33 | 2.09 | 0.40 |
| Male | Femur | 4 | Ca | 27 | 0.02 | 0.01 | Male | Femur | 5 | Ca | 33 | 0.04 | 0.03 |
| Male | Femur | 4 | Cl | 27 | 0.04 | 0.03 | Male | Femur | 5 | Cl | 33 | 0.05 | 0.03 |
| Male | Femur | 4 | Cu | 27 | 0.10 | 0.13 | Male | Femur | 5 | Cu | 33 | 0.06 | 0.03 |
| Male | Femur | 4 | F | 27 | 0.83 | 0.18 | Male | Femur | 5 | F | 33 | 1.18 | 0.24 |
| Male | Femur | 4 | Fe | 27 | 0.03 | 0.01 | Male | Femur | 5 | Fe | 33 | 0.04 | 0.01 |
| Male | Femur | 4 | K | 27 | 0.02 | 0.01 | Male | Femur | 5 | K | 33 | 0.02 | 0.01 |
| Male | Femur | 4 | Mg | 27 | 0.08 | 0.05 | Male | Femur | 5 | Mg | 33 | 0.09 | 0.05 |
| Male | Femur | 4 | Mn | 27 | 0.02 | 0.01 | Male | Femur | 5 | Mn | 33 | 0.03 | 0.01 |
| Male | Femur | 4 | Na | 27 | 0.00 | 0.00 | Male | Femur | 5 | Na | 33 | 0.00 | 0.01 |
| Male | Femur | 4 | P+Pt | 27 | 0.21 | 0.06 | Male | Femur | 5 | P+Pt | 33 | 0.42 | 0.18 |
| Male | Femur | 4 | S | 27 | 0.03 | 0.01 | Male | Femur | 5 | S | 33 | 0.04 | 0.05 |
| Male | Femur | 4 | Si | 27 | 0.04 | 0.10 | Male | Femur | 5 | Si | 33 | 0.02 | 0.03 |
| Male | Femur | 4 | Zn | 27 | 0.08 | 0.09 | Male | Femur | 5 | Zn | 33 | 0.05 | 0.03 |
| Male | Femur | 5 | Te | 20 | 1.41 | 0.25 | Male | Tibia | 1 | Te | 15 | 1.70 | 0.08 |
| Male | Femur | 5 | Ca | 20 | 0.03 | 0.02 | Male | Tibia | 1 | Ca | 15 | 0.01 | 0.01 |
| Male | Femur | 5 | Cl | 20 | 0.03 | 0.01 | Male | Tibia | 1 | Cl | 15 | 0.01 | 0.01 |
| Male | Femur | 5 | Cu | 20 | 0.05 | 0.02 | Male | Tibia | 1 | Cu | 15 | 0.04 | 0.03 |
| Male | Femur | 5 | F | 20 | 0.78 | 0.15 | Male | Tibia | 1 | F | 15 | 1.25 | 0.11 |
| Male | Femur | 5 | Fe | 20 | 0.03 | 0.01 | Male | Tibia | 1 | Fe | 15 | 0.02 | 0.01 |
| Male | Femur | 5 | K | 20 | 0.02 | 0.02 | Male | Tibia | 1 | K | 15 | 0.01 | 0.00 |
| Male | Femur | 5 | Mg | 20 | 0.07 | 0.04 | Male | Tibia | 1 | Mg | 15 | 0.10 | 0.01 |
| Male | Femur | 5 | Mn | 20 | 0.02 | 0.01 | Male | Tibia | 1 | Mn | 15 | 0.01 | 0.00 |
| Male | Femur | 5 | Na | 20 | 0.00 | 0.00 | Male | Tibia | 1 | Na | 15 | 0.00 | 0.00 |
| Male | Femur | 5 | P+Pt | 20 | 0.20 | 0.08 | Male | Tibia | 1 | P+Pt | 15 | 0.15 | 0.03 |
| Male | Femur | 5 | S | 20 | 0.03 | 0.02 | Male | Tibia | 1 | S | 15 | 0.03 | 0.01 |
| Male | Femur | 5 | Si | 20 | 0.03 | 0.03 | Male | Tibia | 1 | Si | 15 | 0.02 | 0.01 |
| Male | Femur | 5 | Zn | 20 | 0.05 | 0.03 | Male | Tibia | 1 | Zn | 15 | 0.04 | 0.02 |
| Male | Tibia | 1 | Te | 15 | 1.54 | 0.14 | Male | Tibia | 2 | Te | 11 | 1.73 | 0.19 |
| Male | Tibia | 1 | Ca | 15 | 0.02 | 0.01 | Male | Tibia | 2 | Ca | 11 | 0.02 | 0.01 |
| Male | Tibia | 1 | Cl | 15 | 0.02 | 0.01 | Male | Tibia | 2 | Cl | 11 | 0.02 | 0.01 |
| Male | Tibia | 1 | Cu | 15 | 0.07 | 0.02 | Male | Tibia | 2 | Cu | 11 | 0.09 | 0.04 |
| Male | Tibia | 1 | F | 15 | 0.94 | 0.07 | Male | Tibia | 2 | F | 11 | 0.99 | 0.13 |
| Male | Tibia | 1 | Fe | 15 | 0.03 | 0.01 | Male | Tibia | 2 | Fe | 11 | 0.04 | 0.01 |
| Male | Tibia | 1 | K | 15 | 0.02 | 0.01 | Male | Tibia | 2 | K | 11 | 0.02 | 0.01 |
| Male | Tibia | 1 | Mg | 15 | 0.07 | 0.03 | Male | Tibia | 2 | Mg | 11 | 0.05 | 0.04 |
| Male | Tibia | 1 | Mn | 15 | 0.02 | 0.01 | Male | Tibia | 2 | Mn | 11 | 0.03 | 0.01 |
| Male | Tibia | 1 | Na | 15 | 0.00 | 0.00 | Male | Tibia | 2 | Na | 11 | 0.00 | 0.00 |
| Male | Tibia | 1 | P+Pt | 15 | 0.20 | 0.03 | Male | Tibia | 2 | P+Pt | 11 | 0.36 | 0.13 |
| Male | Tibia | 1 | S | 15 | 0.03 | 0.01 | Male | Tibia | 2 | S | 11 | 0.04 | 0.02 |
| Male | Tibia | 1 | Si | 15 | 0.07 | 0.13 | Male | Tibia | 2 | Si | 11 | 0.01 | 0.01 |
| Male | Tibia | 1 | Zn | 15 | 0.07 | 0.02 | Male | Tibia | 2 | Zn | 11 | 0.07 | 0.02 |
| Male | Tibia | 2 | Te | 16 | 1.43 | 0.10 | Male | Tibia | 3 | Te | 15 | 1.63 | 0.08 |
| Male | Tibia | 2 | Ca | 16 | 0.01 | 0.01 | Male | Tibia | 3 | Ca | 15 | 0.01 | 0.01 |
| Male | Tibia | 2 | Cl | 16 | 0.02 | 0.02 | Male | Tibia | 3 | Cl | 15 | 0.03 | 0.01 |
| Male | Tibia | 2 | Cu | 16 | 0.05 | 0.02 | Male | Tibia | 3 | Cu | 15 | 0.04 | 0.01 |
| Male | Tibia | 2 | F | 16 | 0.92 | 0.11 | Male | Tibia | 3 | F | 15 | 1.05 | 0.12 |
| Male | Tibia | 2 | Fe | 16 | 0.02 | 0.01 | Male | Tibia | 3 | Fe | 15 | 0.03 | 0.01 |
| Male | Tibia | 2 | K | 16 | 0.01 | 0.01 | Male | Tibia | 3 | K | 15 | 0.02 | 0.01 |
| Male | Tibia | 2 | Mg | 16 | 0.09 | 0.03 | Male | Tibia | 3 | Mg | 15 | 0.10 | 0.03 |
| Male | Tibia | 2 | Mn | 16 | 0.02 | 0.01 | Male | Tibia | 3 | Mn | 15 | 0.02 | 0.01 |
| Male | Tibia | 2 | Na | 16 | 0.00 | 0.00 | Male | Tibia | 3 | Na | 15 | 0.00 | 0.00 |
| Male | Tibia | 2 | P+Pt | 16 | 0.18 | 0.06 | Male | Tibia | 3 | P+Pt | 15 | 0.25 | 0.04 |
| Male | Tibia | 2 | S | 16 | 0.02 | 0.01 | Male | Tibia | 3 | S | 15 | 0.02 | 0.01 |
| Male | Tibia | 2 | Si | 16 | 0.03 | 0.03 | Male | Tibia | 3 | Si | 15 | 0.02 | 0.01 |
| Male | Tibia | 2 | Zn | 16 | 0.05 | 0.02 | Male | Tibia | 3 | Zn | 15 | 0.04 | 0.01 |
| Male | Tibia | 3 | Te | 13 | 1.52 | 0.21 | Male | Spine | ds3 | Te | 18 | 1.13 | 0.19 |
| Male | Tibia | 3 | Ca | 13 | 0.07 | 0.06 | Male | Spine | ds3 | Ca | 18 | 0.03 | 0.01 |
| Male | Tibia | 3 | Cl | 13 | 0.05 | 0.02 | Male | Spine | ds3 | Cl | 18 | 0.07 | 0.03 |
| Male | Tibia | 3 | Cu | 13 | 0.04 | 0.01 | Male | Spine | ds3 | Cu | 18 | 0.04 | 0.01 |
| Male | Tibia | 3 | F | 13 | 0.95 | 0.13 | Male | Spine | ds3 | F | 18 | 0.61 | 0.16 |
| Male | Tibia | 3 | Fe | 13 | 0.03 | 0.01 | Male | Spine | ds3 | Fe | 18 | 0.03 | 0.01 |
| Male | Tibia | 3 | K | 13 | 0.02 | 0.01 | Male | Spine | ds3 | K | 18 | 0.03 | 0.01 |
| Male | Tibia | 3 | Mg | 13 | 0.03 | 0.03 | Male | Spine | ds3 | Mg | 18 | 0.03 | 0.03 |
| Male | Tibia | 3 | Mn | 13 | 0.02 | 0.01 | Male | Spine | ds3 | Mn | 18 | 0.03 | 0.01 |
| Male | Tibia | 3 | Na | 13 | 0.00 | 0.00 | Male | Spine | ds3 | Na | 18 | 0.00 | 0.00 |
| Male | Tibia | 3 | P+Pt | 13 | 0.21 | 0.03 | Male | Spine | ds3 | P+Pt | 18 | 0.19 | 0.05 |
| Male | Tibia | 3 | S | 13 | 0.03 | 0.01 | Male | Spine | ds3 | S | 18 | 0.02 | 0.01 |
| Male | Tibia | 3 | Si | 13 | 0.02 | 0.03 | Male | Spine | ds3 | Si | 18 | 0.01 | 0.01 |
| Male | Tibia | 3 | Zn | 13 | 0.04 | 0.01 | Male | Spine | ds3 | Zn | 18 | 0.04 | 0.01 |
| Male | Spine | ds1 | Te | 7 | 1.48 | 0.30 | Male | Spine | ds4 | Te | 15 | 1.28 | 0.22 |
| Male | Spine | ds1 | Ca | 7 | 0.03 | 0.03 | Male | Spine | ds4 | Ca | 15 | 0.02 | 0.01 |
| Male | Spine | ds1 | Cl | 7 | 0.03 | 0.01 | Male | Spine | ds4 | Cl | 15 | 0.06 | 0.02 |
| Male | Spine | ds1 | Cu | 7 | 0.05 | 0.02 | Male | Spine | ds4 | Cu | 15 | 0.04 | 0.01 |
| Male | Spine | ds1 | F | 7 | 0.89 | 0.17 | Male | Spine | ds4 | F | 15 | 0.70 | 0.19 |
| Male | Spine | ds1 | Fe | 7 | 0.03 | 0.01 | Male | Spine | ds4 | Fe | 15 | 0.03 | 0.01 |
| Male | Spine | ds1 | K | 7 | 0.02 | 0.00 | Male | Spine | ds4 | K | 15 | 0.03 | 0.01 |
| Male | Spine | ds1 | Mg | 7 | 0.09 | 0.03 | Male | Spine | ds4 | Mg | 15 | 0.04 | 0.03 |
| Male | Spine | ds1 | Mn | 7 | 0.02 | 0.01 | Male | Spine | ds4 | Mn | 15 | 0.03 | 0.01 |
| Male | Spine | ds1 | Na | 7 | 0.00 | 0.00 | Male | Spine | ds4 | Na | 15 | 0.00 | 0.00 |
| Male | Spine | ds1 | P+Pt | 7 | 0.21 | 0.05 | Male | Spine | ds4 | P+Pt | 15 | 0.22 | 0.07 |
| Male | Spine | ds1 | S | 7 | 0.03 | 0.02 | Male | Spine | ds4 | S | 15 | 0.02 | 0.01 |
| Male | Spine | ds1 | Si | 7 | 0.02 | 0.01 | Male | Spine | ds4 | Si | 15 | 0.05 | 0.17 |
| Male | Spine | ds1 | Zn | 7 | 0.05 | 0.03 | Male | Spine | ds4 | Zn | 15 | 0.04 | 0.01 |
| Male | Spine | ds3 | Te | 13 | 1.28 | 0.34 | Male | Spine | pvfs | Te | 25 | 1.56 | 0.17 |
| Male | Spine | ds3 | Ca | 13 | 0.02 | 0.01 | Male | Spine | pvfs | Ca | 25 | 0.02 | 0.01 |
| Male | Spine | ds3 | Cl | 13 | 0.03 | 0.01 | Male | Spine | pvfs | Cl | 25 | 0.02 | 0.02 |
| Male | Spine | ds3 | Cu | 13 | 0.03 | 0.02 | Male | Spine | pvfs | Cu | 25 | 0.05 | 0.02 |
| Male | Spine | ds3 | F | 13 | 0.80 | 0.25 | Male | Spine | pvfs | F | 25 | 1.02 | 0.19 |
| Male | Spine | ds3 | Fe | 13 | 0.03 | 0.01 | Male | Spine | pvfs | Fe | 25 | 0.03 | 0.01 |
| Male | Spine | ds3 | K | 13 | 0.02 | 0.01 | Male | Spine | pvfs | K | 25 | 0.02 | 0.01 |
| Male | Spine | ds3 | Mg | 13 | 0.05 | 0.03 | Male | Spine | pvfs | Mg | 25 | 0.09 | 0.04 |
| Male | Spine | ds3 | Mn | 13 | 0.02 | 0.01 | Male | Spine | pvfs | Mn | 25 | 0.02 | 0.01 |
| Male | Spine | ds3 | Na | 13 | 0.00 | 0.00 | Male | Spine | pvfs | Na | 25 | 0.00 | 0.00 |
| Male | Spine | ds3 | P+Pt | 13 | 0.19 | 0.08 | Male | Spine | pvfs | P+Pt | 25 | 0.22 | 0.05 |
| Male | Spine | ds3 | S | 13 | 0.02 | 0.01 | Male | Spine | pvfs | S | 25 | 0.02 | 0.01 |
| Male | Spine | ds3 | Si | 13 | 0.01 | 0.01 | Male | Spine | pvfs | Si | 25 | 0.02 | 0.01 |
| Male | Spine | ds3 | Zn | 13 | 0.04 | 0.01 | Male | Spine | pvfs | Zn | 25 | 0.04 | 0.02 |
| Male | Spine | ds4 | Te | 11 | 1.16 | 0.22 | Male | Spine | l avfs | Te | 18 | 1.83 | 0.36 |
| Male | Spine | ds4 | Ca | 11 | 0.02 | 0.01 | Male | Spine | l avfs | Ca | 18 | 0.04 | 0.05 |
| Male | Spine | ds4 | Cl | 11 | 0.04 | 0.03 | Male | Spine | l avfs | Cl | 18 | 0.03 | 0.02 |
| Male | Spine | ds4 | Cu | 11 | 0.04 | 0.01 | Male | Spine | l avfs | Cu | 18 | 0.04 | 0.02 |
| Male | Spine | ds4 | F | 11 | 0.71 | 0.20 | Male | Spine | l avfs | F | 18 | 1.12 | 0.15 |
| Male | Spine | ds4 | Fe | 11 | 0.03 | 0.01 | Male | Spine | l avfs | Fe | 18 | 0.03 | 0.01 |
| Male | Spine | ds4 | K | 11 | 0.02 | 0.01 | Male | Spine | l avfs | K | 18 | 0.02 | 0.01 |
| Male | Spine | ds4 | Mg | 11 | 0.04 | 0.02 | Male | Spine | l avfs | Mg | 18 | 0.10 | 0.04 |
| Male | Spine | ds4 | Mn | 11 | 0.03 | 0.01 | Male | Spine | l avfs | Mn | 18 | 0.02 | 0.01 |
| Male | Spine | ds4 | Na | 11 | 0.00 | 0.00 | Male | Spine | l avfs | Na | 18 | 0.00 | 0.00 |
| Male | Spine | ds4 | P+Pt | 11 | 0.16 | 0.05 | Male | Spine | l avfs | P+Pt | 18 | 0.30 | 0.12 |
| Male | Spine | ds4 | S | 11 | 0.02 | 0.01 | Male | Spine | l avfs | S | 18 | 0.04 | 0.07 |
| Male | Spine | ds4 | Si | 11 | 0.01 | 0.01 | Male | Spine | l avfs | Si | 18 | 0.02 | 0.01 |
| Male | Spine | ds4 | Zn | 11 | 0.04 | 0.02 | Male | Spine | l avfs | Zn | 18 | 0.04 | 0.02 |
| Male | Spine | pvfs | Te | 16 | 1.31 | 0.19 | Male | Spine | s avfs | Te | 32 | 1.69 | 0.35 |
| Male | Spine | pvfs | Ca | 16 | 0.02 | 0.01 | Male | Spine | s avfs | Ca | 32 | 0.03 | 0.04 |
| Male | Spine | pvfs | Cl | 16 | 0.05 | 0.04 | Male | Spine | s avfs | Cl | 32 | 0.03 | 0.02 |
| Male | Spine | pvfs | Cu | 16 | 0.03 | 0.01 | Male | Spine | s avfs | Cu | 32 | 0.04 | 0.01 |
| Male | Spine | pvfs | F | 16 | 0.80 | 0.15 | Male | Spine | s avfs | F | 32 | 1.03 | 0.21 |
| Male | Spine | pvfs | Fe | 16 | 0.03 | 0.01 | Male | Spine | s avfs | Fe | 32 | 0.03 | 0.01 |
| Male | Spine | pvfs | K | 16 | 0.02 | 0.01 | Male | Spine | s avfs | K | 32 | 0.02 | 0.01 |
| Male | Spine | pvfs | Mg | 16 | 0.06 | 0.03 | Male | Spine | s avfs | Mg | 32 | 0.10 | 0.04 |
| Male | Spine | pvfs | Mn | 16 | 0.02 | 0.01 | Male | Spine | s avfs | Mn | 32 | 0.02 | 0.01 |
| Male | Spine | pvfs | Na | 16 | 0.00 | 0.00 | Male | Spine | s avfs | Na | 32 | 0.00 | 0.00 |
| Male | Spine | pvfs | P+Pt | 16 | 0.20 | 0.08 | Male | Spine | s avfs | P+Pt | 32 | 0.27 | 0.08 |
| Male | Spine | pvfs | S | 16 | 0.02 | 0.01 | Male | Spine | s avfs | S | 32 | 0.03 | 0.03 |
| Male | Spine | pvfs | Si | 16 | 0.02 | 0.03 | Male | Spine | s avfs | Si | 32 | 0.02 | 0.01 |
| Male | Spine | pvfs | Zn | 16 | 0.03 | 0.01 | Male | Spine | s avfs | Zn | 32 | 0.04 | 0.02 |
| Male | Spine | nt avfs | Te | 15 | 1.33 | 0.21 | Male | Spine | avts | Te | 30 | 2.06 | 0.81 |
| Male | Spine | nt avfs | Ca | 15 | 0.02 | 0.01 | Male | Spine | avts | Ca | 30 | 0.06 | 0.07 |
| Male | Spine | nt avfs | Cl | 15 | 0.09 | 0.04 | Male | Spine | avts | Cl | 30 | 0.08 | 0.06 |
| Male | Spine | nt avfs | Cu | 15 | 0.03 | 0.02 | Male | Spine | avts | Cu | 30 | 0.05 | 0.02 |
| Male | Spine | nt avfs | F | 15 | 0.79 | 0.21 | Male | Spine | avts | F | 30 | 1.03 | 0.30 |
| Male | Spine | nt avfs | Fe | 15 | 0.02 | 0.01 | Male | Spine | avts | Fe | 30 | 0.04 | 0.01 |
| Male | Spine | nt avfs | K | 15 | 0.03 | 0.01 | Male | Spine | avts | K | 30 | 0.06 | 0.06 |
| Male | Spine | nt avfs | Mg | 15 | 0.06 | 0.04 | Male | Spine | avts | Mg | 30 | 0.09 | 0.06 |
| Male | Spine | nt avfs | Mn | 15 | 0.02 | 0.01 | Male | Spine | avts | Mn | 30 | 0.03 | 0.01 |
| Male | Spine | nt avfs | Na | 15 | 0.00 | 0.00 | Male | Spine | avts | Na | 30 | 0.00 | 0.03 |
| Male | Spine | nt avfs | P+Pt | 15 | 0.19 | 0.06 | Male | Spine | avts | P+Pt | 30 | 0.41 | 0.25 |
| Male | Spine | nt avfs | S | 15 | 0.02 | 0.02 | Male | Spine | avts | S | 30 | 0.06 | 0.05 |
| Male | Spine | nt avfs | Si | 15 | 0.01 | 0.01 | Male | Spine | avts | Si | 30 | 0.01 | 0.01 |
| Male | Spine | nt avfs | Zn | 15 | 0.03 | 0.02 | Male | Spine | avts | Zn | 30 | 0.05 | 0.03 |
| Male | Spine | t avfs | Te | 14 | 1.27 | 0.36 | Male | Spine | ts | Te | 21 | 1.69 | 0.40 |
| Male | Spine | t avfs | Ca | 14 | 0.02 | 0.02 | Male | Spine | ts | Ca | 21 | 0.04 | 0.03 |
| Male | Spine | t avfs | Cl | 14 | 0.04 | 0.02 | Male | Spine | ts | Cl | 21 | 0.04 | 0.04 |
| Male | Spine | t avfs | Cu | 14 | 0.04 | 0.01 | Male | Spine | ts | Cu | 21 | 0.04 | 0.02 |
| Male | Spine | t avfs | F | 14 | 0.77 | 0.23 | Male | Spine | ts | F | 21 | 0.99 | 0.22 |
| Male | Spine | t avfs | Fe | 14 | 0.03 | 0.01 | Male | Spine | ts | Fe | 21 | 0.03 | 0.01 |
| Male | Spine | t avfs | K | 14 | 0.02 | 0.01 | Male | Spine | ts | K | 21 | 0.04 | 0.04 |
| Male | Spine | t avfs | Mg | 14 | 0.05 | 0.03 | Male | Spine | ts | Mg | 21 | 0.10 | 0.04 |
| Male | Spine | t avfs | Mn | 14 | 0.02 | 0.01 | Male | Spine | ts | Mn | 21 | 0.02 | 0.01 |
| Male | Spine | t avfs | Na | 14 | 0.00 | 0.00 | Male | Spine | ts | Na | 21 | 0.00 | 0.00 |
| Male | Spine | t avfs | P+Pt | 14 | 0.19 | 0.07 | Male | Spine | ts | P+Pt | 21 | 0.25 | 0.08 |
| Male | Spine | t avfs | S | 14 | 0.02 | 0.02 | Male | Spine | ts | S | 21 | 0.03 | 0.02 |
| Male | Spine | t avfs | Si | 14 | 0.01 | 0.01 | Male | Spine | ts | Si | 21 | 0.02 | 0.01 |
| Male | Spine | t avfs | Zn | 14 | 0.04 | 0.02 | Male | Spine | ts | Zn | 21 | 0.05 | 0.02 |
| Male | Spine | avts | Te | 17 | 1.26 | 0.25 | Female | Femur | 1 | Te | 15 | 1.67 | 0.43 |
| Male | Spine | avts | Ca | 17 | 0.02 | 0.01 | Female | Femur | 1 | Ca | 15 | 0.02 | 0.01 |
| Male | Spine | avts | Cl | 17 | 0.04 | 0.02 | Female | Femur | 1 | Cl | 15 | 0.03 | 0.02 |
| Male | Spine | avts | Cu | 17 | 0.04 | 0.02 | Female | Femur | 1 | Cu | 15 | 0.32 | 0.29 |
| Male | Spine | avts | F | 17 | 0.76 | 0.19 | Female | Femur | 1 | F | 15 | 0.96 | 0.18 |
| Male | Spine | avts | Fe | 17 | 0.03 | 0.01 | Female | Femur | 1 | Fe | 15 | 0.03 | 0.01 |
| Male | Spine | avts | K | 17 | 0.02 | 0.01 | Female | Femur | 1 | K | 15 | 0.02 | 0.01 |
| Male | Spine | avts | Mg | 17 | 0.04 | 0.02 | Female | Femur | 1 | Mg | 15 | 0.06 | 0.03 |
| Male | Spine | avts | Mn | 17 | 0.03 | 0.01 | Female | Femur | 1 | Mn | 15 | 0.03 | 0.02 |
| Male | Spine | avts | Na | 17 | 0.00 | 0.00 | Female | Femur | 1 | Na | 15 | 0.00 | 0.00 |
| Male | Spine | avts | P+Pt | 17 | 0.20 | 0.09 | Female | Femur | 1 | P+Pt | 15 | 0.32 | 0.23 |
| Male | Spine | avts | S | 17 | 0.03 | 0.02 | Female | Femur | 1 | S | 15 | 0.03 | 0.01 |
| Male | Spine | avts | Si | 17 | 0.01 | 0.00 | Female | Femur | 1 | Si | 15 | 0.02 | 0.01 |
| Male | Spine | avts | Zn | 17 | 0.50 | 0.02 | Female | Femur | 1 | Zn | 15 | 0.08 | 0.06 |
| Male | Spine | ts | Te | 25 | 1.44 | 0.19 | Female | Femur | 3 | Te | 16 | 1.49 | 0.57 |
| Male | Spine | ts | Ca | 25 | 0.01 | 0.01 | Female | Femur | 3 | Ca | 16 | 0.03 | 0.03 |
| Male | Spine | ts | Cl | 25 | 0.06 | 0.04 | Female | Femur | 3 | Cl | 16 | 0.08 | 0.06 |
| Male | Spine | ts | Cu | 25 | 0.04 | 0.02 | Female | Femur | 3 | Cu | 16 | 0.24 | 0.21 |
| Male | Spine | ts | F | 25 | 0.89 | 0.17 | Female | Femur | 3 | F | 16 | 0.74 | 0.33 |
| Male | Spine | ts | Fe | 25 | 0.03 | 0.01 | Female | Femur | 3 | Fe | 16 | 0.04 | 0.03 |
| Male | Spine | ts | K | 25 | 0.02 | 0.01 | Female | Femur | 3 | K | 16 | 0.04 | 0.05 |
| Male | Spine | ts | Mg | 25 | 0.07 | 0.04 | Female | Femur | 3 | Mg | 16 | 0.07 | 0.09 |
| Male | Spine | ts | Mn | 25 | 0.02 | 0.01 | Female | Femur | 3 | Mn | 16 | 0.03 | 0.05 |
| Male | Spine | ts | Na | 25 | 0.00 | 0.01 | Female | Femur | 3 | Na | 16 | 0.00 | 0.00 |
| Male | Spine | ts | P+Pt | 25 | 0.21 | 0.06 | Female | Femur | 3 | P+Pt | 16 | 0.29 | 0.11 |
| Male | Spine | ts | S | 25 | 0.02 | 0.01 | Female | Femur | 3 | S | 16 | 0.02 | 0.03 |
| Male | Spine | ts | Si | 25 | 0.01 | 0.02 | Female | Femur | 3 | Si | 16 | 0.02 | 0.04 |
| Male | Spine | ts | Zn | 25 | 0.04 | 0.02 | Female | Femur | 3 | Zn | 16 | 0.05 | 0.04 |
| Female | Femur | 1 | Te | 15 | 1.75 | 0.39 | Female | Femur | 4 | Te | 17 | 1.70 | 0.86 |
| Female | Femur | 1 | Ca | 15 | 0.02 | 0.01 | Female | Femur | 4 | Ca | 17 | 0.04 | 0.04 |
| Female | Femur | 1 | Cl | 15 | 0.01 | 0.01 | Female | Femur | 4 | Cl | 17 | 0.03 | 0.03 |
| Female | Femur | 1 | Cu | 15 | 0.08 | 0.09 | Female | Femur | 4 | Cu | 17 | 0.37 | 0.20 |
| Female | Femur | 1 | F | 15 | 0.99 | 0.12 | Female | Femur | 4 | F | 17 | 0.77 | 0.37 |
| Female | Femur | 1 | Fe | 15 | 0.03 | 0.01 | Female | Femur | 4 | Fe | 17 | 0.03 | 0.02 |
| Female | Femur | 1 | K | 15 | 0.02 | 0.01 | Female | Femur | 4 | K | 17 | 0.04 | 0.04 |
| Female | Femur | 1 | Mg | 15 | 0.08 | 0.04 | Female | Femur | 4 | Mg | 17 | 0.12 | 0.11 |
| Female | Femur | 1 | Mn | 15 | 0.02 | 0.01 | Female | Femur | 4 | Mn | 17 | 0.03 | 0.01 |
| Female | Femur | 1 | Na | 15 | 0.00 | 0.00 | Female | Femur | 4 | Na | 17 | 0.00 | 0.01 |
| Female | Femur | 1 | P+Pt | 15 | 0.21 | 0.04 | Female | Femur | 4 | P+Pt | 17 | 0.26 | 0.21 |
| Female | Femur | 1 | S | 15 | 0.02 | 0.01 | Female | Femur | 4 | S | 17 | 0.07 | 0.05 |
| Female | Femur | 1 | Si | 15 | 0.19 | 0.36 | Female | Femur | 4 | Si | 17 | 0.02 | 0.01 |
| Female | Femur | 1 | Zn | 15 | 0.07 | 0.06 | Female | Femur | 4 | Zn | 17 | 0.08 | 0.06 |
| Female | Femur | 2 | Te | 16 | 1.75 | 0.14 | Female | Femur | 5 | Te | 14 | 2.01 | 1.90 |
| Female | Femur | 2 | Ca | 16 | 0.03 | 0.03 | Female | Femur | 5 | Ca | 14 | 0.03 | 0.05 |
| Female | Femur | 2 | Cl | 16 | 0.01 | 0.01 | Female | Femur | 5 | Cl | 14 | 0.06 | 0.03 |
| Female | Femur | 2 | Cu | 16 | 0.08 | 0.03 | Female | Femur | 5 | Cu | 14 | 0.23 | 0.08 |
| Female | Femur | 2 | F | 16 | 1.11 | 0.15 | Female | Femur | 5 | F | 14 | 0.89 | 0.30 |
| Female | Femur | 2 | Fe | 16 | 0.03 | 0.01 | Female | Femur | 5 | Fe | 14 | 0.03 | 0.01 |
| Female | Femur | 2 | K | 16 | 0.02 | 0.01 | Female | Femur | 5 | K | 14 | 0.10 | 0.20 |
| Female | Femur | 2 | Mg | 16 | 0.09 | 0.04 | Female | Femur | 5 | Mg | 14 | 0.14 | 0.23 |
| Female | Femur | 2 | Mn | 16 | 0.02 | 0.01 | Female | Femur | 5 | Mn | 14 | 0.02 | 0.01 |
| Female | Femur | 2 | Na | 16 | 0.00 | 0.00 | Female | Femur | 5 | Na | 14 | 0.00 | 0.00 |
| Female | Femur | 2 | P+Pt | 16 | 0.23 | 0.08 | Female | Femur | 5 | P+Pt | 14 | 0.37 | 0.53 |
| Female | Femur | 2 | S | 16 | 0.03 | 0.01 | Female | Femur | 5 | S | 14 | 0.06 | 0.10 |
| Female | Femur | 2 | Si | 16 | 0.03 | 0.02 | Female | Femur | 5 | Si | 14 | 0.01 | 0.01 |
| Female | Femur | 2 | Zn | 16 | 0.07 | 0.02 | Female | Femur | 5 | Zn | 14 | 0.04 | 0.03 |
| Female | Femur | 3 | Te | 16 | 1.77 | 0.46 | Female | Tibia | 1 | Te | 12 | 1.42 | 0.35 |
| Female | Femur | 3 | Ca | 16 | 0.04 | 0.02 | Female | Tibia | 1 | Ca | 12 | 0.02 | 0.02 |
| Female | Femur | 3 | Cl | 16 | 0.14 | 0.06 | Female | Tibia | 1 | Cl | 12 | 0.02 | 0.01 |
| Female | Femur | 3 | Cu | 16 | 0.06 | 0.03 | Female | Tibia | 1 | Cu | 12 | 0.24 | 0.09 |
| Female | Femur | 3 | F | 16 | 0.86 | 0.28 | Female | Tibia | 1 | F | 12 | 0.91 | 0.26 |
| Female | Femur | 3 | Fe | 16 | 0.03 | 0.01 | Female | Tibia | 1 | Fe | 12 | 0.03 | 0.01 |
| Female | Femur | 3 | K | 16 | 0.08 | 0.04 | Female | Tibia | 1 | K | 12 | 0.02 | 0.01 |
| Female | Femur | 3 | Mg | 16 | 0.06 | 0.03 | Female | Tibia | 1 | Mg | 12 | 0.06 | 0.04 |
| Female | Femur | 3 | Mn | 16 | 0.03 | 0.01 | Female | Tibia | 1 | Mn | 12 | 0.03 | 0.02 |
| Female | Femur | 3 | Na | 16 | 0.00 | 0.00 | Female | Tibia | 1 | Na | 12 | 0.00 | 0.00 |
| Female | Femur | 3 | P+Pt | 16 | 0.26 | 0.06 | Female | Tibia | 1 | P+Pt | 12 | 0.22 | 0.15 |
| Female | Femur | 3 | S | 16 | 0.04 | 0.02 | Female | Tibia | 1 | S | 12 | 0.03 | 0.02 |
| Female | Femur | 3 | Si | 16 | 0.05 | 0.11 | Female | Tibia | 1 | Si | 12 | 0.01 | 0.01 |
| Female | Femur | 3 | Zn | 16 | 0.05 | 0.02 | Female | Tibia | 1 | Zn | 12 | 0.04 | 0.02 |
| Female | Femur | 4 | Te | 12 | 1.69 | 0.34 | Female | Tibia | 2 | Te | 19 | 1.32 | 0.24 |
| Female | Femur | 4 | Ca | 12 | 0.02 | 0.01 | Female | Tibia | 2 | Ca | 19 | 0.02 | 0.01 |
| Female | Femur | 4 | Cl | 12 | 0.12 | 0.05 | Female | Tibia | 2 | Cl | 19 | 0.03 | 0.02 |
| Female | Femur | 4 | Cu | 12 | 0.14 | 0.14 | Female | Tibia | 2 | Cu | 19 | 0.17 | 0.05 |
| Female | Femur | 4 | F | 12 | 0.88 | 0.12 | Female | Tibia | 2 | F | 19 | 0.82 | 0.18 |
| Female | Femur | 4 | Fe | 12 | 0.03 | 0.01 | Female | Tibia | 2 | Fe | 19 | 0.03 | 0.01 |
| Female | Femur | 4 | K | 12 | 0.05 | 0.03 | Female | Tibia | 2 | K | 19 | 0.02 | 0.01 |
| Female | Femur | 4 | Mg | 12 | 0.08 | 0.03 | Female | Tibia | 2 | Mg | 19 | 0.05 | 0.03 |
| Female | Femur | 4 | Mn | 12 | 0.02 | 0.01 | Female | Tibia | 2 | Mn | 19 | 0.02 | 0.01 |
| Female | Femur | 4 | Na | 12 | 0.00 | 0.00 | Female | Tibia | 2 | Na | 19 | 0.00 | 0.00 |
| Female | Femur | 4 | P+Pt | 12 | 0.19 | 0.04 | Female | Tibia | 2 | P+Pt | 19 | 0.20 | 0.06 |
| Female | Femur | 4 | S | 12 | 0.03 | 0.02 | Female | Tibia | 2 | S | 19 | 0.03 | 0.01 |
| Female | Femur | 4 | Si | 12 | 0.02 | 0.01 | Female | Tibia | 2 | Si | 19 | 0.01 | 0.01 |
| Female | Femur | 4 | Zn | 12 | 0.07 | 0.08 | Female | Tibia | 2 | Zn | 19 | 0.04 | 0.01 |
| Female | Femur | 5 | Te | 18 | 1.60 | 0.33 | Female | Tibia | 3 | Te | 16 | 1.38 | 0.22 |
| Female | Femur | 5 | Ca | 18 | 0.04 | 0.04 | Female | Tibia | 3 | Ca | 16 | 0.02 | 0.01 |
| Female | Femur | 5 | Cl | 18 | 0.14 | 0.12 | Female | Tibia | 3 | Cl | 16 | 0.06 | 0.03 |
| Female | Femur | 5 | Cu | 18 | 0.04 | 0.02 | Female | Tibia | 3 | Cu | 16 | 0.30 | 0.51 |
| Female | Femur | 5 | F | 18 | 0.87 | 0.22 | Female | Tibia | 3 | F | 16 | 0.79 | 0.24 |
| Female | Femur | 5 | Fe | 18 | 0.03 | 0.01 | Female | Tibia | 3 | Fe | 16 | 0.03 | 0.01 |
| Female | Femur | 5 | K | 18 | 0.05 | 0.05 | Female | Tibia | 3 | K | 16 | 0.02 | 0.01 |
| Female | Femur | 5 | Mg | 18 | 0.07 | 0.04 | Female | Tibia | 3 | Mg | 16 | 0.04 | 0.02 |
| Female | Femur | 5 | Mn | 18 | 0.03 | 0.02 | Female | Tibia | 3 | Mn | 16 | 0.03 | 0.02 |
| Female | Femur | 5 | Na | 18 | 0.00 | 0.00 | Female | Tibia | 3 | Na | 16 | 0.00 | 0.01 |
| Female | Femur | 5 | P+Pt | 18 | 0.23 | 0.19 | Female | Tibia | 3 | P+Pt | 16 | 0.24 | 0.07 |
| Female | Femur | 5 | S | 18 | 0.04 | 0.03 | Female | Tibia | 3 | S | 16 | 0.03 | 0.01 |
| Female | Femur | 5 | Si | 18 | 0.03 | 0.07 | Female | Tibia | 3 | Si | 16 | 0.01 | 0.01 |
| Female | Femur | 5 | Zn | 18 | 0.03 | 0.01 | Female | Tibia | 3 | Zn | 16 | 0.05 | 0.07 |
| Female | Tibia | 1 | Te | 15 | 1.42 | 0.15 | Female | Spine | ds3 | Te | 8 | 1.62 | 0.20 |
| Female | Tibia | 1 | Ca | 15 | 0.02 | 0.01 | Female | Spine | ds3 | Ca | 8 | 0.10 | 0.08 |
| Female | Tibia | 1 | Cl | 15 | 0.01 | 0.01 | Female | Spine | ds3 | Cl | 8 | 0.08 | 0.03 |
| Female | Tibia | 1 | Cu | 15 | 0.04 | 0.02 | Female | Spine | ds3 | Cu | 8 | 0.06 | 0.02 |
| Female | Tibia | 1 | F | 15 | 0.95 | 0.07 | Female | Spine | ds3 | F | 8 | 0.90 | 0.26 |
| Female | Tibia | 1 | Fe | 15 | 0.02 | 0.01 | Female | Spine | ds3 | Fe | 8 | 0.04 | 0.01 |
| Female | Tibia | 1 | K | 15 | 0.01 | 0.01 | Female | Spine | ds3 | K | 8 | 0.02 | 0.01 |
| Female | Tibia | 1 | Mg | 15 | 0.08 | 0.02 | Female | Spine | ds3 | Mg | 8 | 0.03 | 0.02 |
| Female | Tibia | 1 | Mn | 15 | 0.02 | 0.01 | Female | Spine | ds3 | Mn | 8 | 0.03 | 0.01 |
| Female | Tibia | 1 | Na | 15 | 0.00 | 0.00 | Female | Spine | ds3 | Na | 8 | 0.00 | 0.00 |
| Female | Tibia | 1 | P+Pt | 15 | 0.18 | 0.03 | Female | Spine | ds3 | P+Pt | 8 | 0.27 | 0.08 |
| Female | Tibia | 1 | S | 15 | 0.01 | 0.01 | Female | Spine | ds3 | S | 8 | 0.03 | 0.02 |
| Female | Tibia | 1 | Si | 15 | 0.01 | 0.01 | Female | Spine | ds3 | Si | 8 | 0.01 | 0.01 |
| Female | Tibia | 1 | Zn | 15 | 0.04 | 0.01 | Female | Spine | ds3 | Zn | 8 | 0.06 | 0.02 |
| Female | Tibia | 2 | Te | 16 | 1.44 | 0.31 | Female | Spine | ds4 | Te | 14 | 1.14 | 0.18 |
| Female | Tibia | 2 | Ca | 16 | 0.02 | 0.01 | Female | Spine | ds4 | Ca | 14 | 0.02 | 0.01 |
| Female | Tibia | 2 | Cl | 16 | 0.03 | 0.01 | Female | Spine | ds4 | Cl | 14 | 0.08 | 0.06 |
| Female | Tibia | 2 | Cu | 16 | 0.07 | 0.13 | Female | Spine | ds4 | Cu | 14 | 0.04 | 0.01 |
| Female | Tibia | 2 | F | 16 | 0.87 | 0.12 | Female | Spine | ds4 | F | 14 | 0.65 | 0.20 |
| Female | Tibia | 2 | Fe | 16 | 0.03 | 0.01 | Female | Spine | ds4 | Fe | 14 | 0.03 | 0.01 |
| Female | Tibia | 2 | K | 16 | 0.02 | 0.01 | Female | Spine | ds4 | K | 14 | 0.03 | 0.01 |
| Female | Tibia | 2 | Mg | 16 | 0.09 | 0.03 | Female | Spine | ds4 | Mg | 14 | 0.03 | 0.02 |
| Female | Tibia | 2 | Mn | 16 | 0.02 | 0.01 | Female | Spine | ds4 | Mn | 14 | 0.03 | 0.01 |
| Female | Tibia | 2 | Na | 16 | 0.00 | 0.00 | Female | Spine | ds4 | Na | 14 | 0.00 | 0.00 |
| Female | Tibia | 2 | P+Pt | 16 | 0.19 | 0.04 | Female | Spine | ds4 | P+Pt | 14 | 0.17 | 0.04 |
| Female | Tibia | 2 | S | 16 | 0.02 | 0.01 | Female | Spine | ds4 | S | 14 | 0.03 | 0.01 |
| Female | Tibia | 2 | Si | 16 | 0.02 | 0.00 | Female | Spine | ds4 | Si | 14 | 0.01 | 0.01 |
| Female | Tibia | 2 | Zn | 16 | 0.06 | 0.08 | Female | Spine | ds4 | Zn | 14 | 0.03 | 0.01 |
| Female | Tibia | 3 | Te | 15 | 1.49 | 0.16 | Female | Spine | pvfs | Te | 23 | 1.42 | 0.28 |
| Female | Tibia | 3 | Ca | 15 | 0.09 | 0.04 | Female | Spine | pvfs | Ca | 23 | 0.02 | 0.02 |
| Female | Tibia | 3 | Cl | 15 | 0.03 | 0.02 | Female | Spine | pvfs | Cl | 23 | 0.04 | 0.04 |
| Female | Tibia | 3 | Cu | 15 | 0.04 | 0.03 | Female | Spine | pvfs | Cu | 23 | 0.04 | 0.02 |
| Female | Tibia | 3 | F | 15 | 0.90 | 0.14 | Female | Spine | pvfs | F | 23 | 0.82 | 0.34 |
| Female | Tibia | 3 | Fe | 15 | 0.03 | 0.01 | Female | Spine | pvfs | Fe | 23 | 0.03 | 0.01 |
| Female | Tibia | 3 | K | 15 | 0.02 | 0.00 | Female | Spine | pvfs | K | 23 | 0.02 | 0.02 |
| Female | Tibia | 3 | Mg | 15 | 0.06 | 0.03 | Female | Spine | pvfs | Mg | 23 | 0.07 | 0.04 |
| Female | Tibia | 3 | Mn | 15 | 0.02 | 0.01 | Female | Spine | pvfs | Mn | 23 | 0.03 | 0.02 |
| Female | Tibia | 3 | Na | 15 | 0.00 | 0.00 | Female | Spine | pvfs | Na | 23 | 0.00 | 0.00 |
| Female | Tibia | 3 | P+Pt | 15 | 0.21 | 0.05 | Female | Spine | pvfs | P+Pt | 23 | 0.24 | 0.08 |
| Female | Tibia | 3 | S | 15 | 0.02 | 0.01 | Female | Spine | pvfs | S | 23 | 0.02 | 0.01 |
| Female | Tibia | 3 | Si | 15 | 0.02 | 0.01 | Female | Spine | pvfs | Si | 23 | 0.01 | 0.01 |
| Female | Tibia | 3 | Zn | 15 | 0.04 | 0.02 | Female | Spine | pvfs | Zn | 23 | 0.04 | 0.02 |
| Female | Spine | ds1 | Te | 2 | 0.96 | 0.25 | Female | Spine | l avfs | Te | 33 | 1.54 | 0.42 |
| Female | Spine | ds1 | Ca | 2 | 0.04 | 0.02 | Female | Spine | l avfs | Ca | 33 | 0.02 | 0.02 |
| Female | Spine | ds1 | Cl | 2 | 0.08 | 0.02 | Female | Spine | l avfs | Cl | 33 | 0.06 | 0.07 |
| Female | Spine | ds1 | Cu | 2 | 0.03 | 0.01 | Female | Spine | l avfs | Cu | 33 | 0.04 | 0.02 |
| Female | Spine | ds1 | F | 2 | 0.52 | 0.22 | Female | Spine | l avfs | F | 33 | 0.91 | 0.18 |
| Female | Spine | ds1 | Fe | 2 | 0.03 | 0.01 | Female | Spine | l avfs | Fe | 33 | 0.03 | 0.01 |
| Female | Spine | ds1 | K | 2 | 0.03 | 0.00 | Female | Spine | l avfs | K | 33 | 0.03 | 0.04 |
| Female | Spine | ds1 | Mg | 2 | 0.00 | 0.00 | Female | Spine | l avfs | Mg | 33 | 0.09 | 0.05 |
| Female | Spine | ds1 | Mn | 2 | 0.00 | 0.00 | Female | Spine | l avfs | Mn | 33 | 0.02 | 0.01 |
| Female | Spine | ds1 | Na | 2 | 0.00 | 0.00 | Female | Spine | l avfs | Na | 33 | 0.00 | 0.00 |
| Female | Spine | ds1 | P+Pt | 2 | 0.17 | 0.06 | Female | Spine | l avfs | P+Pt | 33 | 0.21 | 0.11 |
| Female | Spine | ds1 | S | 2 | 0.02 | 0.01 | Female | Spine | l avfs | S | 33 | 0.03 | 0.03 |
| Female | Spine | ds1 | Si | 2 | 0.01 | 0.00 | Female | Spine | l avfs | Si | 33 | 0.02 | 0.01 |
| Female | Spine | ds1 | Zn | 2 | 0.03 | 0.01 | Female | Spine | l avfs | Zn | 33 | 0.03 | 0.02 |
| Female | Spine | ds2 | Te | 11 | 1.35 | 0.34 | Female | Spine | s avfs | Te | 24 | 1.43 | 0.27 |
| Female | Spine | ds2 | Ca | 11 | 0.02 | 0.01 | Female | Spine | s avfs | Ca | 24 | 0.02 | 0.02 |
| Female | Spine | ds2 | Cl | 11 | 0.04 | 0.05 | Female | Spine | s avfs | Cl | 24 | 0.06 | 0.07 |
| Female | Spine | ds2 | Cu | 11 | 0.05 | 0.03 | Female | Spine | s avfs | Cu | 24 | 0.04 | 0.02 |
| Female | Spine | ds2 | F | 11 | 0.78 | 0.30 | Female | Spine | s avfs | F | 24 | 0.84 | 0.21 |
| Female | Spine | ds2 | Fe | 11 | 0.03 | 0.01 | Female | Spine | s avfs | Fe | 24 | 0.03 | 0.01 |
| Female | Spine | ds2 | K | 11 | 0.02 | 0.01 | Female | Spine | s avfs | K | 24 | 0.03 | 0.04 |
| Female | Spine | ds2 | Mg | 11 | 0.05 | 0.03 | Female | Spine | s avfs | Mg | 24 | 0.08 | 0.04 |
| Female | Spine | ds2 | Mn | 11 | 0.03 | 0.01 | Female | Spine | s avfs | Mn | 24 | 0.02 | 0.01 |
| Female | Spine | ds2 | Na | 11 | 0.00 | 0.01 | Female | Spine | s avfs | Na | 24 | 0.00 | 0.01 |
| Female | Spine | ds2 | P+Pt | 11 | 0.24 | 0.11 | Female | Spine | s avfs | P+Pt | 24 | 0.20 | 0.07 |
| Female | Spine | ds2 | S | 11 | 0.02 | 0.01 | Female | Spine | s avfs | S | 24 | 0.03 | 0.04 |
| Female | Spine | ds2 | Si | 11 | 0.01 | 0.01 | Female | Spine | s avfs | Si | 24 | 0.02 | 0.01 |
| Female | Spine | ds2 | Zn | 11 | 0.05 | 0.03 | Female | Spine | s avfs | Zn | 24 | 0.04 | 0.02 |
| Female | Spine | ds3 | Te | 17 | 1.15 | 0.33 | Female | Spine | avts | Te | 65 | 1.57 | 0.49 |
| Female | Spine | ds3 | Ca | 17 | 0.02 | 0.01 | Female | Spine | avts | Ca | 65 | 0.02 | 0.01 |
| Female | Spine | ds3 | Cl | 17 | 0.04 | 0.02 | Female | Spine | avts | Cl | 65 | 0.04 | 0.03 |
| Female | Spine | ds3 | Cu | 17 | 0.03 | 0.02 | Female | Spine | avts | Cu | 65 | 0.04 | 0.01 |
| Female | Spine | ds3 | F | 17 | 0.70 | 0.28 | Female | Spine | avts | F | 65 | 0.99 | 0.28 |
| Female | Spine | ds3 | Fe | 17 | 0.02 | 0.01 | Female | Spine | avts | Fe | 65 | 0.03 | 0.01 |
| Female | Spine | ds3 | K | 17 | 0.02 | 0.01 | Female | Spine | avts | K | 65 | 0.02 | 0.01 |
| Female | Spine | ds3 | Mg | 17 | 0.06 | 0.04 | Female | Spine | avts | Mg | 65 | 0.07 | 0.03 |
| Female | Spine | ds3 | Mn | 17 | 0.02 | 0.01 | Female | Spine | avts | Mn | 65 | 0.02 | 0.01 |
| Female | Spine | ds3 | Na | 17 | 0.00 | 0.00 | Female | Spine | avts | Na | 65 | 0.00 | 0.00 |
| Female | Spine | ds3 | P+Pt | 17 | 0.16 | 0.05 | Female | Spine | avts | P+Pt | 65 | 0.23 | 0.19 |
| Female | Spine | ds3 | S | 17 | 0.02 | 0.01 | Female | Spine | avts | S | 65 | 0.03 | 0.02 |
| Female | Spine | ds3 | Si | 17 | 0.02 | 0.01 | Female | Spine | avts | Si | 65 | 0.01 | 0.01 |
| Female | Spine | ds3 | Zn | 17 | 0.03 | 0.01 | Female | Spine | avts | Zn | 65 | 0.04 | 0.01 |
| Female | Spine | ds4 | Te | 14 | 1.56 | 0.26 | Female | Spine | ts | Te | 27 | 1.24 | 0.25 |
| Female | Spine | ds4 | Ca | 14 | 0.03 | 0.02 | Female | Spine | ts | Ca | 27 | 0.02 | 0.01 |
| Female | Spine | ds4 | Cl | 14 | 0.07 | 0.04 | Female | Spine | ts | Cl | 27 | 0.04 | 0.03 |
| Female | Spine | ds4 | Cu | 14 | 0.04 | 0.01 | Female | Spine | ts | Cu | 27 | 0.03 | 0.01 |
| Female | Spine | ds4 | F | 14 | 0.82 | 0.17 | Female | Spine | ts | F | 27 | 0.77 | 0.24 |
| Female | Spine | ds4 | Fe | 14 | 0.03 | 0.01 | Female | Spine | ts | Fe | 27 | 0.03 | 0.01 |
| Female | Spine | ds4 | K | 14 | 0.08 | 0.06 | Female | Spine | ts | K | 27 | 0.02 | 0.01 |
| Female | Spine | ds4 | Mg | 14 | 0.09 | 0.04 | Female | Spine | ts | Mg | 27 | 0.07 | 0.04 |
| Female | Spine | ds4 | Mn | 14 | 0.03 | 0.01 | Female | Spine | ts | Mn | 27 | 0.02 | 0.01 |
| Female | Spine | ds4 | Na | 14 | 0.00 | 0.01 | Female | Spine | ts | Na | 27 | 0.00 | 0.01 |
| Female | Spine | ds4 | P+Pt | 14 | 0.24 | 0.06 | Female | Spine | ts | P+Pt | 27 | 0.17 | 0.04 |
| Female | Spine | ds4 | S | 14 | 0.04 | 0.02 | Female | Spine | ts | S | 27 | 0.03 | 0.01 |
| Female | Spine | ds4 | Si | 14 | 0.02 | 0.00 | Female | Spine | ts | Si | 27 | 0.02 | 0.01 |
| Female | Spine | ds4 | Zn | 14 | 0.04 | 0.01 | Female | Spine | ts | Zn | 27 | 0.03 | 0.01 |
| Female | Spine | pvfs | Te | 17 | 1.51 | 0.12 |  |  |  |  |  |  |  |
| Female | Spine | pvfs | Ca | 17 | 0.02 | 0.01 |  |  |  |  |  |  |  |
| Female | Spine | pvfs | Cl | 17 | 0.03 | 0.02 |  |  |  |  |  |  |  |
| Female | Spine | pvfs | Cu | 17 | 0.04 | 0.01 |  |  |  |  |  |  |  |
| Female | Spine | pvfs | F | 17 | 0.97 | 0.06 |  |  |  |  |  |  |  |
| Female | Spine | pvfs | Fe | 17 | 0.03 | 0.01 |  |  |  |  |  |  |  |
| Female | Spine | pvfs | K | 17 | 0.02 | 0.01 |  |  |  |  |  |  |  |
| Female | Spine | pvfs | Mg | 17 | 0.10 | 0.02 |  |  |  |  |  |  |  |
| Female | Spine | pvfs | Mn | 17 | 0.02 | 0.01 |  |  |  |  |  |  |  |
| Female | Spine | pvfs | Na | 17 | 0.00 | 0.00 |  |  |  |  |  |  |  |
| Female | Spine | pvfs | P+Pt | 17 | 0.20 | 0.03 |  |  |  |  |  |  |  |
| Female | Spine | pvfs | S | 17 | 0.01 | 0.01 |  |  |  |  |  |  |  |
| Female | Spine | pvfs | Si | 17 | 0.04 | 0.07 |  |  |  |  |  |  |  |
| Female | Spine | pvfs | Zn | 17 | 0.04 | 0.01 |  |  |  |  |  |  |  |
| Female | Spine | nt avfs | Te | 43 | 1.70 | 0.91 |  |  |  |  |  |  |  |
| Female | Spine | nt avfs | Ca | 43 | 0.02 | 0.01 |  |  |  |  |  |  |  |
| Female | Spine | nt avfs | Cl | 43 | 0.12 | 0.08 |  |  |  |  |  |  |  |
| Female | Spine | nt avfs | Cu | 43 | 0.04 | 0.02 |  |  |  |  |  |  |  |
| Female | Spine | nt avfs | F | 43 | 1.03 | 0.64 |  |  |  |  |  |  |  |
| Female | Spine | nt avfs | Fe | 43 | 0.03 | 0.02 |  |  |  |  |  |  |  |
| Female | Spine | nt avfs | K | 43 | 0.03 | 0.02 |  |  |  |  |  |  |  |
| Female | Spine | nt avfs | Mg | 43 | 0.08 | 0.05 |  |  |  |  |  |  |  |
| Female | Spine | nt avfs | Mn | 43 | 0.02 | 0.01 |  |  |  |  |  |  |  |
| Female | Spine | nt avfs | Na | 43 | 0.00 | 0.00 |  |  |  |  |  |  |  |
| Female | Spine | nt avfs | P+Pt | 43 | 0.25 | 0.24 |  |  |  |  |  |  |  |
| Female | Spine | nt avfs | S | 43 | 0.02 | 0.01 |  |  |  |  |  |  |  |
| Female | Spine | nt avfs | Si | 43 | 0.01 | 0.01 |  |  |  |  |  |  |  |
| Female | Spine | nt avfs | Zn | 43 | 0.04 | 0.03 |  |  |  |  |  |  |  |
| Female | Spine | t avfs | Te | 22 | 1.46 | 0.51 |  |  |  |  |  |  |  |
| Female | Spine | t avfs | Ca | 22 | 0.02 | 0.03 |  |  |  |  |  |  |  |
| Female | Spine | t avfs | Cl | 22 | 0.09 | 0.14 |  |  |  |  |  |  |  |
| Female | Spine | t avfs | Cu | 22 | 0.03 | 0.01 |  |  |  |  |  |  |  |
| Female | Spine | t avfs | F | 22 | 0.82 | 0.15 |  |  |  |  |  |  |  |
| Female | Spine | t avfs | Fe | 22 | 0.03 | 0.01 |  |  |  |  |  |  |  |
| Female | Spine | t avfs | K | 22 | 0.03 | 0.03 |  |  |  |  |  |  |  |
| Female | Spine | t avfs | Mg | 22 | 0.07 | 0.03 |  |  |  |  |  |  |  |
| Female | Spine | t avfs | Mn | 22 | 0.02 | 0.01 |  |  |  |  |  |  |  |
| Female | Spine | t avfs | Na | 22 | 0.00 | 0.00 |  |  |  |  |  |  |  |
| Female | Spine | t avfs | P+Pt | 22 | 0.17 | 0.06 |  |  |  |  |  |  |  |
| Female | Spine | t avfs | S | 22 | 0.02 | 0.02 |  |  |  |  |  |  |  |
| Female | Spine | t avfs | Si | 22 | 0.11 | 0.42 |  |  |  |  |  |  |  |
| Female | Spine | t avfs | Zn | 22 | 0.03 | 0.01 |  |  |  |  |  |  |  |
| Female | Spine | avts | Te | 32 | 1.47 | 0.25 |  |  |  |  |  |  |  |
| Female | Spine | avts | Ca | 32 | 0.02 | 0.01 |  |  |  |  |  |  |  |
| Female | Spine | avts | Cl | 32 | 0.07 | 0.04 |  |  |  |  |  |  |  |
| Female | Spine | avts | Cu | 32 | 0.04 | 0.02 |  |  |  |  |  |  |  |
| Female | Spine | avts | F | 32 | 0.91 | 0.19 |  |  |  |  |  |  |  |
| Female | Spine | avts | Fe | 32 | 0.03 | 0.01 |  |  |  |  |  |  |  |
| Female | Spine | avts | K | 32 | 0.04 | 0.02 |  |  |  |  |  |  |  |
| Female | Spine | avts | Mg | 32 | 0.08 | 0.04 |  |  |  |  |  |  |  |
| Female | Spine | avts | Mn | 32 | 0.02 | 0.01 |  |  |  |  |  |  |  |
| Female | Spine | avts | Na | 32 | 0.00 | 0.00 |  |  |  |  |  |  |  |
| Female | Spine | avts | P+Pt | 32 | 0.20 | 0.05 |  |  |  |  |  |  |  |
| Female | Spine | avts | S | 32 | 0.02 | 0.01 |  |  |  |  |  |  |  |
| Female | Spine | avts | Si | 32 | 0.02 | 0.01 |  |  |  |  |  |  |  |
| Female | Spine | avts | Zn | 32 | 0.03 | 0.02 |  |  |  |  |  |  |  |
| Female | Spine | ts | Te | 19 | 1.28 | 0.21 |  |  |  |  |  |  |  |
| Female | Spine | ts | Ca | 19 | 0.02 | 0.01 |  |  |  |  |  |  |  |
| Female | Spine | ts | Cl | 19 | 0.04 | 0.03 |  |  |  |  |  |  |  |
| Female | Spine | ts | Cu | 19 | 0.04 | 0.01 |  |  |  |  |  |  |  |
| Female | Spine | ts | F | 19 | 0.83 | 0.18 |  |  |  |  |  |  |  |
| Female | Spine | ts | Fe | 19 | 0.03 | 0.01 |  |  |  |  |  |  |  |
| Female | Spine | ts | K | 19 | 0.02 | 0.01 |  |  |  |  |  |  |  |
| Female | Spine | ts | Mg | 19 | 0.06 | 0.03 |  |  |  |  |  |  |  |
| Female | Spine | ts | Mn | 19 | 0.03 | 0.01 |  |  |  |  |  |  |  |
| Female | Spine | ts | Na | 19 | 0.00 | 0.00 |  |  |  |  |  |  |  |
| Female | Spine | ts | P+Pt | 19 | 0.16 | 0.05 |  |  |  |  |  |  |  |
| Female | Spine | ts | S | 19 | 0.01 | 0.01 |  |  |  |  |  |  |  |
| Female | Spine | ts | Si | 19 | 0.01 | 0.01 |  |  |  |  |  |  |  |
| Female | Spine | ts | Zn | 19 | 0.03 | 0.01 |  |  |  |  |  |  |  |

**Supplementary Table 8. Results of the EDX analysis, given in atomic %, for the different regions of the tibial claw from the male *Sphodromantis lineola*. We tested one male. Abbreviations: Ca, calcium; Cl, chlorine; Cu, copper; F, fluorine; Fe, iron; K, potassium; Mg, magnesium; Mn, manganese; N, number of EDX measurements; Na, sodium; P+Pt, sum of phosphorus and platinum; S, sulphur; SD, standard deviation; Si, silicon;** Te, trace elements, sum of Ca, Cl, Cu, F, Fe, K, Mg, Mn, Na, P+Pt, S, Si, and Zn**; Zn, zinc.**

| **Element** | **Region** | **N** | **Mean** | **SD** |
| --- | --- | --- | --- | --- |
| Te | 1 | 6 | 1.57 | 0.12 |
| Te | 2 | 8 | 1.48 | 0.22 |
| Te | 3 | 4 | 1.44 | 0.11 |
| Te | 4 | 7 | 1.30 | 0.15 |
| Ca | 1 | 6 | 0.01 | 0.01 |
| Ca | 2 | 8 | 0.02 | 0.01 |
| Ca | 3 | 4 | 0.02 | 0.01 |
| Ca | 4 | 7 | 0.02 | 0.01 |
| Cl | 1 | 6 | 0.12 | 0.03 |
| Cl | 2 | 8 | 0.06 | 0.02 |
| Cl | 3 | 4 | 0.03 | 0.01 |
| Cl | 4 | 7 | 0.02 | 0.01 |
| Cu | 1 | 6 | 0.03 | 0.01 |
| Cu | 2 | 8 | 0.04 | 0.02 |
| Cu | 3 | 4 | 0.04 | 0.01 |
| Cu | 4 | 7 | 0.05 | 0.03 |
| F | 1 | 6 | 0.99 | 0.12 |
| F | 2 | 8 | 0.89 | 0.22 |
| F | 3 | 4 | 0.88 | 0.11 |
| F | 4 | 7 | 0.83 | 0.14 |
| Fe | 1 | 6 | 0.03 | 0.01 |
| Fe | 2 | 8 | 0.03 | 0.01 |
| Fe | 3 | 4 | 0.03 | 0.01 |
| Fe | 4 | 7 | 0.03 | 0.01 |
| K | 1 | 6 | 0.02 | 0.01 |
| K | 2 | 8 | 0.02 | 0.01 |
| K | 3 | 4 | 0.03 | 0.01 |
| K | 4 | 7 | 0.02 | 0.01 |
| Mg | 1 | 6 | 0.08 | 0.03 |
| Mg | 2 | 8 | 0.08 | 0.04 |
| Mg | 3 | 4 | 0.09 | 0.04 |
| Mg | 4 | 7 | 0.05 | 0.03 |
| Mn | 1 | 6 | 0.02 | 0.01 |
| Mn | 2 | 8 | 0.03 | 0.01 |
| Mn | 3 | 4 | 0.03 | 0.01 |
| Mn | 4 | 7 | 0.02 | 0.01 |
| Na | 1 | 6 | 0.00 | 0.00 |
| Na | 2 | 8 | 0.01 | 0.02 |
| Na | 3 | 4 | 0.00 | 0.00 |
| Na | 4 | 7 | 0.00 | 0.00 |
| P+Pt | 1 | 6 | 0.23 | 0.05 |
| P+Pt | 2 | 8 | 0.23 | 0.07 |
| P+Pt | 3 | 4 | 0.22 | 0.01 |
| P+Pt | 4 | 7 | 0.19 | 0.09 |
| S | 1 | 6 | 0.01 | 0.01 |
| S | 2 | 8 | 0.02 | 0.00 |
| S | 3 | 4 | 0.02 | 0.01 |
| S | 4 | 7 | 0.02 | 0.01 |
| Si | 1 | 6 | 0.01 | 0.00 |
| Si | 2 | 8 | 0.02 | 0.03 |
| Si | 3 | 4 | 0.01 | 0.01 |
| Si | 4 | 7 | 0.01 | 0.00 |
| Zn | 1 | 6 | 0.03 | 0.01 |
| Zn | 2 | 8 | 0.04 | 0.01 |
| Zn | 3 | 4 | 0.04 | 0.01 |
| Zn | 4 | 7 | 0.05 | 0.02 |

**Supplementary Table 9. Results of nanoindentation, the hardness and Young’s modulus, both given in GPa, for the four regions of the tibial spur.** **We tested one specimen. Abbreviations: N, number of nanoindentation measurements.**

| **Structure** | **N** | **Hardness** | | **Young’s modulus** | |
| --- | --- | --- | --- | --- | --- |
|  |  | **Mean** | **SD** | **Mean** | **SD** |
| *S. lineola* Tibial spur Region 1 | 7 | 0.63 | 0.10 | 8.64 | 0.78 |
| *S. lineola* Tibial spur Region 2 | 4 | 0.57 | 0.06 | 7.97 | 0.02 |
| *S. lineola* Tibial spur Region 3 | 8 | 0.46 | 0.04 | 6.64 | 0.53 |
| *S. lineola* Tibial spur Region 4 | 6 | 0.37 | 0.02 | 4.96 | 0.44 |

**Supplementary Table 10. Pairwise comparison between the different regions of the tibial spur tested by Wilcoxon method. Colours of the p-values: black, not significant; yellow, highly significant. We tested one specimen.**

| **Structure 1** | **- Structure 2** | **Hardness**  **p-value** | **Young’s modulus**  **p-value** |
| --- | --- | --- | --- |
| *S. lineola* Tibial spur Region 1 | *S. lineola* Tibial spur Region 3 | 0.0013* | 0.0014* |
| *S. lineola* Tibial spur Region 3 | *S. lineola* Tibial spur Region 4 | 0.0023* | 0.0024* |
| *S. lineola* Tibial spur Region 1 | *S. lineola* Tibial spur Region 4 | 0.0029* | 0.0034* |
| *S. lineola* Tibial spur Region 2 | *S. lineola* Tibial spur Region 3 | 0.0128* | 0.0084* |
| *S. lineola* Tibial spur Region 2 | *S. lineola* Tibial spur Region 4 | 0.0136* | 0.0142* |
| *S. lineola* Tibial spur Region 1 | *S. lineola* Tibial spur Region 2 | 0.1362 | 0.0138* |

**Supplementary Table 11. Results of nanoindentation, the hardness and Young’s modulus, both given in GPa, for the tested regions of the tibia and femur.** **For each species, we tested one male and one female. Abbreviations: N, number of nanoindentation measurements.** Abbreviations: avts, anteroventral tibial spines; l avfs, long anteroventral femoral spines; nt avfs, non-tiltable anteroventral femoral spines; s avfs, short anteroventral femoral spines; t avfs, tiltable anteroventral femoral spines.

| **Structure** | **N** | **Hardness** | | **Young’s modulus** | |
| --- | --- | --- | --- | --- | --- |
|  |  | **Mean** | **SD** | **Mean** | **SD** |
| *G. gongylodes* Femur Region 5 | 58 | 0.04 | 0.01 | 1.86 | 0.17 |
| *G. gongylodes* Tibia Region 2 | 34 | 0.05 | 0.02 | 2.29 | 0.08 |
| *S. lineola* Tibia Region 2 | 33 | 0.06 | 0.01 | 2.58 | 0.09 |
| *G. gongylodes* Femur Region 4 | 44 | 0.08 | 0.01 | 2.96 | 0.13 |
| *S. lineola* Femur Region 5 | 41 | 0.10 | 0.02 | 3.33 | 0.09 |
| *S. lineola* Femur Region 4 | 39 | 0.13 | 0.03 | 3.57 | 0.06 |
| *G. gongylodes* l avfs | 52 | 0.16 | 0.05 | 3.79 | 0.07 |
| *G. gongylodes* s avfs | 57 | 0.20 | 0.06 | 4.05 | 0.08 |
| *G. gongylodes* avts | 99 | 0.22 | 0.08 | 4.50 | 0.19 |
| *S. lineola* nt avfs | 62 | 0.24 | 0.06 | 5.11 | 0.15 |
| *S. lineola* t avfs | 37 | 0.29 | 0.09 | 5.56 | 0.11 |
| *S. lineola* avts | 55 | 0.34 | 0.09 | 6.27 | 0.40 |

**Supplementary Table 12. For hardness values - pairwise comparison between the different regions of the tibia and femur tested by Wilcoxon method. Colours of the p-values: black, not significant; yellow, highly significant. For each species, we tested one male and one female.** Abbreviations: avts, anteroventral tibial spines; l avfs, long anteroventral femoral spines; nt avfs, non-tiltable anteroventral femoral spines; s avfs, short anteroventral femoral spines; t avfs, tiltable anteroventral femoral spines.

| **Structure 1** | **- Structure 2** | **p-value** |
| --- | --- | --- |
| *S. lineola* nt avfs | *G. gongylodes* Femur 5 | <.0001* |
| *S. lineola* avts | *G. gongylodes* Femur 5 | <.0001* |
| *S. lineola* nt avfs | *G. gongylodes* Femur 4 | <.0001* |
| *S. lineola* avts | *G. gongylodes* avts | <.0001* |
| *S. lineola* avts | *G. gongylodes* l avfs | <.0001* |
| *S. lineola* avts | *G. gongylodes* Femur 4 | <.0001* |
| *S. lineola* Femur Region 5 | *G. gongylodes* Femur 5 | <.0001* |
| *S. lineola* Femur Region 4 | *G. gongylodes* Femur Region 5 | <.0001* |
| *S. lineola* nt avfs | *G. gongylodes* Tibia Region 2 | <.0001* |
| *S. lineola* t avfs | *G. gongylodes* Femur Region 5 | <.0001* |
| *S. lineola* avts | *G. gongylodes* s avfs | <.0001* |
| *S. lineola* avts | *G. gongylodes* Tibia Region 2 | <.0001* |
| *S. lineola* nt avfs | *G. gongylodes* l avfs | <.0001* |
| *S. lineola* t avfs | *G. gongylodes* Femur Region 4 | <.0001* |
| *S. lineola* Femur Region 4 | *G. gongylodes* Femur Region 4 | <.0001* |
| *S. lineola* avts | *S. lineola* nt avfs | <.0001* |
| *S. lineola* Femur Region 4 | *G. gongylodes* Tibia Region 2 | <.0001* |
| *S. lineola* Femur Region 5 | *G. gongylodes* Tibia Region 2 | <.0001* |
| *S. lineola* t avfs | *G. gongylodes* Tibia Region 2 | <.0001* |
| *S. lineola* t avfs | *G. gongylodes* l avfs | <.0001* |
| *S. lineola* Tibia Region 2 | *G. gongylodes* Femur Region 5 | <.0001* |
| *G. gongylodes* avts | *G. gongylodes* l avfs | <.0001* |
| *S. lineola* t avfs | *G. gongylodes* avts | <.0001* |
| *S. lineola* Femur Region 5 | *G. gongylodes* Femur Region 4 | <.0001* |
| *S. lineola* t avfs | *G. gongylodes* s avfs | <.0001* |
| *S. lineola* nt avfs | *G. gongylodes* s avfs | <.0001* |
| *G. gongylodes* s avfs | *G. gongylodes* l avfs | 0.0002* |
| *S. lineola* t avfs | *S. lineola* nt avfs | 0.0003* |
| *S. lineola* nt avfs | *G. gongylodes* avts | 0.0241* |
| *G. gongylodes* Tibia Region 2 | *G. gongylodes* Femur Region 5 | 0.0019* |
| *S. lineola* avts | *S. lineola* t avfs | 0.0277* |
| *S. lineola* Tibia Region 2 | *G. gongylodes* Tibia Region 2 | 0.0153* |
| *G. gongylodes* avts | *G. gongylodes* s avfs | 0.1703 |
| *S. lineola* Femur Region 4 | *G. gongylodes* l avfs | 0.0020* |
| *S. lineola* Femur Region 5 | *S. lineola* Femur Region 4 | <.0001* |
| *S. lineola* Tibia Region 2 | *G. gongylodes* Femur Region 4 | <.0001* |
| *G. gongylodes* Tibia Region 2 | *G. gongylodes* Femur Region 4 | <.0001* |
| *S. lineola* Femur Region 4 | *S. lineola* t avfs | <.0001* |
| *S. lineola* Tibia Region 2 | *S. lineola* Femur Region 5 | <.0001* |
| *S. lineola* Femur Region 5 | *G. gongylodes* l avfs | <.0001* |
| *S. lineola* Femur Region 4 | *G. gongylodes* s avfs | <.0001* |
| *S. lineola* Tibia Region 2 | *S. lineola* s avfs | <.0001* |
| *S. lineola* Tibia Region 2 | *S. lineola* Femur Region 4 | <.0001* |
| *S. lineola* Femur Region 5 | *S. lineola* s avfs | <.0001* |
| *S. lineola* Tibia Region 2 | *G. gongylodes* l avfs | <.0001* |
| *G. gongylodes* Tibia Region 2 | *G. gongylodes* l avfs | <.0001* |
| *S. lineola* Femur Region 5 | *G. gongylodes* s avfs | <.0001* |
| *S. lineola* Tibia Region 2 | *S. lineola* avts | <.0001* |
| *S. lineola* Tibia Region 2 | *G. gongylodes* s avfs | <.0001* |
| *S. lineola* Femur Region 4 | *S. lineola* nt avfs | <.0001* |
| *G. gongylodes* Tibia Region 2 | *G. gongylodes* s avfs | <.0001* |
| *S. lineola* Femur Region 4 | *G. gongylodes* avts | <.0001* |
| *S. lineola* Femur Region 4 | *S. lineola* avts | <.0001* |
| *G. gongylodes* Femur Region 4 | *G. gongylodes* l avfs | <.0001* |
| *S. lineola* Tibia Region 2 | *S. lineola* nt avfs | <.0001* |
| *S. lineola* Femur Region 5 | *S. lineola* avts | <.0001* |
| *G. gongylodes* Femur Region 5 | *G. gongylodes* Femur Region 4 | <.0001* |
| *S. lineola* Femur Region 5 | *S. lineola* nt avfs | <.0001* |
| *G. gongylodes* Femur Region 4 | *G. gongylodes* s avfs | <.0001* |
| *G. gongylodes* Femur Region 5 | *G. gongylodes* l avfs | <.0001* |
| *G. gongylodes* Femur Region 5 | *G. gongylodes* s avfs | <.0001* |
| *S. lineola* Femur Region 5 | *G. gongylodes* avts | <.0001* |
| *S. lineola* Tibia Region 2 | *G. gongylodes* avts | <.0001* |
| *G. gongylodes* Tibia Region 2 | *G. gongylodes* avts | <.0001* |
| *G. gongylodes* Femur Region 4 | *G. gongylodes* avts | <.0001* |
| *G. gongylodes* Femur Region 5 | *G. gongylodes* avts | <.0001* |

**Supplementary Table 13. For Young’s modulus values - pairwise comparison between the different regions of the tibia and femur tested by Wilcoxon method. Colours of the p-values: black, not significant; yellow, highly significant. For each species, we tested one male and one female.** Abbreviations: avts, anteroventral tibial spines; l avfs, long anteroventral femoral spines; nt avfs, non-tiltable anteroventral femoral spines; s avfs, short anteroventral femoral spines; t avfs, tiltable anteroventral femoral spines.

| **Structure 1** | **- Structure 2** | **p-value** |
| --- | --- | --- |
| *S. lineola* nt avfs | *G. gongylodes* avts | <.0001* |
| *G. gongylodes* avts | *G. gongylodes* s avfs | <.0001* |
| *S. lineola* avts | *G. gongylodes* avts | <.0001* |
| *G. gongylodes* avts | *G. gongylodes* l avfs | <.0001* |
| *S. lineola* t avfs | *G. gongylodes* avts | <.0001* |
| *S. lineola* nt avfs | *G. gongylodes* Femur Region 5 | <.0001* |
| *S. lineola* nt avfs | *G. gongylodes* s avfs | <.0001* |
| *S. lineola* avts | *S. lineola* nt avfs | <.0001* |
| *S. lineola* nt avfs | *G. gongylodes* l avfs | <.0001* |
| *S. lineola* avts | *G. gongylodes* Femur Region 5 | <.0001* |
| *S. lineola* avts | *G. gongylodes* s avfs | <.0001* |
| *G. gongylodes* s avfs | *G. gongylodes* l avfs | <.0001* |
| *S. lineola* avts | *G. gongylodes* l avfs | <.0001* |
| *S. lineola* nt avfs | *G. gongylodes* Femur Region 4 | <.0001* |
| *S. lineola* avts | *G. gongylodes* Femur Region 4 | <.0001* |
| *S. lineola* Femur Region 5 | *G. gongylodes* Femur Region 5 | <.0001* |
| *S. lineola* t avfs | *S. lineola* nt avfs | <.0001* |
| *S. lineola* Femur Region 4 | *G. gongylodes* Femur Region 5 | <.0001* |
| *S. lineola* nt avfs | *G. gongylodes* Tibia Region 2 | <.0001* |
| *S. lineola* t avfs | *G. gongylodes* Femur Region 5 | <.0001* |
| *S. lineola* t avfs | *G. gongylodes* s avfs | <.0001* |
| *S. lineola* avts | *S. lineola* t avfs | <.0001* |
| *G. gongylodes* Tibia Region 2 | *G. gongylodes* Femur Region 5 | <.0001* |
| *S. lineola* Tibia Region 2 | *G. gongylodes* Femur Region 5 | <.0001* |
| *S. lineola* t avfs | *G. gongylodes* l avfs | <.0001* |
| *S. lineola* avts | *G. gongylodes* Tibia Region 2 | <.0001* |
| *S. lineola* Femur Region 5 | *G. gongylodes* Femur Region 4 | <.0001* |
| *S. lineola* Femur Region 4 | *G. gongylodes* Femur Region 4 | <.0001* |
| *S. lineola* t avfs | *G. gongylodes* Femur Region 4 | <.0001* |
| *S. lineola* Femur Region 5 | *G. gongylodes* Tibia Region 2 | <.0001* |
| *S. lineola* Femur Region 4 | *G. gongylodes* Tibia Region 2 | <.0001* |
| *S. lineola* t avfs | *G. gongylodes* Tibia Region 2 | <.0001* |
| *S. lineola* Tibia Region 2 | *G. gongylodes* Tibia Region 2 | <.0001* |
| *S. lineola* Tibia Region 2 | *S. lineola* t avfs | <.0001* |
| *S. lineola* Tibia Region 2 | *S. lineola* Femur Region 4 | <.0001* |
| *S. lineola* Tibia Region 2 | *S. lineola* Femur Region 5 | <.0001* |
| *S. lineola* Femur Region 4 | *S. lineola* t avfs | <.0001* |
| *S. lineola* Tibia Region 2 | *G. gongylodes* Femur Region 4 | <.0001* |
| *G. gongylodes* Tibia Region 2 | *G. gongylodes* Femur Region 4 | <.0001* |
| *S. lineola* Femur Region 5 | *S. lineola* t avfs | <.0001* |
| *S. lineola* Femur Region 5 | *S. lineola* Femur Region 4 | <.0001* |
| *S. lineola* Tibia Region 2 | *G. gongylodes* l avfs | <.0001* |
| *G. gongylodes* Tibia Region 2 | *G. gongylodes* l avfs | <.0001* |
| *S. lineola* Tibia Region 2 | *S. lineola* avts | <.0001* |
| *S. lineola* Tibia Region 2 | *G. gongylodes* s avfs | <.0001* |
| *S. lineola* Femur Region 4 | *G. gongylodes* l avfs | <.0001* |
| *G. gongylodes* Tibia Region 2 | *G. gongylodes* s avfs | <.0001* |
| *S. lineola* Femur Region 5 | *G. gongylodes* l avfs | <.0001* |
| *S. lineola* Femur Region 4 | *S. lineola* avts | <.0001* |
| *S. lineola* Tibia Region 2 | *S. lineola* nt avfs | <.0001* |
| *S. lineola* Femur Region 4 | *G. gongylodes* s avfs | <.0001* |
| *S. lineola* Femur Region 5 | *S. lineola* avts | <.0001* |
| *G. gongylodes* Femur Region 4 | *G. gongylodes* l avfs | <.0001* |
| *S. lineola* Femur Region 5 | *G. gongylodes* s avfs | <.0001* |
| *S. lineola* Femur Region 4 | *S. lineola* nt avfs | <.0001* |
| *G. gongylodes* Femur Region 4 | *G. gongylodes* s avfs | <.0001* |
| *G. gongylodes* Femur Region 5 | *G. gongylodes* Femur Region 4 | <.0001* |
| *S. lineola* Femur Region 5 | *S. lineola* nt avfs | <.0001* |
| *G. gongylodes* Femur Region 5 | *G. gongylodes* l avfs | <.0001* |
| *G. gongylodes* Femur Region 5 | *G. gongylodes* s avfs | <.0001* |
| *S. lineola* Tibia Region 2 | *G. gongylodes* avts | <.0001* |
| *G. gongylodes* Tibia Region 2 | *G. gongylodes* avts | <.0001* |
| *S. lineola* Femur Region 4 | *G. gongylodes* avts | <.0001* |
| *S. lineola* Femur Region 5 | *G. gongylodes* avts | <.0001* |
| *G. gongylodes* Femur Region 4 | *G. gongylodes* avts | <.0001* |
| *G. gongylodes* Femur Region 5 | *G. gongylodes* avts | <.0001* |

**Supplementary Table 14. C**orrelation coefficients, estimated by row-wise method, between the discussed elements and the mechanical properties are shown. 1.00 = perfect correlation; 0.80–0.99 = very strong correlation; 0.60–0.79 = strong correlation; 0.40–0.59 = moderate correlation; 0.20–0.39 = weak correlation; 0.00–0.19 = very weak correlation. **For each species, we tested one male and one female.**

|  | **Ca** | **Cl** | **Cu** | **E** | **F** | **Fe** | **H** | **K** | **Mg** | **Mn** | **Na** | **P+Pt** | **S** | **Si** | **Zn** |
| --- | --- | --- | --- | --- | --- | --- | --- | --- | --- | --- | --- | --- | --- | --- | --- |
| **Ca** | 1.00 | 0.56 | 0.27 | -0.12 | -0.12 | 0.49 | -0.09 | 0.78 | 0.38 | 0.46 | 0.31 | 0.52 | 0.78 | -0.04 | 0.50 |
| **Cl** |  | 1.00 | 0.13 | 0.09 | -0.35 | 0.54 | 0.07 | 0.50 | -0.13 | 0.48 | 0.01 | 0.08 | 0.30 | -0.03 | 0.15 |
| **Cu** |  |  | 1.00 | -0.14 | -0.13 | 0.40 | -0.12 | 0.26 | 0.07 | 0.45 | 0.17 | 0.36 | 0.31 | 0.09 | 0.87 |
| **E** |  |  |  | 1.00 | -0.14 | -0.00 | 0.88 | -0.09 | -0.22 | 0.03 | -0.08 | -0.19 | -0.20 | 0.01 | -0.16 |
| **F** |  |  |  |  | 1.00 | -0.17 | -0.12 | -0.06 | 0.44 | -0.42 | 0.04 | 0.23 | -0.04 | -0.00 | -0.04 |
| **Fe** |  |  |  |  |  | 1.00 | 0.02 | 0.37 | -0.10 | 0.82 | 0.06 | 0.41 | 0.35 | -0.06 | 0.44 |
| **H** |  |  |  |  |  |  | 1.00 | -0.07 | -0.18 | 0.05 | -0.06 | -0.14 | -0.17 | -0.01 | -0.12 |
| **K** |  |  |  |  |  |  |  | 1.00 | 0.58 | 0.31 | 0.64 | 0.66 | 0.82 | -0.03 | 0.48 |
| **Mg** |  |  |  |  |  |  |  |  | 1.00 | -0.18 | 0.47 | 0.60 | 0.58 | 0.03 | 0.31 |
| **Mn** |  |  |  |  |  |  |  |  |  | 1.00 | 0.08 | 0.35 | 0.33 | -0.04 | 0.45 |
| **Na** |  |  |  |  |  |  |  |  |  |  | 1.00 | 0.56 | 0.45 | -0.01 | 0.36 |
| **P+Pt** |  |  |  |  |  |  |  |  |  |  |  | 1.00 | 0.68 | -0.05 | 0.56 |
| **S** |  |  |  |  |  |  |  |  |  |  |  |  | 1.00 | -0.02 | 0.53 |
| **Si** |  |  |  |  |  |  |  |  |  |  |  |  |  | 1.00 | 0.06 |
| **Zn** |  |  |  |  |  |  |  |  |  |  |  |  |  |  | 1.00 |

**
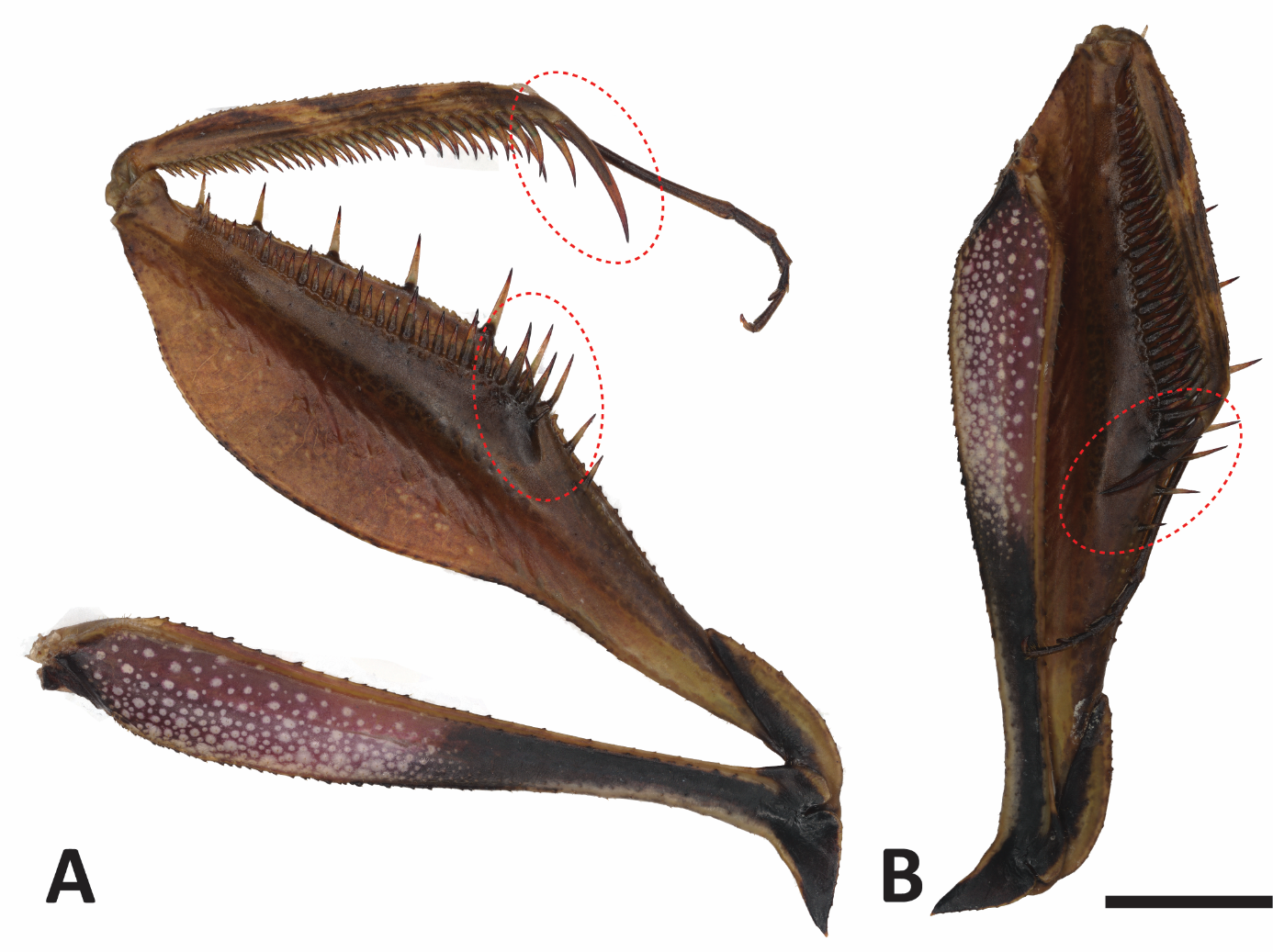
**

**Supplementary Figure 1.** Light microscopic images of the opened and closed foreleg of *Gongylus gongylodes*, specimen 11, female. High lightened regions interact during raptorial apparatus closure. A. Left foreleg, medial view, opened. B. Left foreleg, medial view, closed. Scale bar: 4 mm.


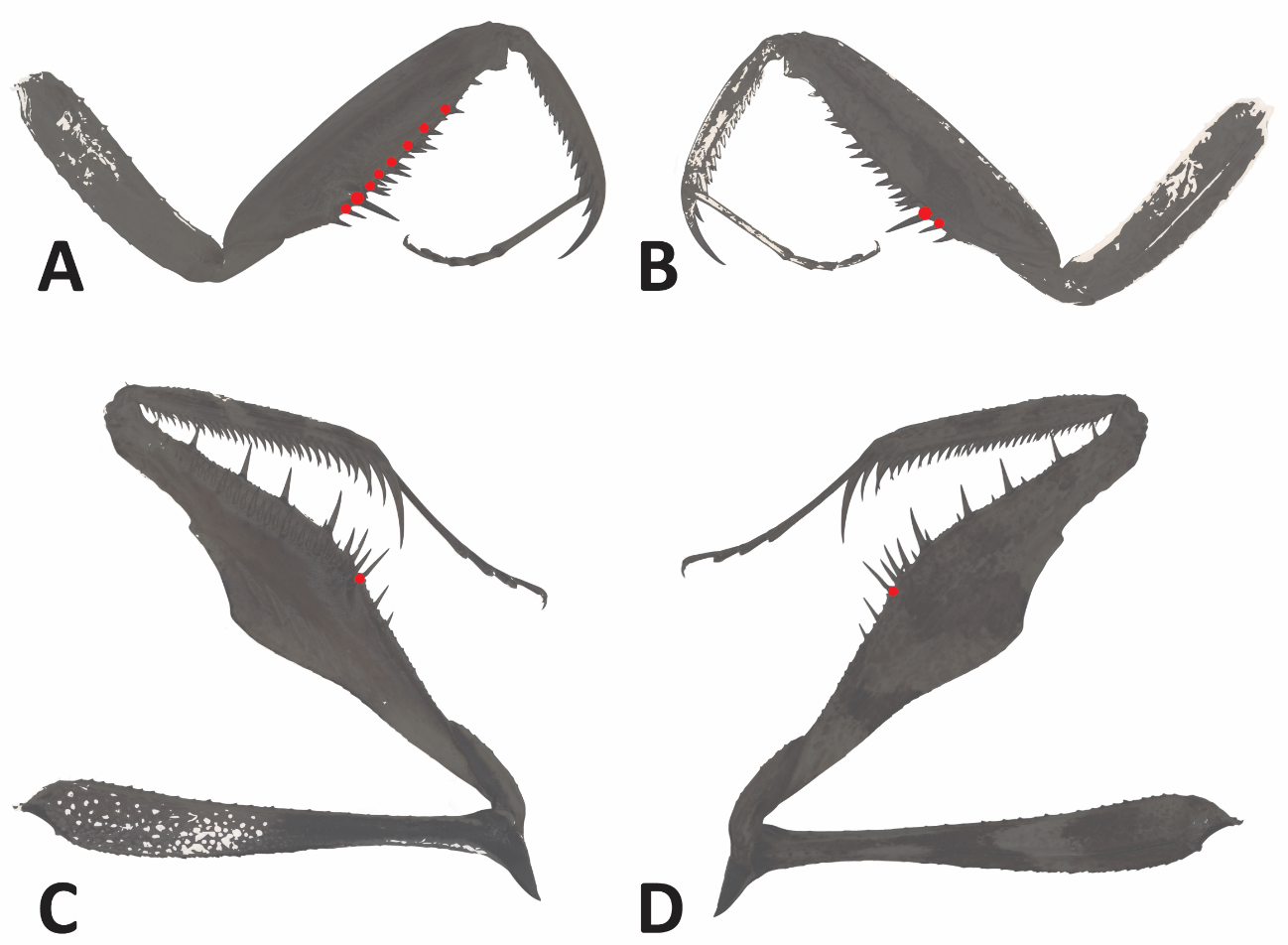


**Supplementary Figure 2.** Forelegs with high lightened movable spines (red dots). A–B. *Sphodromantis lineola*, specimen 01, male, left foreleg. A. Medial view. B. Lateral view. C–D. *Gongylus gongylodes*, specimen 22, male, left foreleg. C. Medial view. D. Lateral view.


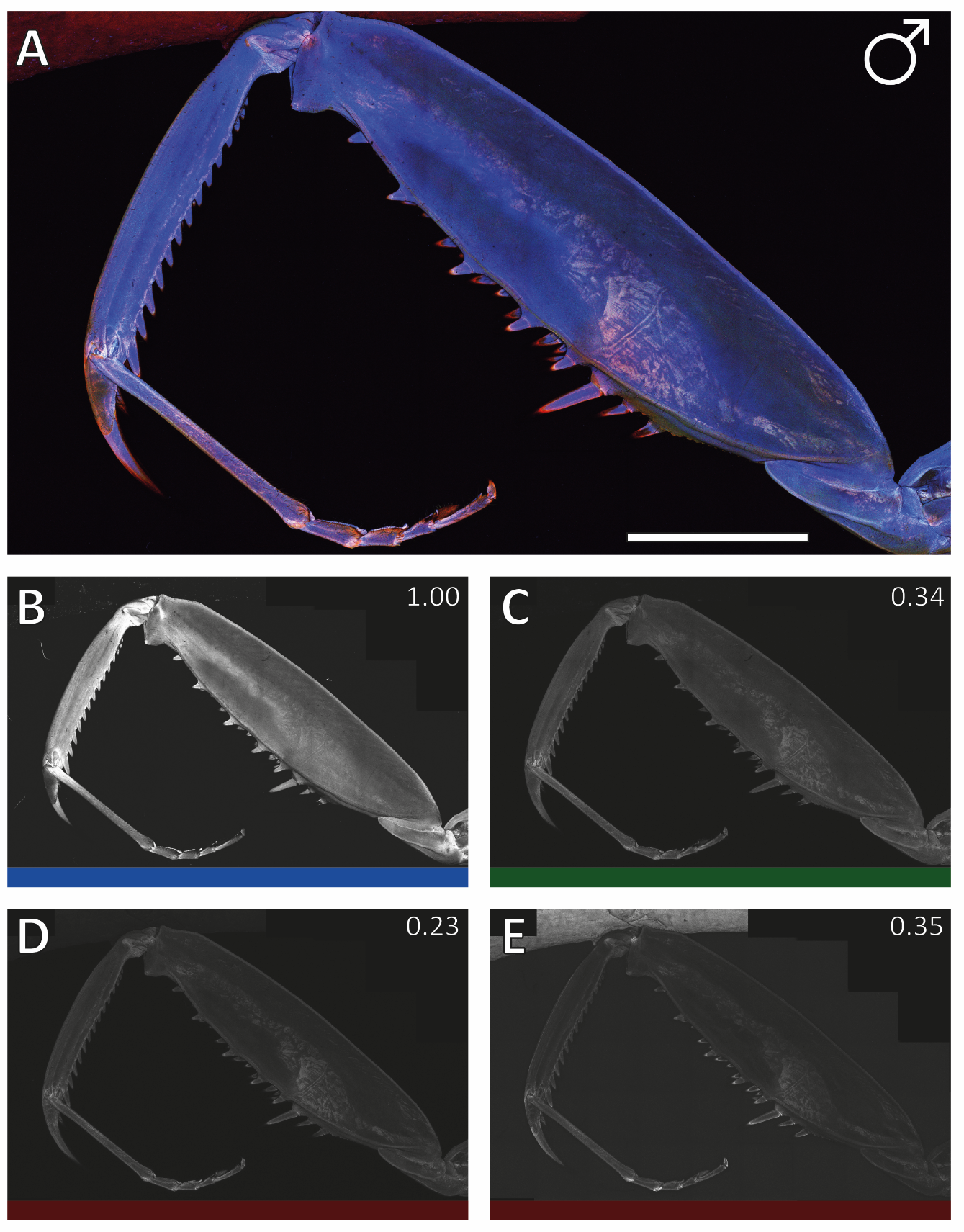


**Supplementary Figure 3.** *Sphodromantis lineola*, specimen 01, male, left foreleg, lateral view. CLSM image with the same settings as Figures 6 and 8 as well as Supplementary Figures 4–8. A. All channels. B. Blue (100%). C. Green (34% compared to blue). D. Red 50% (23% compared to blue). E. Red 50% (35% compared to blue). Scale bar: 4 mm.

**
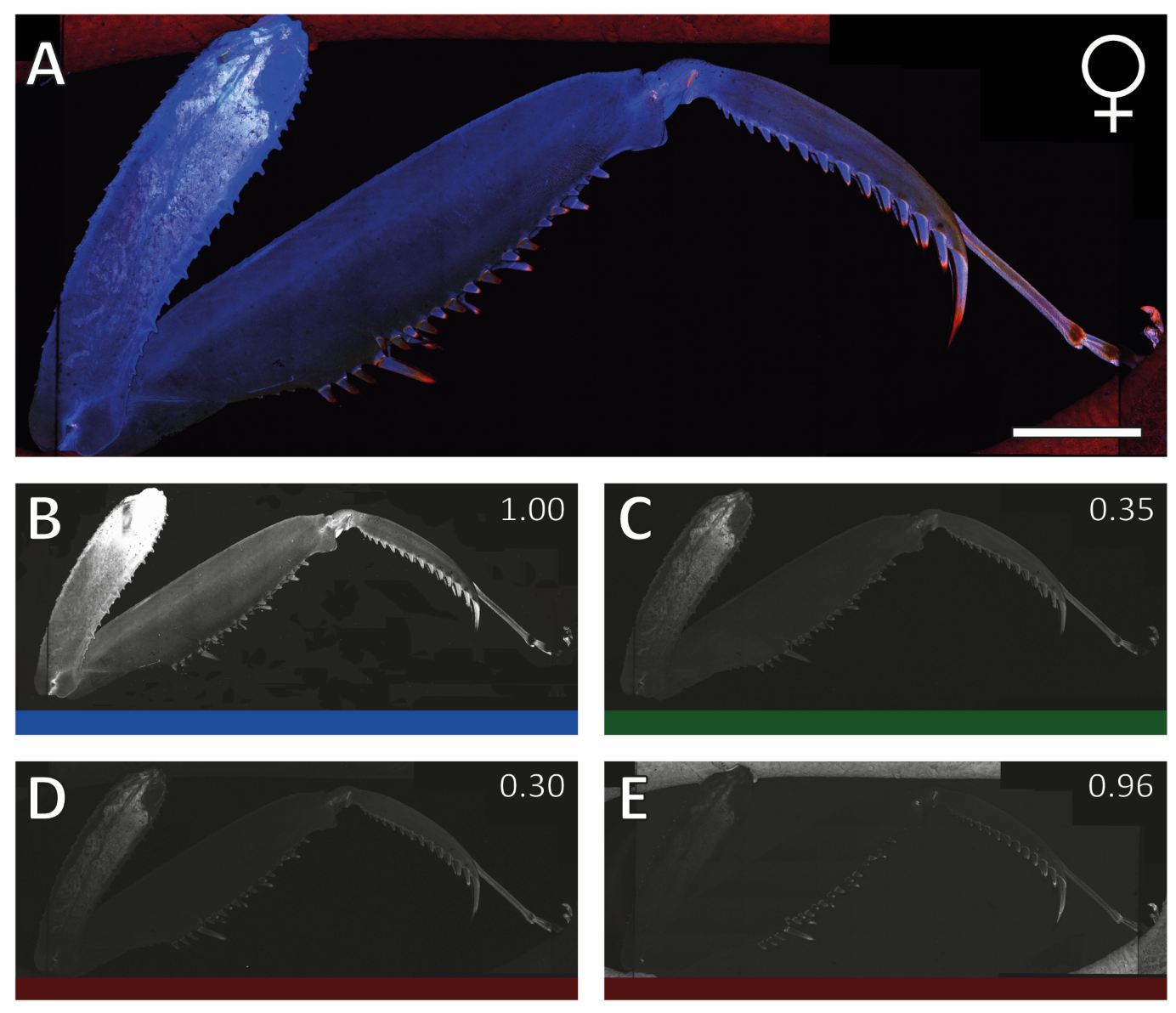
 Supplementary Figure 4.** *Sphodromantis lineola*, female, specimen 05, left foreleg, medial view. CLSM image with the same settings Figures 6 and 8 as well as Supplementary Figures 3 and 5–8. A. All channels. B. Blue (100%). C. Green (35% compared to blue). D. Red 50% (30% compared to blue). E. Red 50% (96% compared to blue). Scale bar: 4 mm.


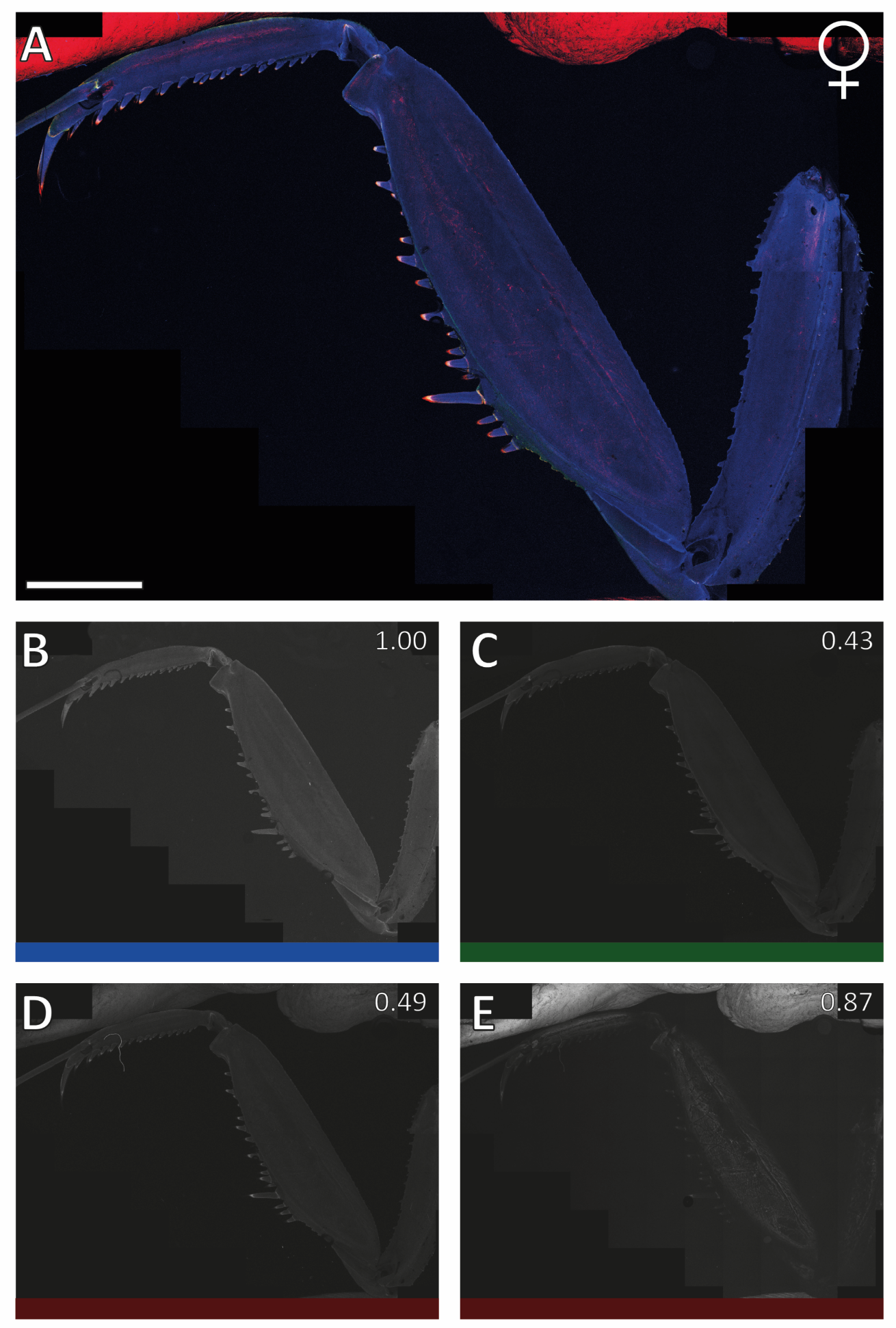


**Supplementary Figure 5.** *Sphodromantis lineola*, specimen 05, female, left foreleg, lateral view. CLSM image with the same settings as Figures 6 and 8 as well as Supplementary Figures 3–4 and 6–8. A. All channels. B. Blue (100%). C. Green (43% compared to blue). D. Red 50% (49% compared to blue). E. Red 50% (87% compared to blue). Scale bar: 4 mm.


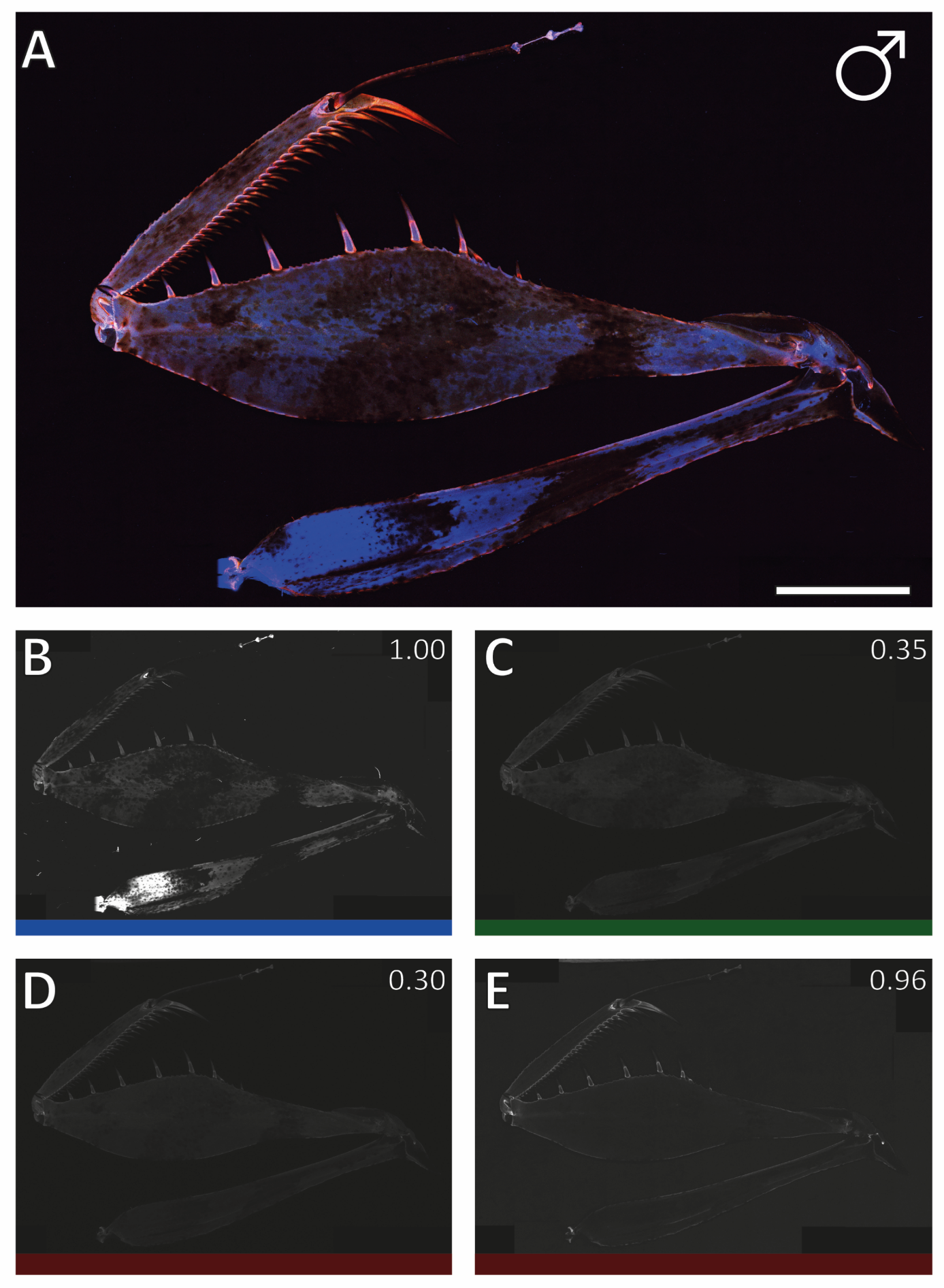


**Supplementary Figure 6.** *Gongylus gongylodes*, specimen 15, male, right foreleg, lateral view. CLSM image with the same settings as Figures 6 and 8 as well as Supplementary Figures 3–5 and 7–8. A. All channels. B. Blue (100%). C. Green (35% compared to blue). D. Red 50% (30% compared to blue). E. Red 50% (96% compared to blue). Scale bar: 4 mm.


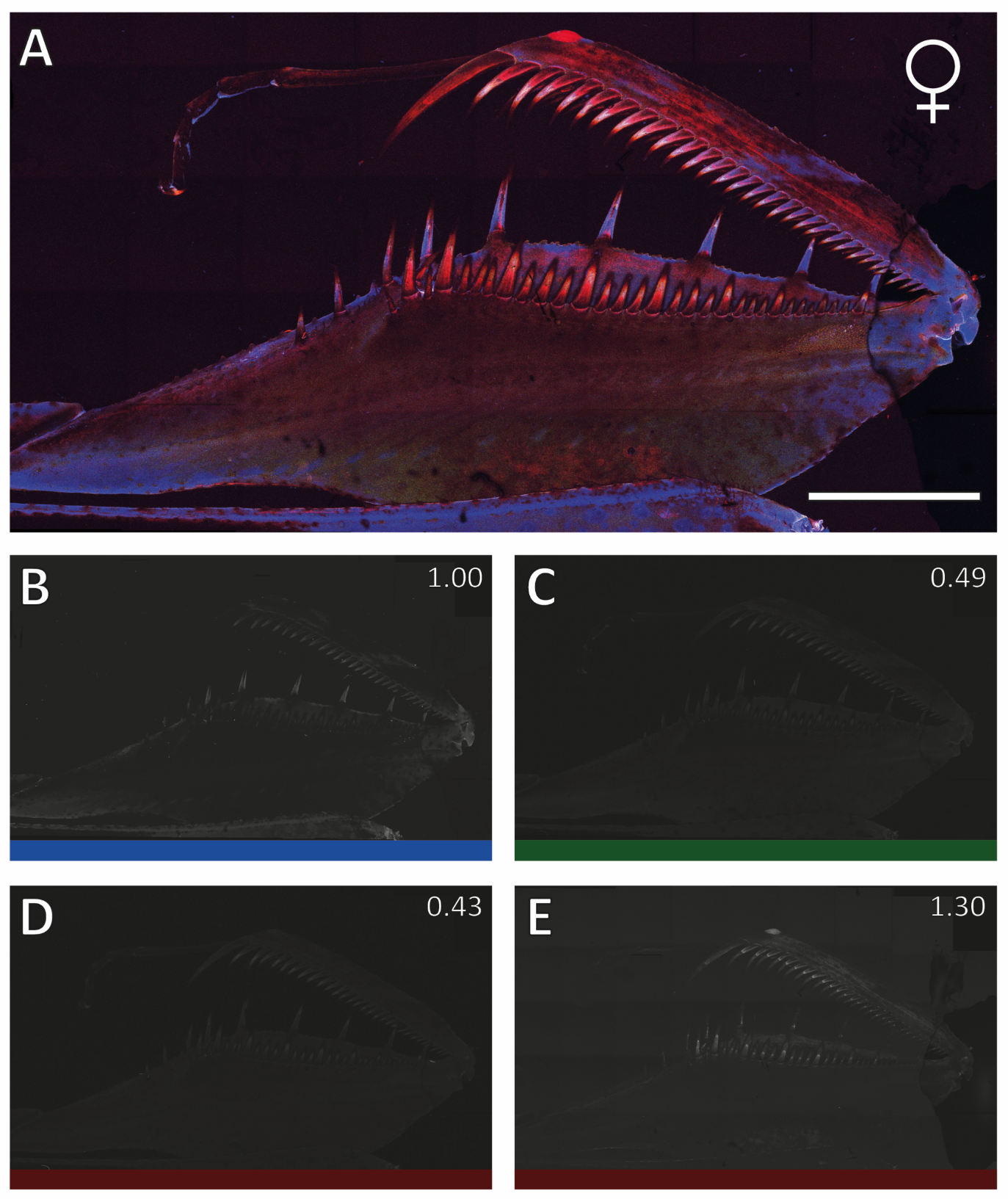


**Supplementary Figure 7.** *Gongylus gongylodes*, specimen 11, female, right foreleg, medial view. CLSM image with the same settings as Figures 6 and 8 as well as Supplementary Figures 3–6 and 7–8. A. All channels. B. Blue (100%). C. Green (49% compared to blue). D. Red 50% (43% compared to blue). E. Red 50% (130% compared to blue). Scale bar: 4 mm.


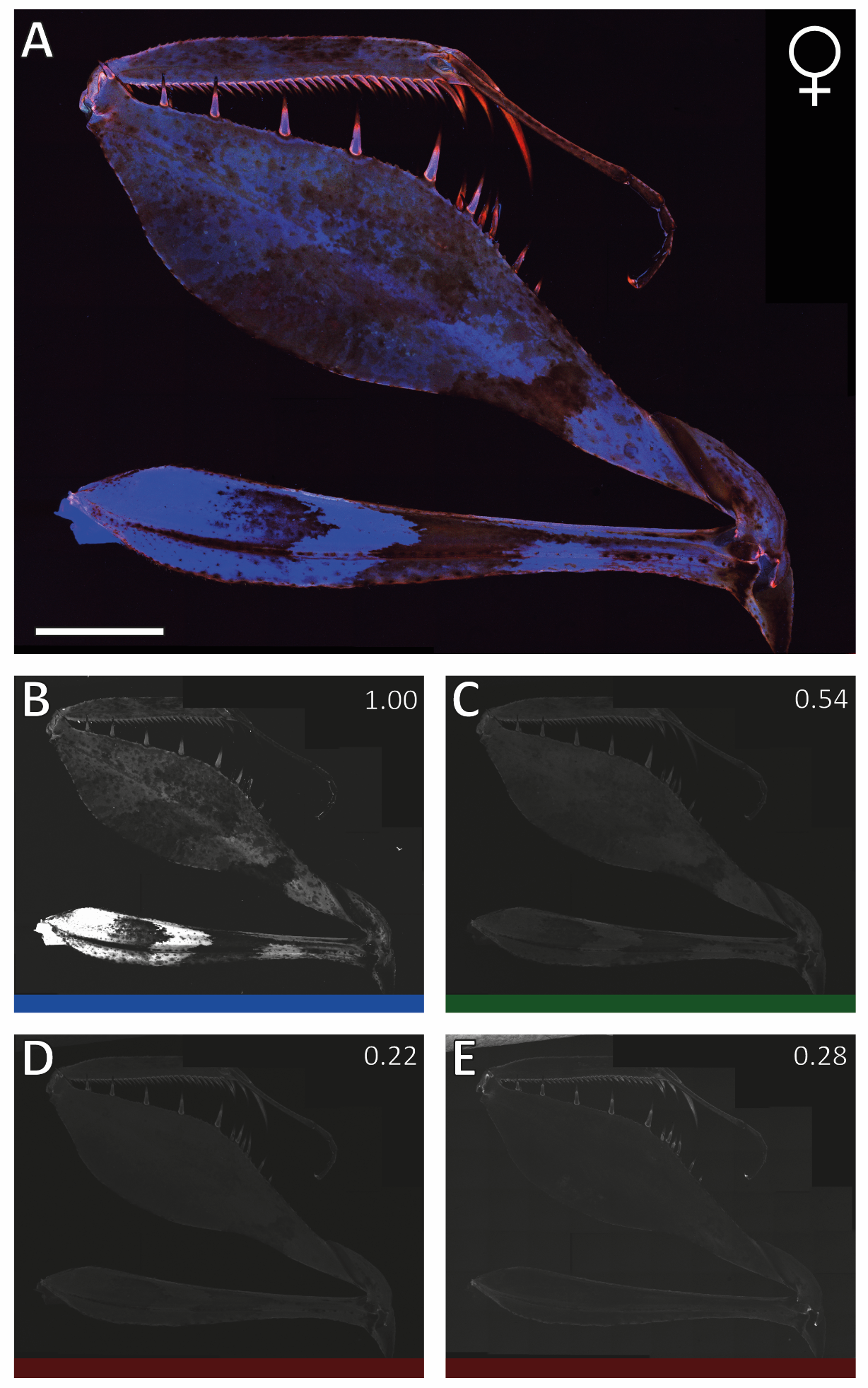


**Supplementary Figure 8.** *Gongylus gongylodes*, specimen 11, female, right foreleg, lateral view. CLSM image with the same settings as Figures 6 and 8 as well as Supplementary Figures 3–7. A. All channels. B. Blue (100%). C. Green (54% compared to blue). D. Red 50% (22% compared to blue). E. Red 50% (28% compared to blue). Scale bar: 4 mm.


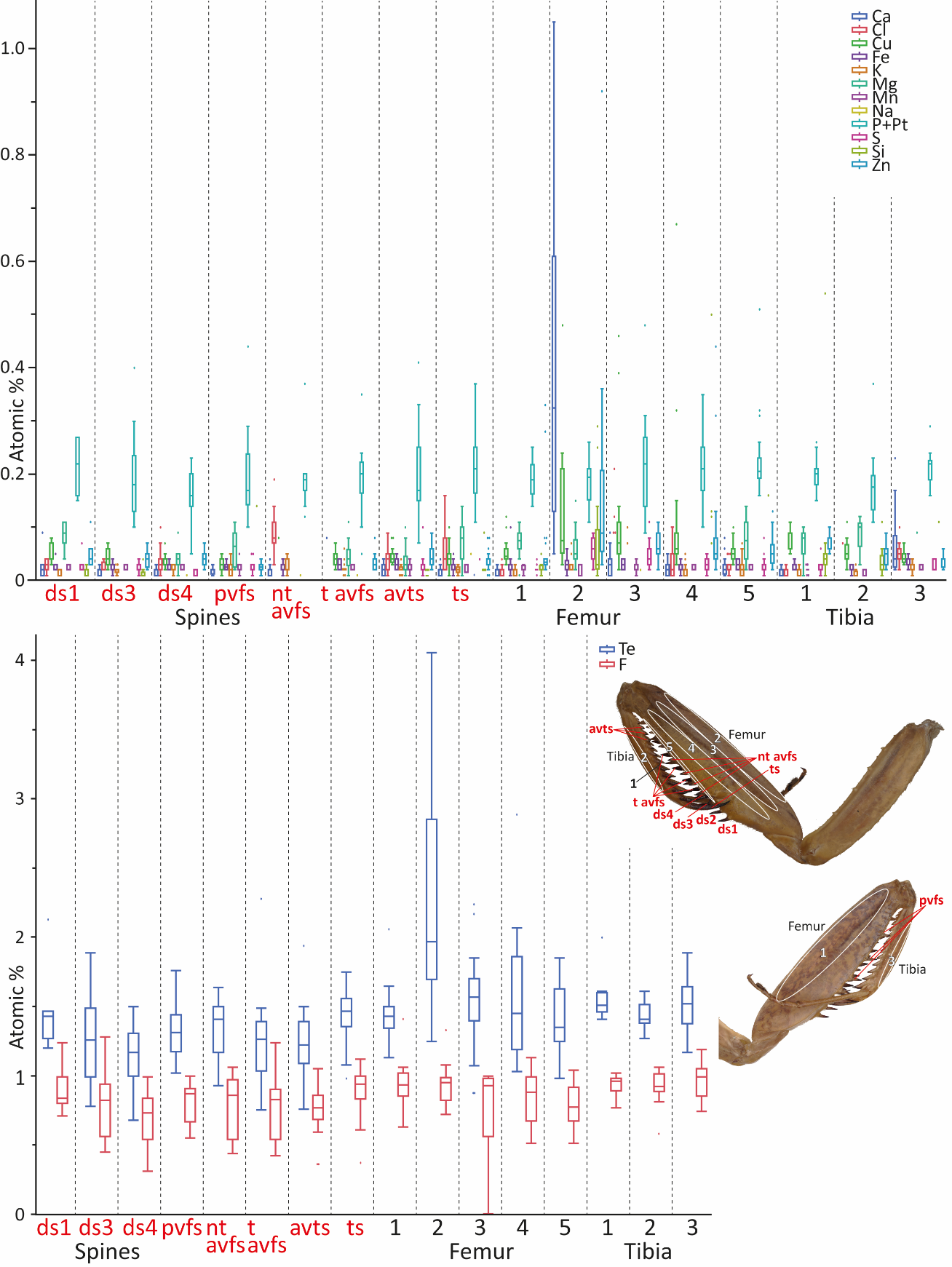


**Supplementary Figure 9.** Results from EDX analyses for *Sphodromantis lineola*, male, right foreleg, specimen 01 – sorted to the tested foreleg regions and spines. For values see Supplementary Table 7. Light microscopy images of the foreleg in medial view (above) and lateral view (below). Abbreviations: avts, anteroventral tibial spines; ds1, 1th discoidal spine; ds2, 2nd discoidal spine; ds3, 3rd discoidal spine; ds4, 4th discoidal spine; nt avfs, non-tiltable anteroventral femoral spines; pvfs, posteroventral femoral spines; t avfs, tiltable anteroventral femoral spines; Te, trace elements, sum of Ca, Cl, Cu, F, Fe, K, Mg, Mn, Na, P+Pt, S, Si, and Zn; ts, tibial spur.


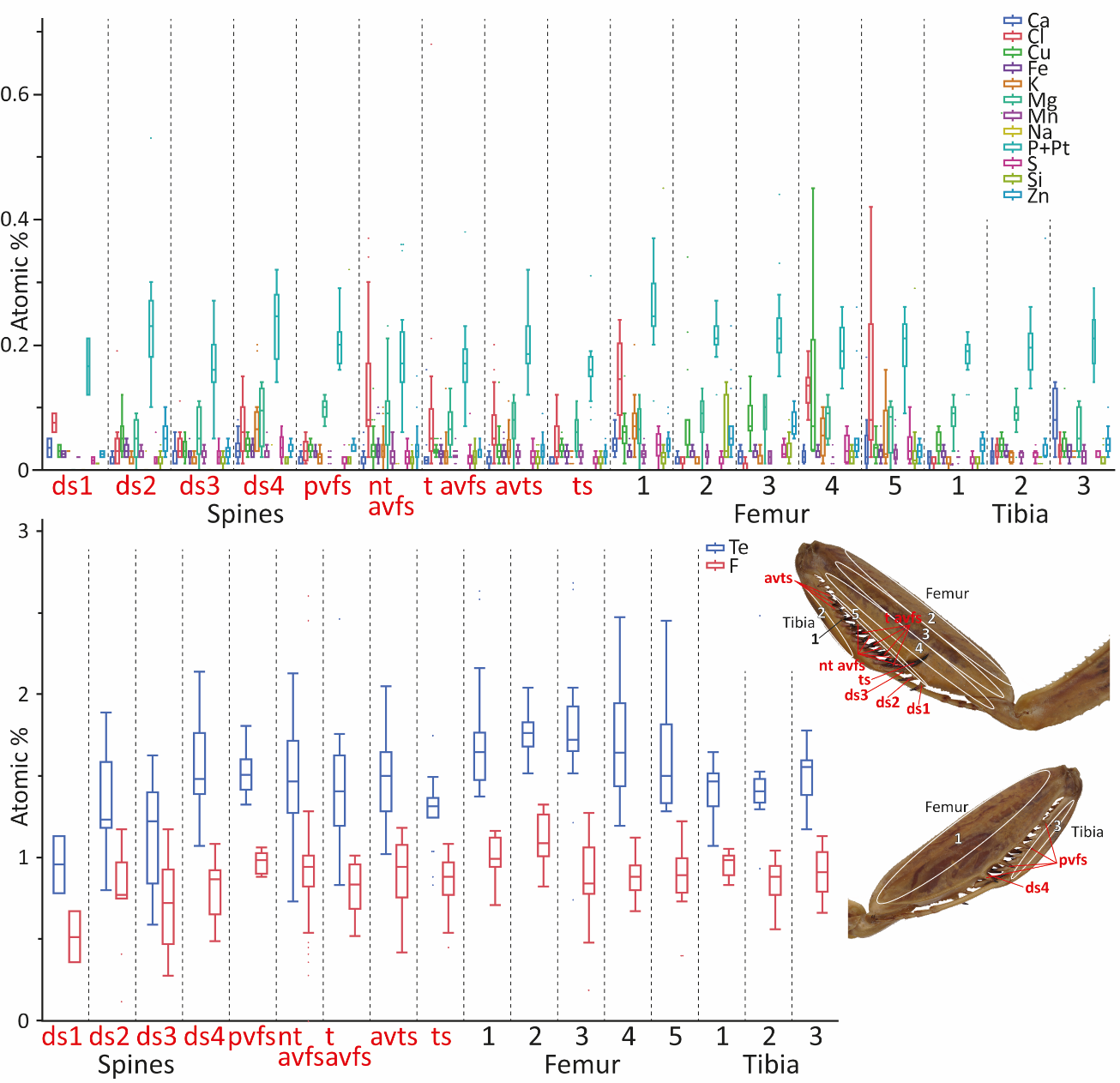


**Supplementary Figure 10.** Results from EDX analyses for *Sphodromantis lineola*, female, right foreleg, specimen 06 – sorted to the tested foreleg regions and spines. For values see Supplementary Table 7. Light microscopy images of the foreleg in medial view (above) and lateral view (below). Abbreviations: avts, anteroventral tibial spines; ds1, 1th discoidal spine; ds2, 2nd discoidal spine; ds3, 3rd discoidal spine; ds4, 4th discoidal spine; nt avfs, non-tiltable anteroventral femoral spines; pvfs, posteroventral femoral spines; t avfs, tiltable anteroventral femoral spines; Te, trace elements, sum of Ca, Cl, Cu, F, Fe, K, Mg, Mn, Na, P+Pt, S, Si, and Zn; ts, tibial spur.


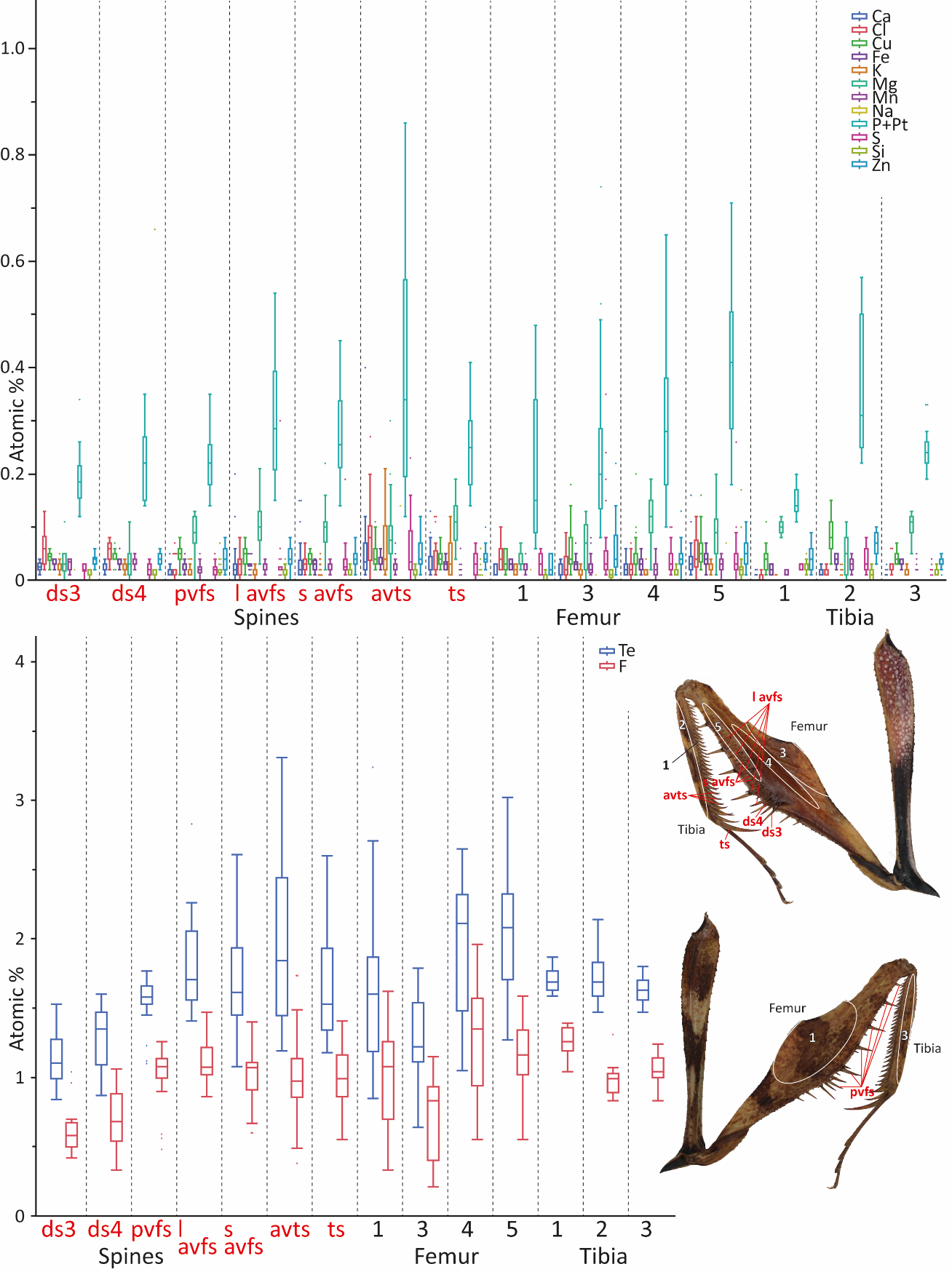


**Supplementary Figure 11.** Results from EDX analyses for *Gongylus gongylodes*, male, right foreleg, specimen 15 – sorted to the tested foreleg regions and spines. For values see Supplementary Table 7. Light microscopy images of the foreleg in medial view (above) and lateral view (below). Abbreviations: avts, anteroventral tibial spines; d3, 3rd discoidal spine; d4, 4th discoidal spine; l avfs, long anteroventral femoral spines; pvfs, posteroventral femoral spines; s avfs, short anteroventral femoral spines; Te, trace elements, sum of Ca, Cl, Cu, F, Fe, K, Mg, Mn, Na, P+Pt, S, Si, and Zn; ts, tibial spur.


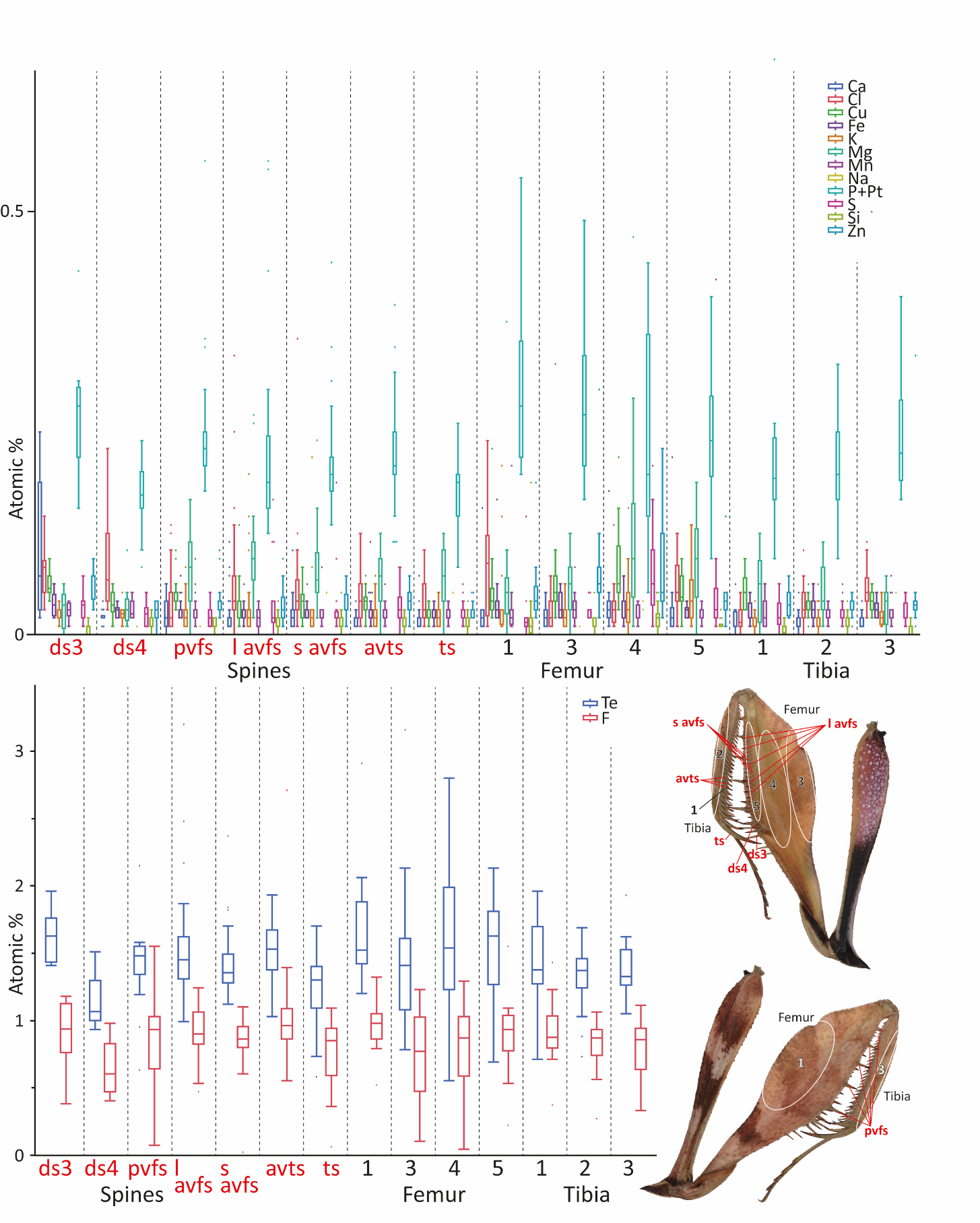


**Supplementary Figure 12.** Results from EDX analyses for *Gongylus gongylodes*, female, right foreleg, specimen 14 – sorted to the tested foreleg regions and spines. For values see Supplementary Table 7. Light microscopy images of the foreleg in medial view (above) and lateral view (below). Abbreviations: avts, anteroventral tibial spines; d3, 3rd discoidal spine; d4, 4th discoidal spine; l avfs, long anteroventral femoral spines; pvfs, posteroventral femoral spines; s avfs, short anteroventral femoral spines; Te, trace elements, sum of Ca, Cl, Cu, F, Fe, K, Mg, Mn, Na, P+Pt, S, Si, and Zn; ts, tibial spur.


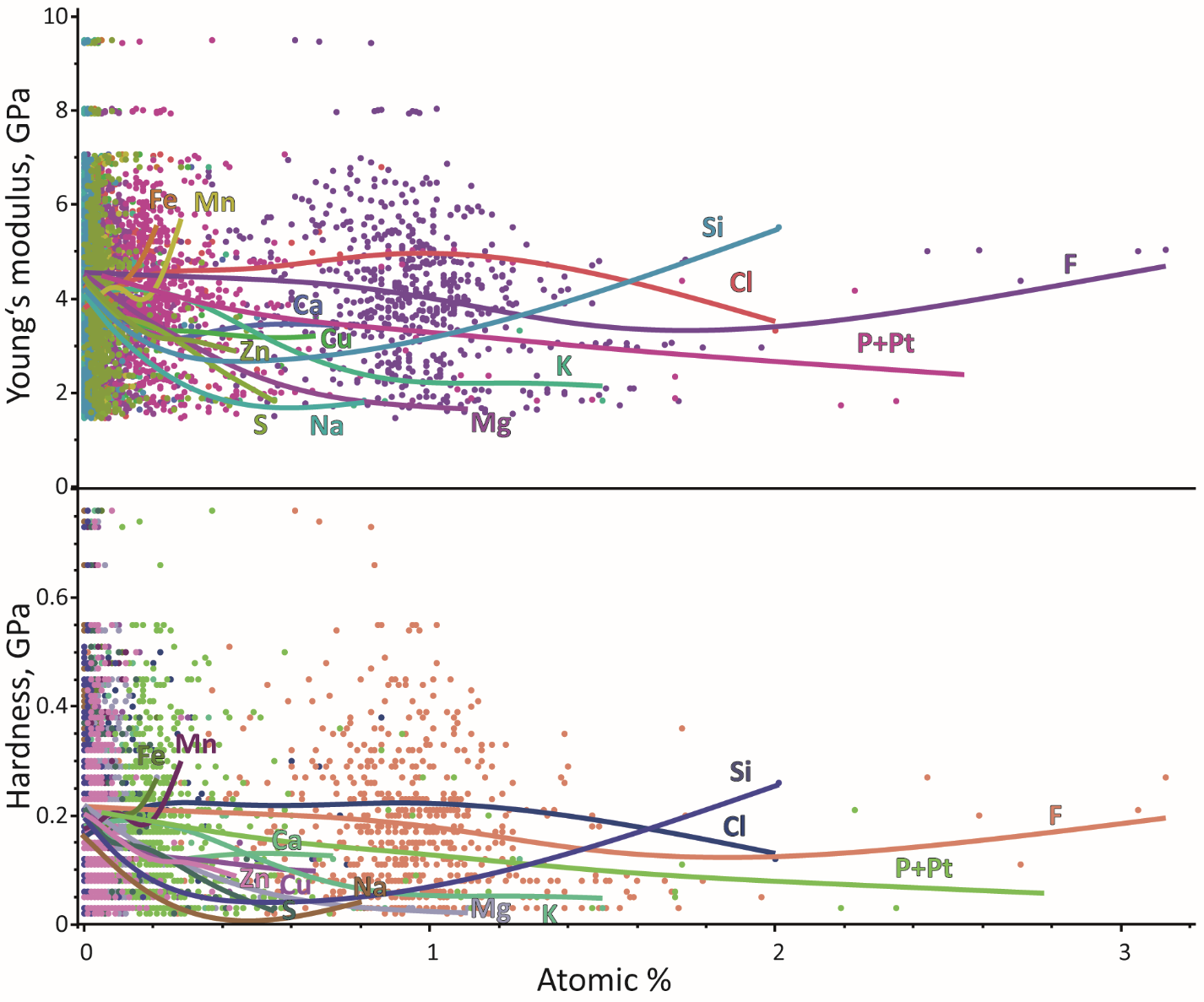


**Supplementary Figure 13.** Here, only the data of the regions of the forelegs, which were tested by both nanoindentation and elemental analysis, is shown. Each point represents one region, which has data on elements, given in atomic %, and the mechanical properties hardness and Young’s modulus, both given in GPa. By this, potential relationship between the elemental content and the mechanical properties can be seen.
